# Supplementary material for: Three-dimensional structural dynamics of DNA origami Bennett linkages using individual-particle electron tomography
Source: Nat Commun. 2018 Feb 9;9:592. doi: 10.1038/s41467-018-03018-0 (PMC5807444; doi:10.1038/s41467-018-03018-0)
Supplement: Supplementary file 1 — Supplementary Information [file 41467_2018_3018_MOESM1_ESM.pdf]

Supplementary Methods

Kinematic analysis of DNA origami Bennett linkage internal angles

Based on the dimensions of the DNA origami Bennett linkage and the kinematics model (was described in ref. [2, 20, 34], figure of kinematics model was also reprinted here in Supporting **Figure S67**), we can obtain the position of four vertex of Bennett linkage (**A**, **B** ,**C** and **D**) as:

$$\mathbf{A}=\begin{Bmatrix}0\\0\\0\end{Bmatrix},\ \mathbf{B}=\begin{Bmatrix}l\\0\\0\end{Bmatrix},\ \mathbf{C}=\mathbf{B}+[e^{\theta_2\mathbf{S}_2}]\begin{Bmatrix}-l\\0\\0\end{Bmatrix},\ \mathbf{D}=[e^{\theta_1\mathbf{S}_1}]\begin{Bmatrix}l\\0\\0\end{Bmatrix}, \tag{1}$$

in which l is the length of Bennett linkage arm (27.8 nm), $\mathbf{S_1}$ and $\mathbf{S_2}$ are two internal rotational axis ($\mathbf{S}_1=\{-0.544984,-0.734471,-0.404406\}$ and $\mathbf{S}_2=\{-0.544984,0.404406,-0.734471\}$), θ_1 is the rotation angle of edge AD against edge AB along $\mathbf{S_1}$, and θ_2 is the rotation angle of edge BC against edge AB along $\mathbf{S_2}$.

Since the Bennett linkage should be a closed-loop chain, the distance between vertex C and D should be l, namely:

$$(\mathbf{D}-\mathbf{C})\cdot(\mathbf{D}-\mathbf{C})=l^2 \tag{2}$$

Based on equation (1) and the above distance constraint (2), we can calculate θ_2 against each θ_1 value.

Based on the geometry of Bennett linkage, we can also determine the angles α, β between two nearby edges (for details please refer ref. 31) from each set of θ_1 and θ_2 as:

$$\sin(\alpha/2)=\sin(\eta)\sin(\theta_1/2), \tag{3}$$

and

$$\sin(\beta/2)=\sin(\xi)\sin(\theta_2/2), \tag{4}$$

in which η is the angle between **S1** and edge AB (123˚), and ξ is the angle between S2 and edge BC (57˚).

By assuming θ_1 is distributed evenly, the distribution of α and β can then be obtained..

Based on the cross product of two nearby edges, we determined the normal direction of planes formed by any two adjacent edges. The angles between normal directions were then measured as the dihedral angles (θ and φ) between planes.

Supplementary Figures

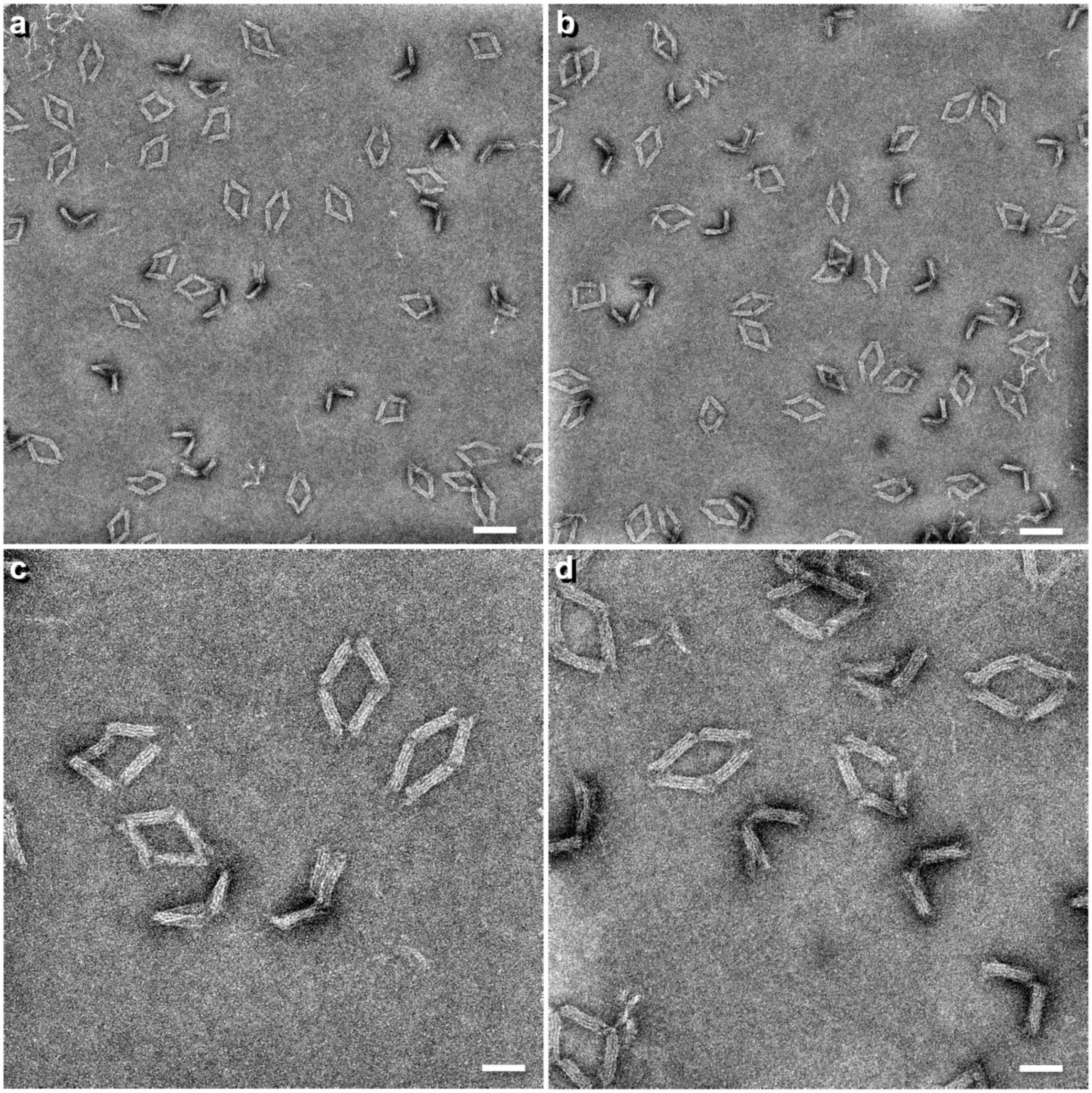

Supplementary Fig. 1 | Additional OpNS-EM images of DNA origami Bennett linkage (a and b) Imaged under 80kX magnification. **(c and d)** Imaged under 125kX magnification. Scale bars are 50 nm in a and b, and are 20 nm in c and d.

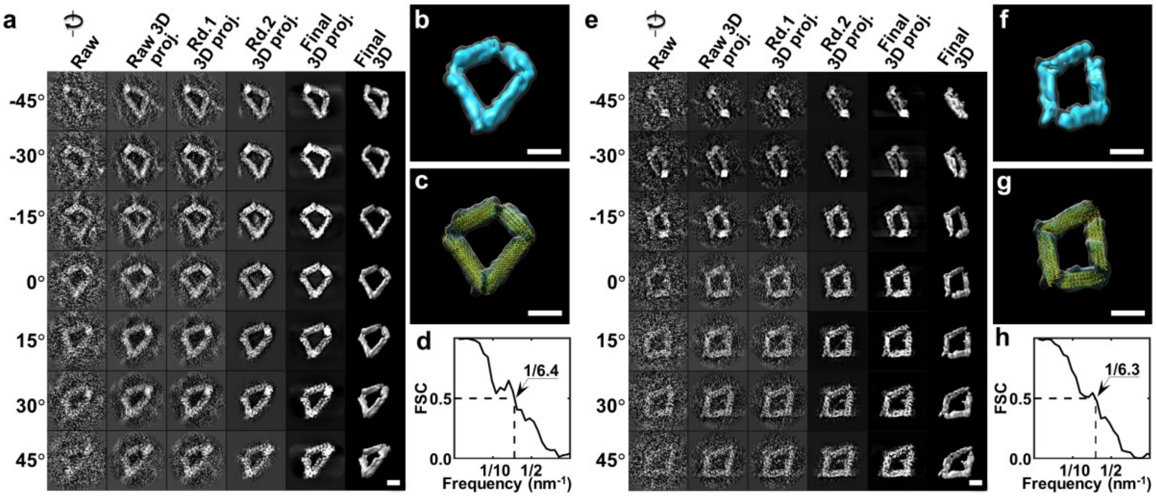

Supplementary Fig. 2 | IPET reconstruction processes on the 1st and 2nd DNA origami Bennett linkage (a) Seven representative tilt views (first column), their corresponding projections on the intermediate 3D reconstructions from major iterations (second to fifth columns), and the final 3D density map (sixth column) of the 1st particle of the DNA origami Bennett linkage are shown. **(b)** The final 3D density map. **(c)** The density map displays the overall conformation of the DNA origami Bennett linkage. A new conformation of DNA origami Bennett linkage was obtained (yellow ribbon) by flexible docking the Bennett linkage model into the density. **(d)** The FSC curve shows that the resolution of the final 3D reconstruction was ~6.4 nm. **(e–h)** The 3D density map of the 2nd individual Bennett linkage was reconstructed from the tilt images using IPET. The FSC analysis showed that the 3D reconstruction resolution was ~6.3 nm. Scale bars are 20 nm.

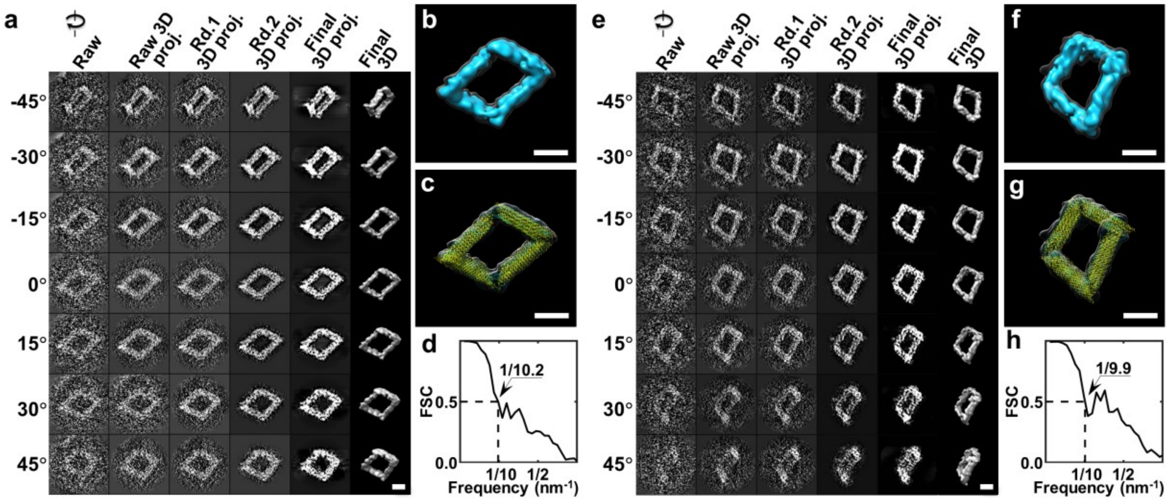

Supplementary Fig. 3 | IPET reconstruction processes on the 3rd and 4th DNA origami Bennett linkage (a) Seven representative tilt views (first column), their corresponding projections on the intermediate 3D reconstructions from major iterations (second to fifth columns), and the final 3D density map (sixth column) of the 3rd particle of the DNA origami Bennett linkage are shown. **(b)** The final 3D density map. **(c)** The density map displays the overall conformation of the DNA origami Bennett linkage. A new conformation of DNA origami Bennett linkage was obtained (yellow ribbon) by flexible docking the Bennett linkage model into the density. **(d)** The FSC curve shows that the resolution of the final 3D reconstruction was ~10.2 nm. **(e–h)** The 3D density map of the 4th individual Bennett linkage was reconstructed from the tilt images using IPET. The FSC analysis showed that the 3D reconstruction resolution was ~9.9 nm. Scale bars are 20 nm.

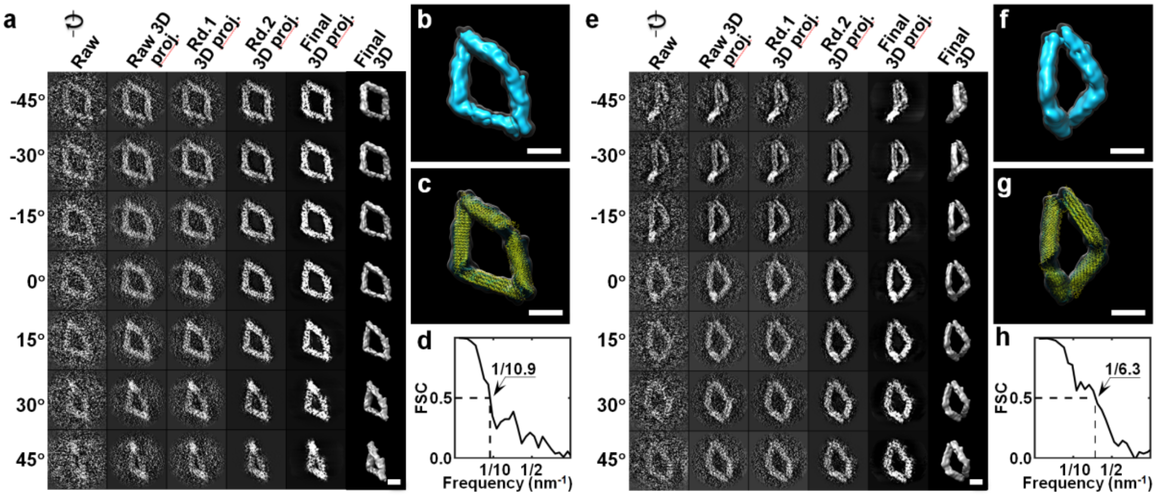

Supplementary Fig. 4 | IPET reconstruction processes on the 5th and 6th DNA origami Bennett linkage (a) Seven representative tilt views (first column), their corresponding projections on the intermediate 3D reconstructions from major iterations (second to fifth columns), and the final 3D density map (sixth column) of the 5th particle of the DNA origami Bennett linkage are shown. **(b)** The final 3D density map. **(c)** The density map displays the overall conformation of the DNA origami Bennett linkage. A new conformation of DNA origami Bennett linkage was obtained (yellow ribbon) by flexible docking the Bennett linkage model into the density. **(d)** The FSC curve shows that the resolution of the final 3D reconstruction was ~10.9 nm. **(e–h)** The 3D density map of the 6th individual Bennett linkage was reconstructed from the tilt images using IPET. The FSC analysis showed that the 3D reconstruction resolution was ~6.3 nm. Scale bars are 20 nm.

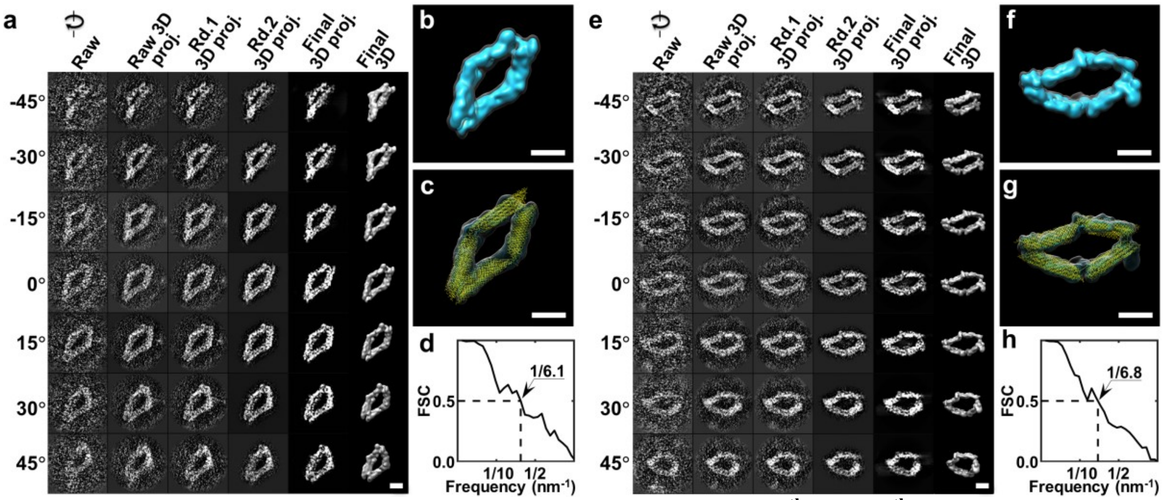

Supplementary Fig. 5 | IPET reconstruction processes on the 7th and 8th DNA origami Bennett linkage (a) Seven representative tilt views (first column), their corresponding projections on the intermediate 3D reconstructions from major iterations (second to fifth columns), and the final 3D density map (sixth column) of the 7th particle of the DNA origami Bennett linkage are shown. **(b)** The final 3D density map. **(c)** The density map displays the overall conformation of the DNA origami Bennett linkage. A new conformation of DNA origami Bennett linkage was obtained (yellow ribbon) by flexible docking the Bennett linkage model into the density. **(d)** The FSC curve shows that the resolution of the final 3D reconstruction was ~6.1 nm. **(e–h)** The 3D density map of the 8th individual Bennett linkage was reconstructed from the tilt images using IPET. The FSC analysis showed that the 3D reconstruction resolution was ~6.8 nm. Scale bars are 20 nm.

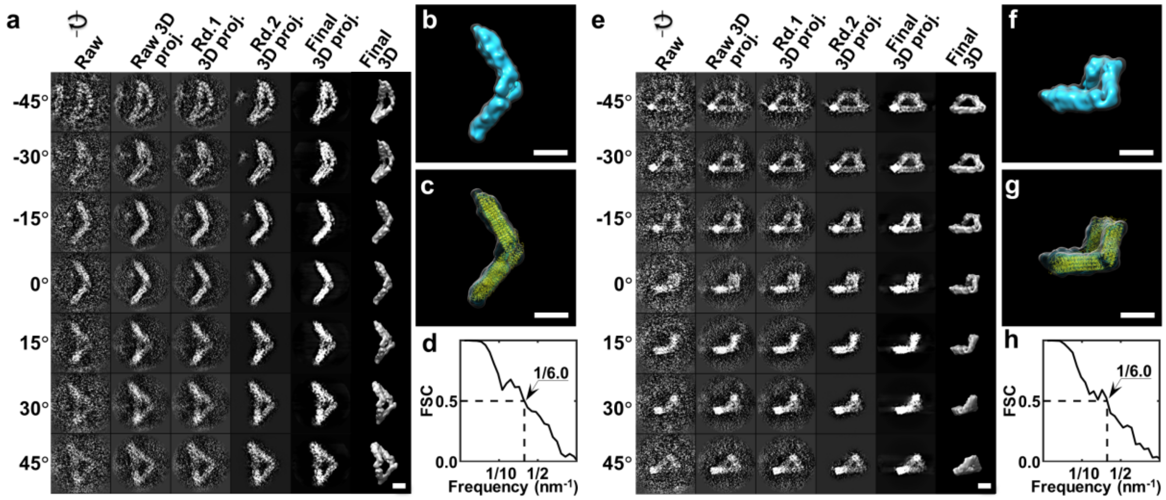

Supplementary Fig. 6 | IPET reconstruction processes on the 9th and 10th DNA origami Bennett linkage (a) Seven representative tilt views (first column), their corresponding projections on the intermediate 3D reconstructions from major iterations (second to fifth columns), and the final 3D density map (sixth column) of the 9th particle of the DNA origami Bennett linkage are shown. **(b)** The final 3D density map. **(c)** The density map displays the overall conformation of the DNA origami Bennett linkage. A new conformation of DNA origami Bennett linkage was obtained (yellow ribbon) by flexible docking the Bennett linkage model into the density. **(d)** The FSC curve shows that the resolution of the final 3D reconstruction was ~6.0 nm. **(e–h)** The 3D density map of the 10th individual Bennett linkage was reconstructed from the tilt images using IPET. The FSC analysis showed that the 3D reconstruction resolution was ~6.0 nm. Scale bars are 20 nm.

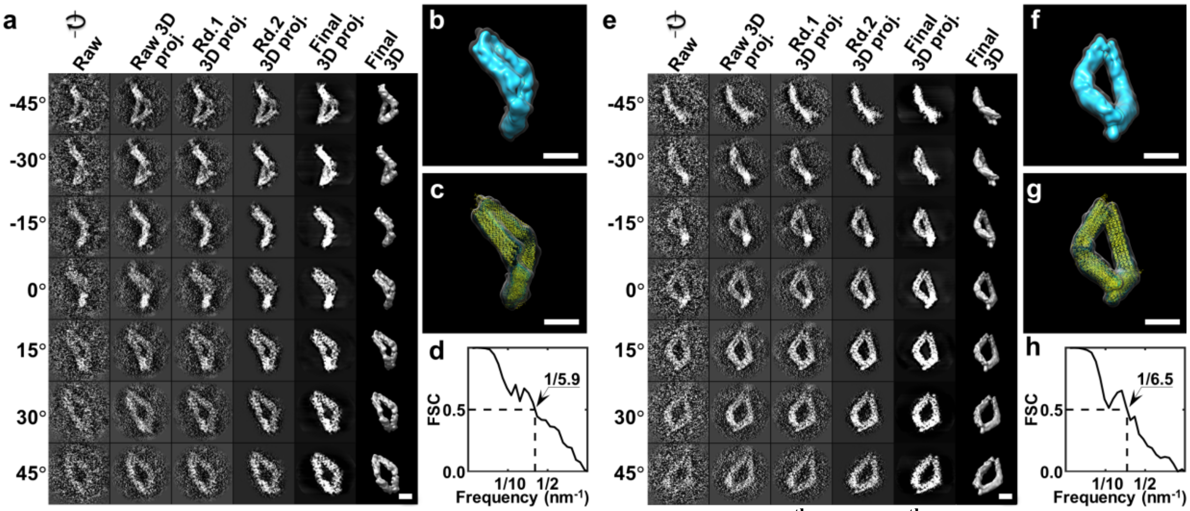

Supplementary Fig. 7 | IPET reconstruction processes on the 11th and 12th DNA origami Bennett linkage (a) Seven representative tilt views (first column), their corresponding projections on the intermediate 3D reconstructions from major iterations (second to fifth columns), and the 11th 3D density map (sixth column) of the 11th particle of the DNA origami Bennett linkage are shown. **(b)** The final 3D density map. **(c)** The density map displays the overall conformation of the DNA origami Bennett linkage. A new conformation of DNA origami Bennett linkage was obtained (yellow ribbon) by flexible docking the Bennett linkage model into the density. **(d)** The FSC curve shows that the resolution of the final 3D reconstruction was ~5.9 nm. **(e–h)** The 3D density map of the 12th individual Bennett linkage was reconstructed from the tilt images using IPET. The FSC analysis showed that the 3D reconstruction resolution was ~6.5 nm. Scale bars are 20 nm.

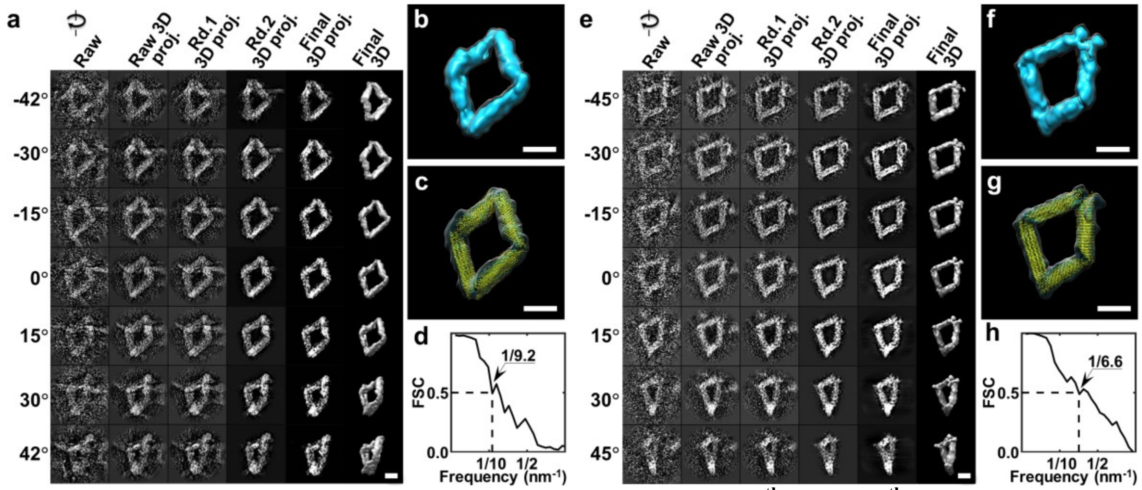

Supplementary Fig. 8 | IPET reconstruction processes on the 13th and 14th DNA origami Bennett linkage (a) Seven representative tilt views (first column), their corresponding projections on the intermediate 3D reconstructions from major iterations (second to fifth columns), and the 13th 3D density map (sixth column) of the 13th particle of the DNA origami Bennett linkage are shown. **(b)** The final 3D density map. **(c)** The density map displays the overall conformation of the DNA origami Bennett linkage. A new conformation of DNA origami Bennett linkage was obtained (yellow ribbon) by flexible docking the Bennett linkage model into the density. **(d)** The FSC curve shows that the resolution of the final 3D reconstruction was ~9.2 nm. **(e–h)** The 3D density map of the 14th individual Bennett linkage was reconstructed from the tilt images using IPET. The FSC analysis showed that the 3D reconstruction resolution was ~6.6 nm. Scale bars are 20 nm.

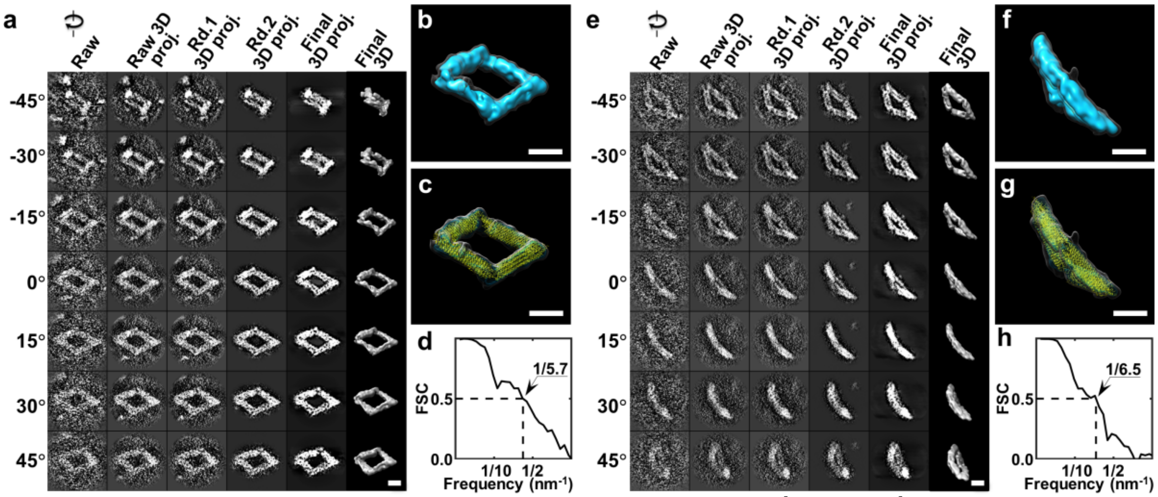

Supplementary Fig. 9 | IPET reconstruction processes on the 15th and 16th DNA origami Bennett linkage (a) Seven representative tilt views (first column), their corresponding projections on the intermediate 3D reconstructions from major iterations (second to fifth columns), and the 3D density map (sixth column) of the 15th particle of the DNA origami Bennett linkage are shown. **(b)** The final 3D density map. **(c)** The density map displays the overall conformation of the DNA origami Bennett linkage. A new conformation of DNA origami Bennett linkage was obtained (yellow ribbon) by flexible docking the Bennett linkage model into the density. **(d)** The FSC curve shows that the resolution of the final 3D reconstruction was ~5.7 nm. **(e–h)** The 3D density map of the 16th individual Bennett linkage was reconstructed from the tilt images using IPET. The FSC analysis showed that the 3D reconstruction resolution was ~6.5 nm. Scale bars are 20 nm.

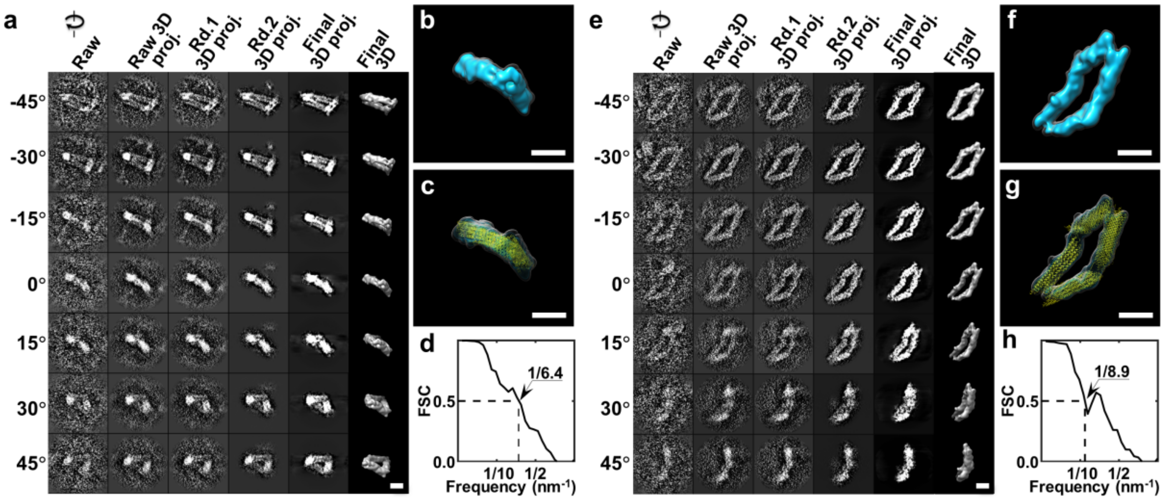

Supplementary Fig. 10 | IPET reconstruction processes on the 17th and 18th DNA origami Bennett linkage (a) Seven representative tilt views (first column), their corresponding projections on the intermediate 3D reconstructions from major iterations (second to fifth columns), and the 3D density map (sixth column) of the 17th particle of the DNA origami Bennett linkage are shown. **(b)** The final 3D density map. **(c)** The density map displays the overall conformation of the DNA origami Bennett linkage. A new conformation of DNA origami Bennett linkage was obtained (yellow ribbon) by flexible docking the Bennett linkage model into the density. **(d)** The FSC curve shows that the resolution of the final 3D reconstruction was ~6.4 nm. **(e–h)** The 3D density map of the 18th individual Bennett linkage was reconstructed from the tilt images using IPET. The FSC analysis showed that the 3D reconstruction resolution was ~8.9 nm. Scale bars are 20 nm.

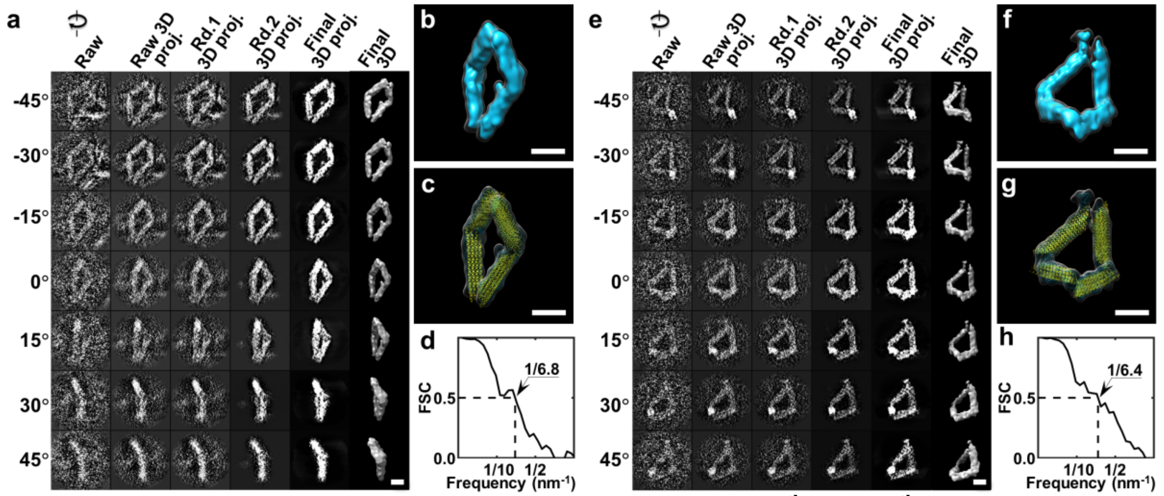

Supplementary Fig. 11 | IPET reconstruction processes on the 19th and 20th DNA origami Bennett linkage (a) Seven representative tilt views (first column), their corresponding projections on the intermediate 3D reconstructions from major iterations (second to fifth columns), and the 3D density map (sixth column) of the 19th particle of the DNA origami Bennett linkage are shown. **(b)** The final 3D density map. **(c)** The density map displays the overall conformation of the DNA origami Bennett linkage. A new conformation of DNA origami Bennett linkage was obtained (yellow ribbon) by flexible docking the Bennett linkage model into the density. **(d)** The FSC curve shows that the resolution of the final 3D reconstruction was ~6.8 nm. **(e–h)** The 3D density map of the 20th individual Bennett linkage was reconstructed from the tilt images using IPET. The FSC analysis showed that the 3D reconstruction resolution was ~6.4 nm. Scale bars are 20 nm.

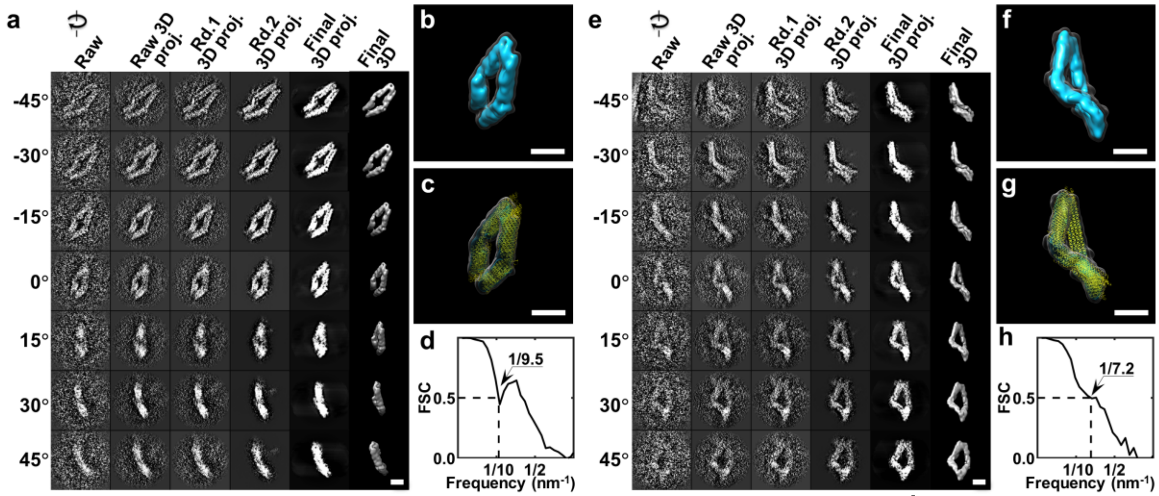

Supplementary Fig. 12 | IPET reconstruction processes on the 21st and 22nd DNA origami Bennett linkage (a) Seven representative tilt views (first column), their corresponding projections on the intermediate 3D reconstructions from major iterations (second to fifth columns), and the 3D density map (sixth column) of the 21st particle of the DNA origami Bennett linkage are shown. **(b)** The final 3D density map. **(c)** The density map displays the overall conformation of the DNA origami Bennett linkage. A new conformation of DNA origami Bennett linkage was obtained (yellow ribbon) by flexible docking the Bennett linkage model into the density. **(d)** The FSC curve shows that the resolution of the final 3D reconstruction was ~9.5 nm. **(e–h)** The 3D density map of the 22nd individual Bennett linkage was reconstructed from the tilt images using IPET. The FSC analysis showed that the 3D reconstruction resolution was ~7.2 nm. Scale bars are 20 nm.

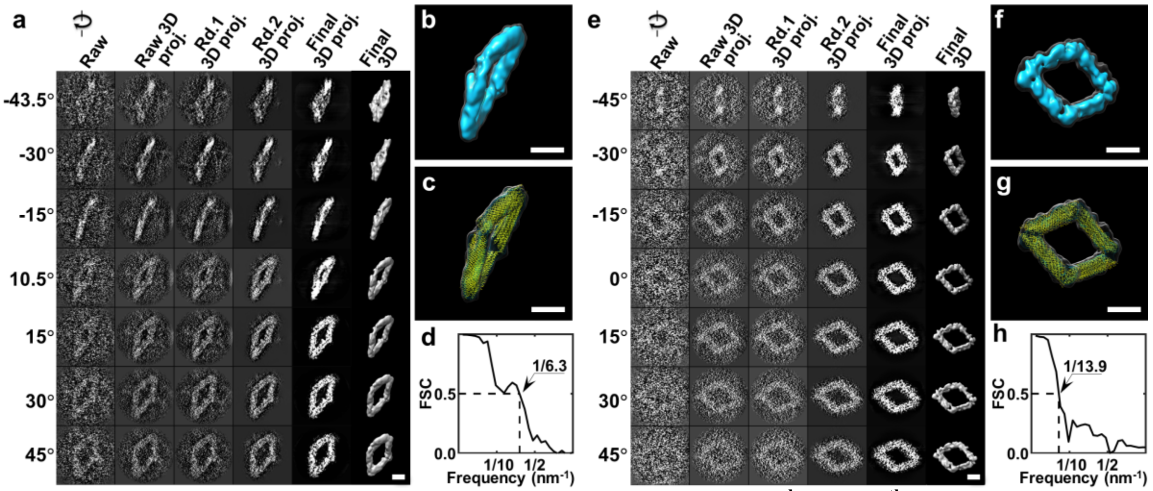

Supplementary Fig. 13 | IPET reconstruction processes on the 23rd and 24th DNA origami Bennett linkage (a) Seven representative tilt views (first column), their corresponding projections on the intermediate 3D reconstructions from major iterations (second to fifth columns), and the 3D density map (sixth column) of the 23rd particle of the DNA origami Bennett linkage are shown. **(b)** The final 3D density map. **(c)** The density map displays the overall conformation of the DNA origami Bennett linkage. A new conformation of DNA origami Bennett linkage was obtained (yellow ribbon) by flexible docking the Bennett linkage model into the density. **(d)** The FSC curve shows that the resolution of the final 3D reconstruction was ~6.3 nm. **(e–h)** The 3D density map of the 24th individual Bennett linkage was reconstructed from the tilt images using IPET. The FSC analysis showed that the 3D reconstruction resolution was ~13.9 nm. Scale bars are 20 nm.

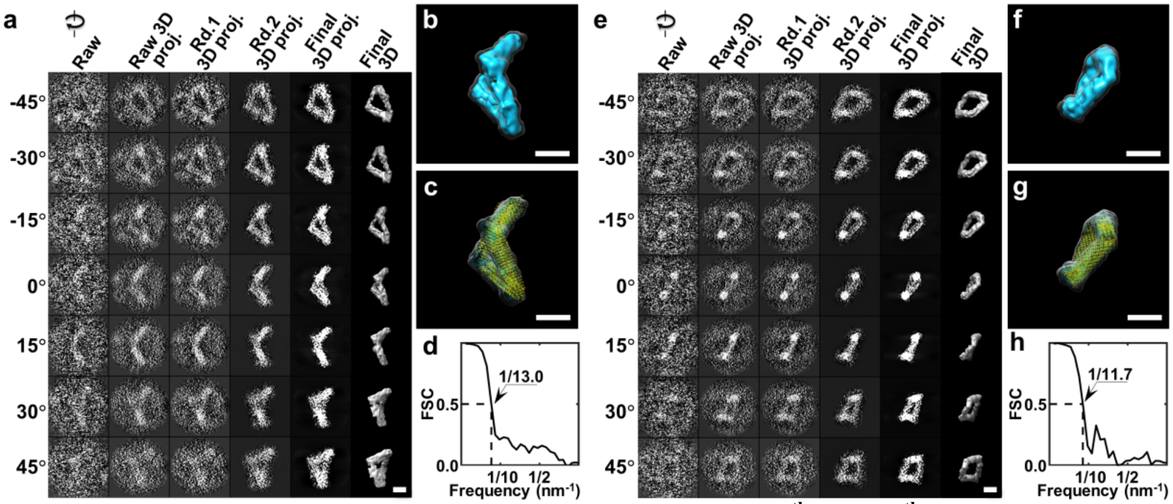

Supplementary Fig. 14 | IPET reconstruction processes on the 25th and 26th DNA origami Bennett linkage (a) Seven representative tilt views (first column), their corresponding projections on the intermediate 3D reconstructions from major iterations (second to fifth columns), and the 3D density map (sixth column) of the 25th particle of the DNA origami Bennett linkage are shown. **(b)** The final 3D density map. **(c)** The density map displays the overall conformation of the DNA origami Bennett linkage. A new conformation of DNA origami Bennett linkage was obtained (yellow ribbon) by flexible docking the Bennett linkage model into the density. **(d)** The FSC curve shows that the resolution of the final 3D reconstruction was ~13.0 nm. **(e–h)** The 3D density map of the 26th individual Bennett linkage was reconstructed from the tilt images using IPET. The FSC analysis showed that the 3D reconstruction resolution was ~11.7 nm. Scale bars are 20 nm.

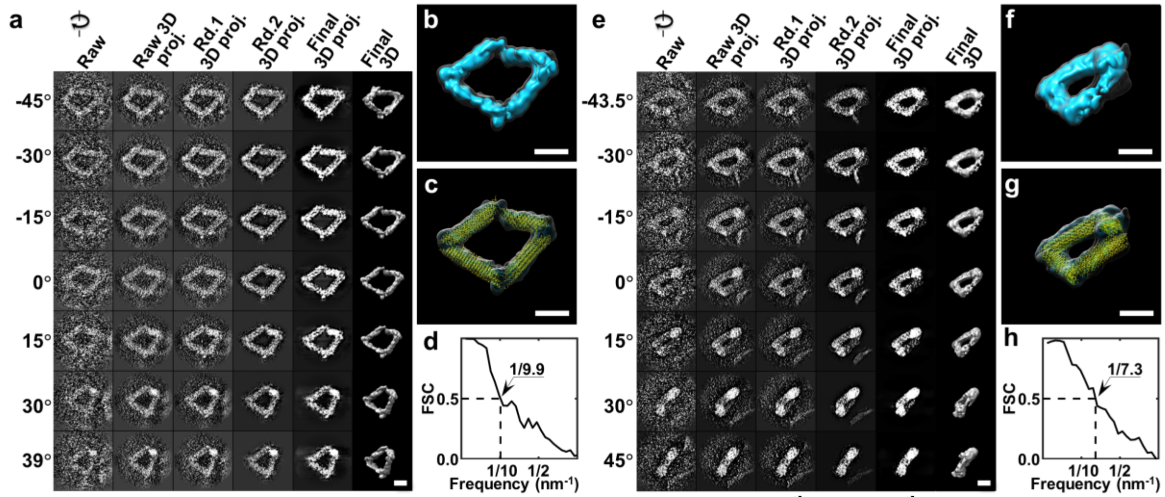

Supplementary Fig. 15 | IPET reconstruction processes on the 27th and 28th DNA origami Bennett linkage (a) Seven representative tilt views (first column), their corresponding projections on the intermediate 3D reconstructions from major iterations (second to fifth columns), and the 3D density map (sixth column) of the 27th particle of the DNA origami Bennett linkage are shown. **(b)** The final 3D density map. **(c)** The density map displays the overall conformation of the DNA origami Bennett linkage. A new conformation of DNA origami Bennett linkage was obtained (yellow ribbon) by flexible docking the Bennett linkage model into the density. **(d)** The FSC curve shows that the resolution of the final 3D reconstruction was ~9.9 nm. **(e–h)** The 3D density map of the 28th individual Bennett linkage was reconstructed from the tilt images using IPET. The FSC analysis showed that the 3D reconstruction resolution was ~7.3 nm. Scale bars are 20 nm.

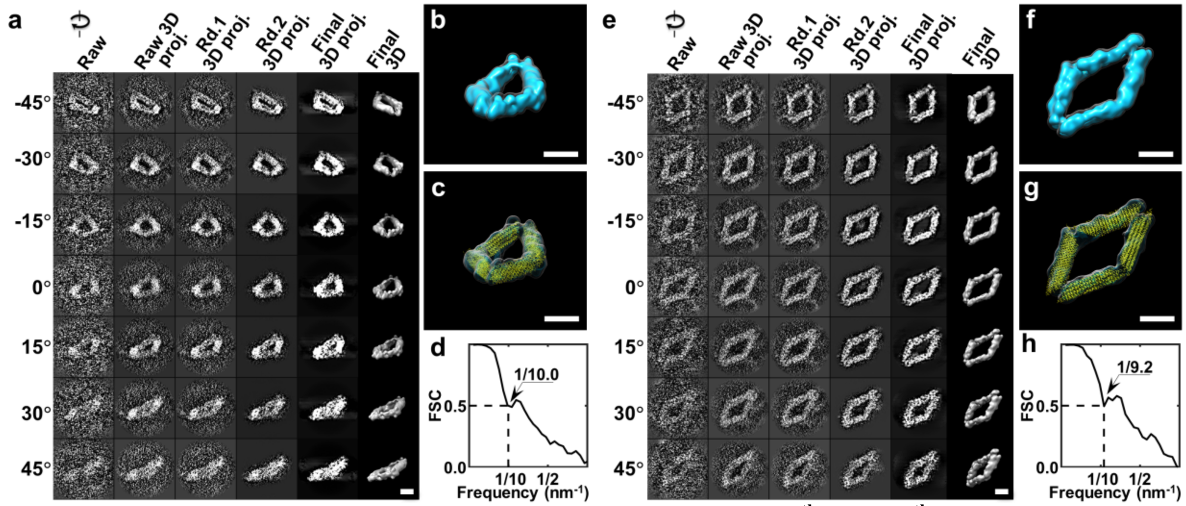

Supplementary Fig. 16 | IPET reconstruction processes on the 29th and 30th DNA origami Bennett linkage (a) Seven representative tilt views (first column), their corresponding projections on the intermediate 3D reconstructions from major iterations (second to fifth columns), and the 3D density map (sixth column) of the 29th particle of the DNA origami Bennett linkage are shown. **(b)** The final 3D density map. **(c)** The density map displays the overall conformation of the DNA origami Bennett linkage. A new conformation of DNA origami Bennett linkage was obtained (yellow ribbon) by flexible docking the Bennett linkage model into the density. **(d)** The FSC curve shows that the resolution of the final 3D reconstruction was ~10.0 nm. **(e–h)** The 3D density map of the 30th individual Bennett linkage was reconstructed from the tilt images using IPET. The FSC analysis showed that the 3D reconstruction resolution was ~9.2 nm. Scale bars are 20 nm.

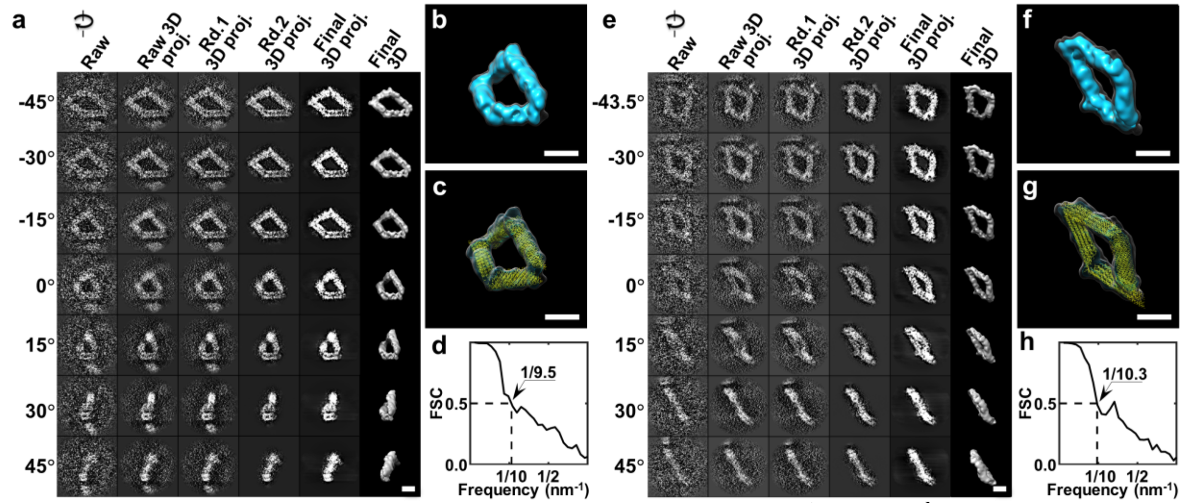

Supplementary Fig. 17 | IPET reconstruction processes on the 31st and 32nd DNA origami Bennett linkage (a) Seven representative tilt views (first column), their corresponding projections on the intermediate 3D reconstructions from major iterations (second to fifth columns), and the 3D density map (sixth column) of the 31st particle of the DNA origami Bennett linkage are shown. **(b)** The final 3D density map. **(c)** The density map displays the overall conformation of the DNA origami Bennett linkage. A new conformation of DNA origami Bennett linkage was obtained (yellow ribbon) by flexible docking the Bennett linkage model into the density. **(d)** The FSC curve shows that the resolution of the final 3D reconstruction was ~9.5 nm. **(e–h)** The 3D density map of the 32nd individual Bennett linkage was reconstructed from the tilt images using IPET. The FSC analysis showed that the 3D reconstruction resolution was ~10.3 nm. Scale bars are 20 nm.

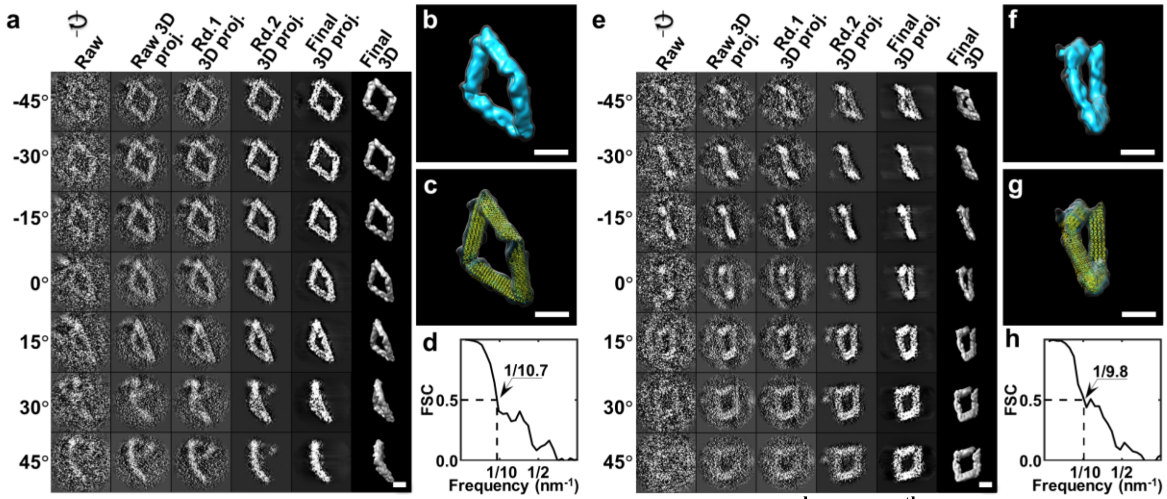

Supplementary Fig. 18 | IPET reconstruction processes on the 33rd and 34th DNA origami Bennett linkage (a) Seven representative tilt views (first column), their corresponding projections on the intermediate 3D reconstructions from major iterations (second to fifth columns), and the 3D density map (sixth column) of the 33rd particle of the DNA origami Bennett linkage are shown. **(b)** The final 3D density map. **(c)** The density map displays the overall conformation of the DNA origami Bennett linkage. A new conformation of DNA origami Bennett linkage was obtained (yellow ribbon) by flexible docking the Bennett linkage model into the density. **(d)** The FSC curve shows that the resolution of the final 3D reconstruction was ~10.7 nm. **(e–h)** The 3D density map of the 34th individual Bennett linkage was reconstructed from the tilt images using IPET. The FSC analysis showed that the 3D reconstruction resolution was ~9.8 nm. Scale bars are 20 nm.

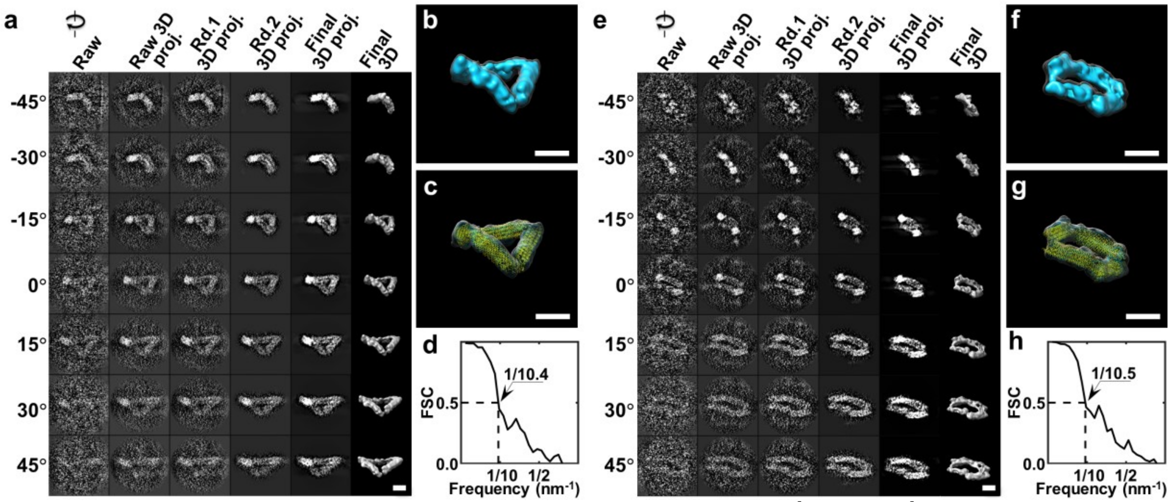

Supplementary Fig. 19 | IPET reconstruction processes on the 35th and 36th DNA origami Bennett linkage (a) Seven representative tilt views (first column), their corresponding projections on the intermediate 3D reconstructions from major iterations (second to fifth columns), and the 3D density map (sixth column) of the 35th particle of the DNA origami Bennett linkage are shown. **(b)** The final 3D density map. **(c)** The density map displays the overall conformation of the DNA origami Bennett linkage. A new conformation of DNA origami Bennett linkage was obtained (yellow ribbon) by flexible docking the Bennett linkage model into the density. **(d)** The FSC curve shows that the resolution of the final 3D reconstruction was ~10.4 nm. **(e–h)** The 3D density map of the 36th individual Bennett linkage was reconstructed from the tilt images using IPET. The FSC analysis showed that the 3D reconstruction resolution was ~10.5 nm. Scale bars are 20 nm.

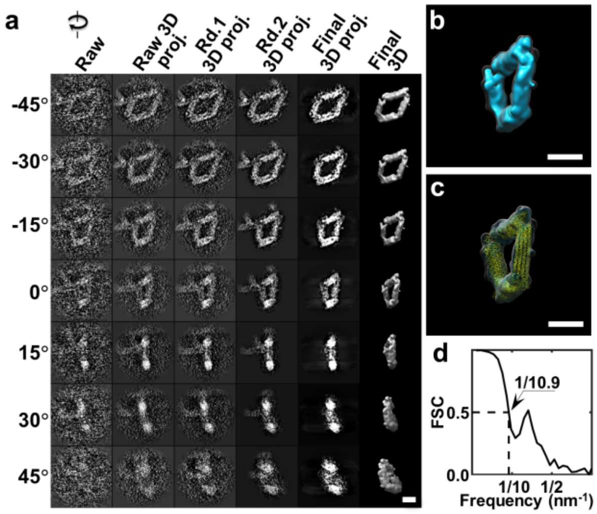

Supplementary Fig. 20 | IPET reconstruction processes on the 37th and 38th DNA origami Bennett linkage (a) Seven representative tilt views (first column), their corresponding projections on the intermediate 3D reconstructions from major iterations (second to fifth columns), and the 3D density map (sixth column) of the 37th particle of the DNA origami Bennett linkage are shown. **(b)** The final 3D density map. **(c)** The density map displays the overall conformation of the DNA origami Bennett linkage. A new conformation of DNA origami Bennett linkage was obtained (yellow ribbon) by flexible docking the Bennett linkage model into the density. **(d)** The FSC curve shows that the resolution of the final 3D reconstruction was ~10.9 nm. **(e–h)** The 3D density map of the 38th individual Bennett linkage was reconstructed from the tilt images using IPET. The FSC analysis showed that the 3D reconstruction resolution was ~10.3 nm. Scale bars are 20 nm.

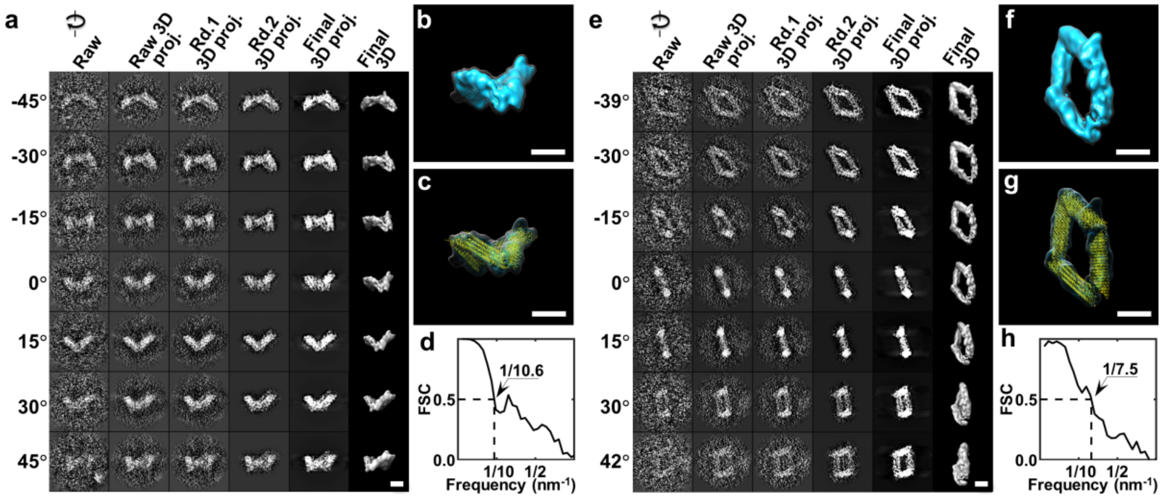

Supplementary Fig. 21 | IPET reconstruction processes on the 39th and 40th DNA origami Bennett linkage (a) Seven representative tilt views (first column), their corresponding projections on the intermediate 3D reconstructions from major iterations (second to fifth columns), and the 3D density map (sixth column) of the 39th particle of the DNA origami Bennett linkage are shown. **(b)** The final 3D density map. **(c)** The density map displays the overall conformation of the DNA origami Bennett linkage. A new conformation of DNA origami Bennett linkage was obtained (yellow ribbon) by flexible docking the Bennett linkage model into the density. **(d)** The FSC curve shows that the resolution of the final 3D reconstruction was ~10.6 nm. **(e–h)** The 3D density map of the 40th individual Bennett linkage was reconstructed from the tilt images using IPET. The FSC analysis showed that the 3D reconstruction resolution was ~7.5 nm. Scale bars are 20 nm.

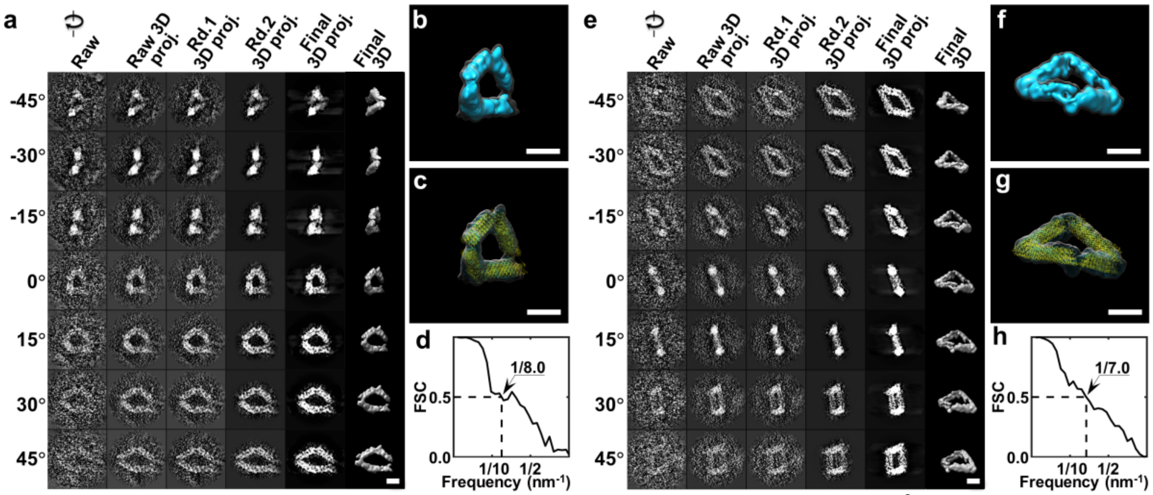

Supplementary Fig. 22 | IPET reconstruction processes on the 41st and 42nd DNA origami Bennett linkage (a) Seven representative tilt views (first column), their corresponding projections on the intermediate 3D reconstructions from major iterations (second to fifth columns), and the 3D density map (sixth column) of the 41st particle of the DNA origami Bennett linkage are shown. **(b)** The final 3D density map. **(c)** The density map displays the overall conformation of the DNA origami Bennett linkage. A new conformation of DNA origami Bennett linkage was obtained (yellow ribbon) by flexible docking the Bennett linkage model into the density. **(d)** The FSC curve shows that the resolution of the final 3D reconstruction was ~8.0 nm. **(e–h)** The 3D density map of the 42nd individual Bennett linkage was reconstructed from the tilt images using IPET. The FSC analysis showed that the 3D reconstruction resolution was ~7.0 nm. Scale bars are 20 nm.

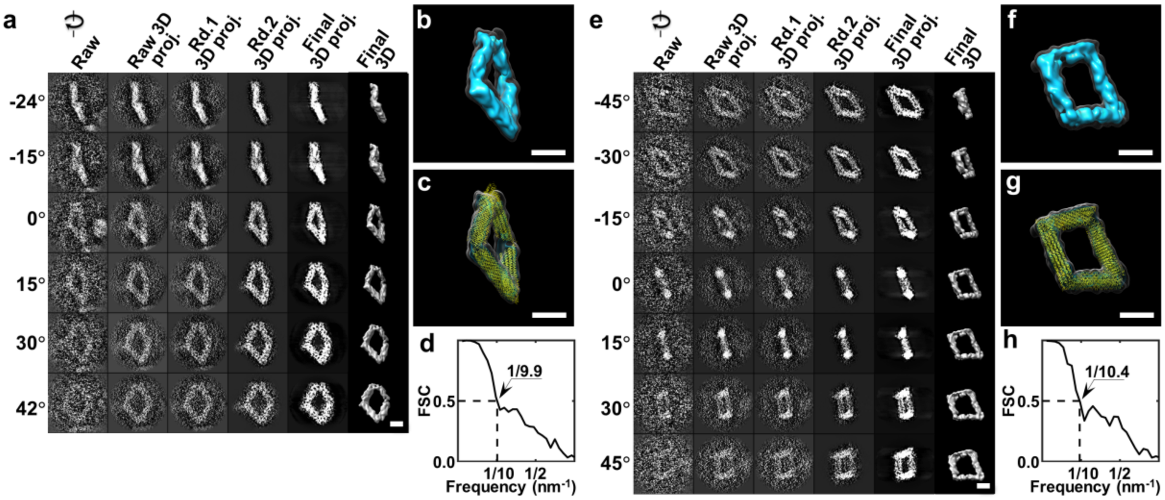

Supplementary Fig. 23 | IPET reconstruction processes on the 43rd and 44th DNA origami Bennett linkage (a) Seven representative tilt views (first column), their corresponding projections on the intermediate 3D reconstructions from major iterations (second to fifth columns), and the 3D density map (sixth column) of the 43rd particle of the DNA origami Bennett linkage are shown. **(b)** The final 3D density map. **(c)** The density map displays the overall conformation of the DNA origami Bennett linkage. A new conformation of DNA origami Bennett linkage was obtained (yellow ribbon) by flexible docking the Bennett linkage model into the density. **(d)** The FSC curve shows that the resolution of the final 3D reconstruction was ~9.9 nm. **(e–h)** The 3D density map of the 44th individual Bennett linkage was reconstructed from the tilt images using IPET. The FSC analysis showed that the 3D reconstruction resolution was ~10.4 nm. Scale bars are 20 nm.

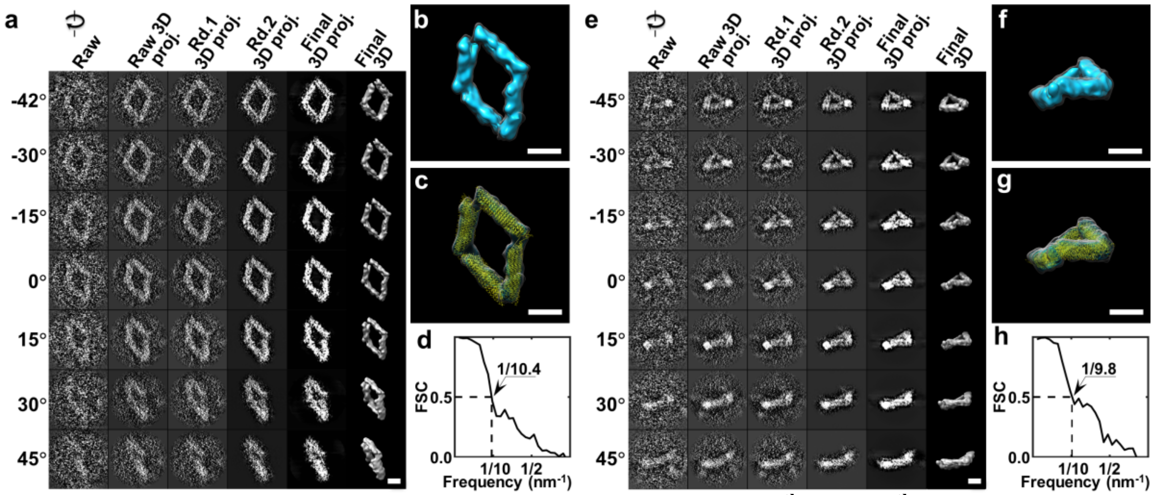

Supplementary Fig. 24 | IPET reconstruction processes on the 45th and 46th DNA origami Bennett linkage (a) Seven representative tilt views (first column), their corresponding projections on the intermediate 3D reconstructions from major iterations (second to fifth columns), and the 3D density map (sixth column) of the 45th particle of the DNA origami Bennett linkage are shown. **(b)** The final 3D density map. **(c)** The density map displays the overall conformation of the DNA origami Bennett linkage. A new conformation of DNA origami Bennett linkage was obtained (yellow ribbon) by flexible docking the Bennett linkage model into the density. **(d)** The FSC curve shows that the resolution of the final 3D reconstruction was ~10.4 nm. **(e–h)** The 3D density map of the 46th individual Bennett linkage was reconstructed from the tilt images using IPET. The FSC analysis showed that the 3D reconstruction resolution was ~9.8 nm. Scale bars are 20 nm.

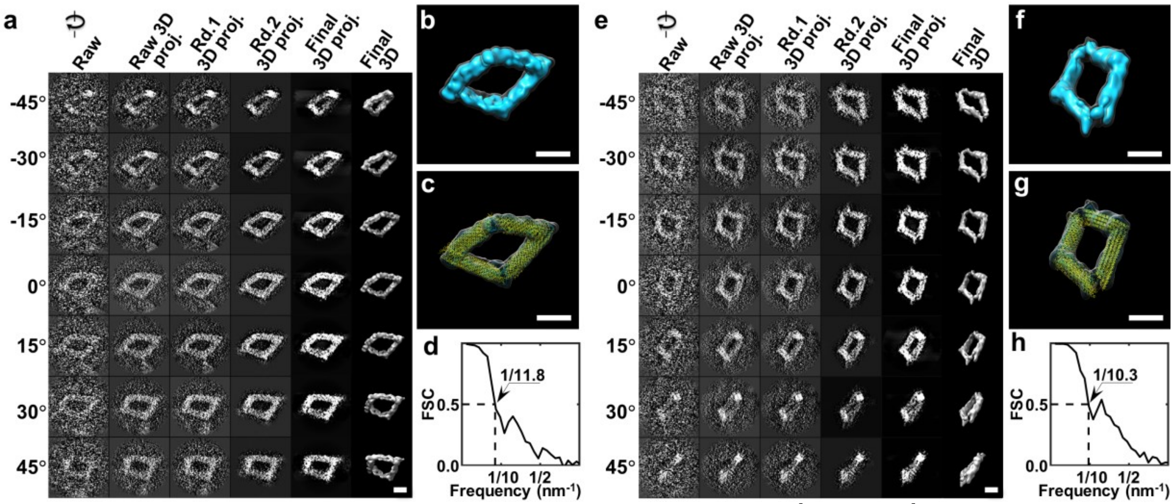

Supplementary Fig. 25 | IPET reconstruction processes on the 47th and 48th DNA origami Bennett linkage (a) Seven representative tilt views (first column), their corresponding projections on the intermediate 3D reconstructions from major iterations (second to fifth columns), and the 3D density map (sixth column) of the 47th particle of the DNA origami Bennett linkage are shown. **(b)** The final 3D density map. **(c)** The density map displays the overall conformation of the DNA origami Bennett linkage. A new conformation of DNA origami Bennett linkage was obtained (yellow ribbon) by flexible docking the Bennett linkage model into the density. **(d)** The FSC curve shows that the resolution of the final 3D reconstruction was ~11.8 nm. **(e–h)** The 3D density map of the 48th individual Bennett linkage was reconstructed from the tilt images using IPET. The FSC analysis showed that the 3D reconstruction resolution was ~10.3 nm. Scale bars are 20 nm.

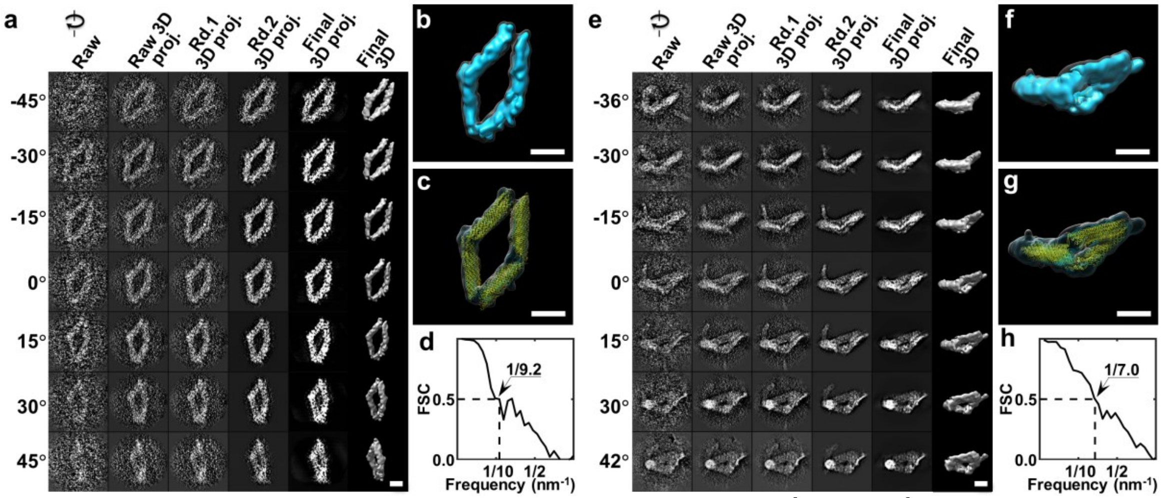

Supplementary Fig. 26 | IPET reconstruction processes on the 49th and 50th DNA origami Bennett linkage (a) Seven representative tilt views (first column), their corresponding projections on the intermediate 3D reconstructions from major iterations (second to fifth columns), and the 3D density map (sixth column) of the 49th particle of the DNA origami Bennett linkage are shown. **(b)** The final 3D density map. **(c)** The density map displays the overall conformation of the DNA origami Bennett linkage. A new conformation of DNA origami Bennett linkage was obtained (yellow ribbon) by flexible docking the Bennett linkage model into the density. **(d)** The FSC curve shows that the resolution of the final 3D reconstruction was ~9.2nm. **(e–h)** The 3D density map of the 50th individual Bennett linkage was reconstructed from the tilt images using IPET. The FSC analysis showed that the 3D reconstruction resolution was ~7.0 nm. Scale bars are 20 nm.

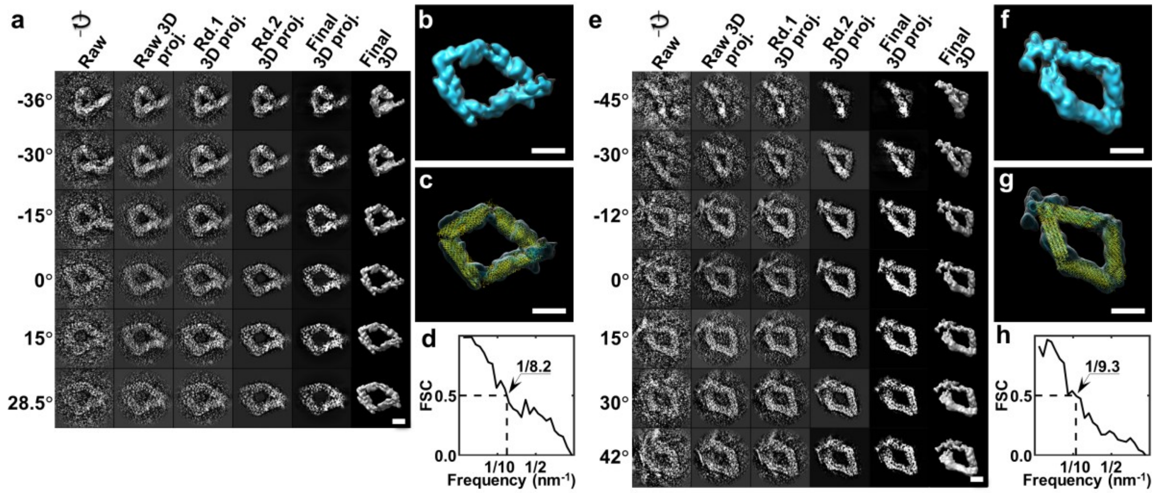

Supplementary Fig. 27 | IPET reconstruction processes on the 51st and 52nd DNA origami Bennett linkage (a) Seven representative tilt views (first column), their corresponding projections on the intermediate 3D reconstructions from major iterations (second to fifth columns), and the 3D density map (sixth column) of the 51st particle of the DNA origami Bennett linkage are shown. **(b)** The final 3D density map. **(c)** The density map displays the overall conformation of the DNA origami Bennett linkage. A new conformation of DNA origami Bennett linkage was obtained (yellow ribbon) by flexible docking the Bennett linkage model into the density. **(d)** The FSC curve shows that the resolution of the final 3D reconstruction was ~8.2 nm. **(e–h)** The 3D density map of the 52nd individual Bennett linkage was reconstructed from the tilt images using IPET. The FSC analysis showed that the 3D reconstruction resolution was ~9.3 nm. Scale bars are 20 nm.

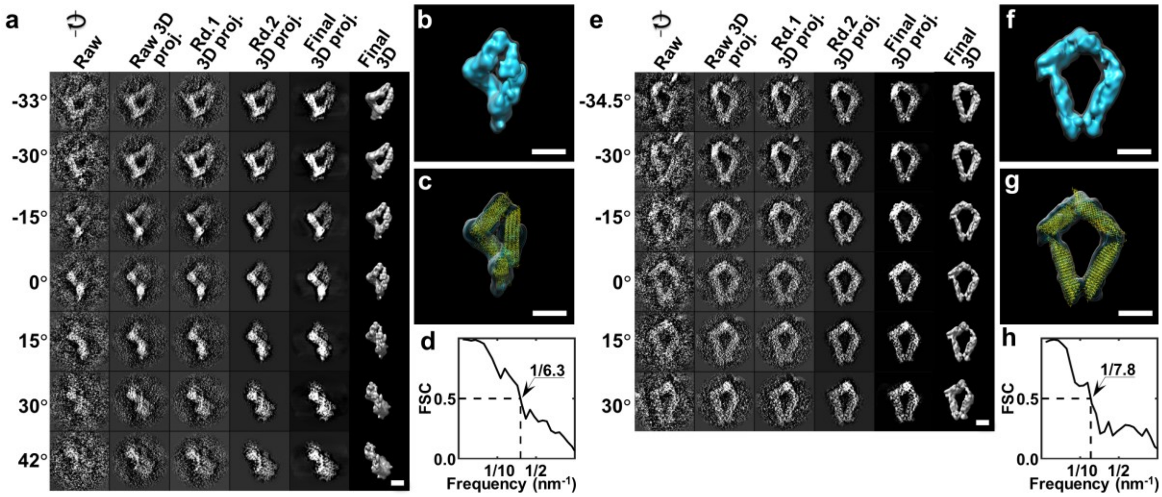

Supplementary Fig. 28 | IPET reconstruction processes on the 53rd and 54th DNA origami Bennett linkage (a) Seven representative tilt views (first column), their corresponding projections on the intermediate 3D reconstructions from major iterations (second to fifth columns), and the 3D density map (sixth column) of the 53rd particle of the DNA origami Bennett linkage are shown. **(b)** The final 3D density map. **(c)** The density map displays the overall conformation of the DNA origami Bennett linkage. A new conformation of DNA origami Bennett linkage was obtained (yellow ribbon) by flexible docking the Bennett linkage model into the density. **(d)** The FSC curve shows that the resolution of the final 3D reconstruction was ~6.3 nm. **(e–h)** The 3D density map of the 54th individual Bennett linkage was reconstructed from the tilt images using IPET. The FSC analysis showed that the 3D reconstruction resolution was ~7.8 nm. Scale bars are 20 nm.

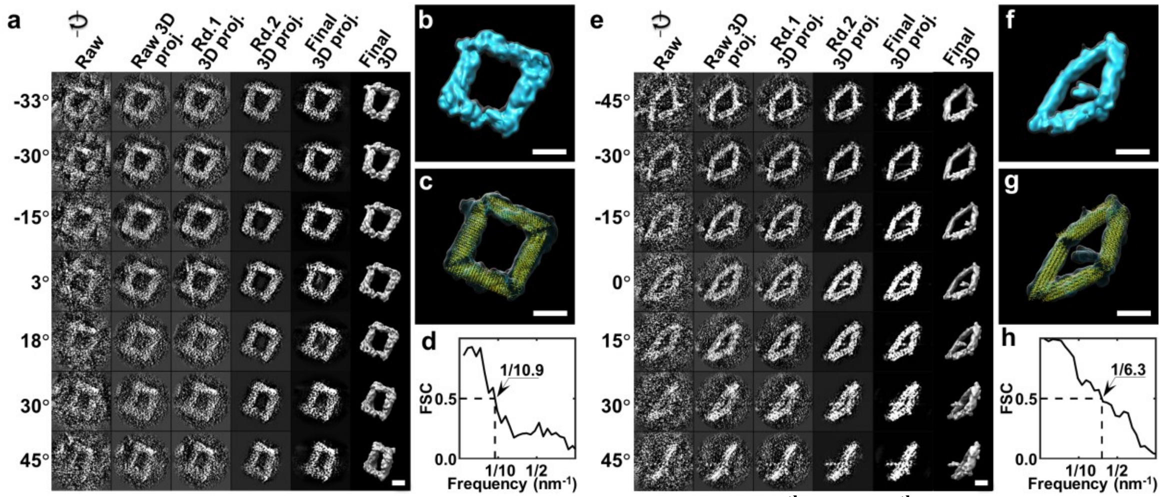

Supplementary Fig. 29 | IPET reconstruction processes on the 55th and 56th DNA origami Bennett linkage (a) Seven representative tilt views (first column), their corresponding projections on the intermediate 3D reconstructions from major iterations (second to fifth columns), and the 3D density map (sixth column) of the 55th particle of the DNA origami Bennett linkage are shown. **(b)** The final 3D density map. **(c)** The density map displays the overall conformation of the DNA origami Bennett linkage. A new conformation of DNA origami Bennett linkage was obtained (yellow ribbon) by flexible docking the Bennett linkage model into the density. **(d)** The FSC curve shows that the resolution of the final 3D reconstruction was ~10.9 nm. **(e–h)** The 3D density map of the 56th individual Bennett linkage was reconstructed from the tilt images using IPET. The FSC analysis showed that the 3D reconstruction resolution was ~6.3 nm. Scale bars are 20 nm.

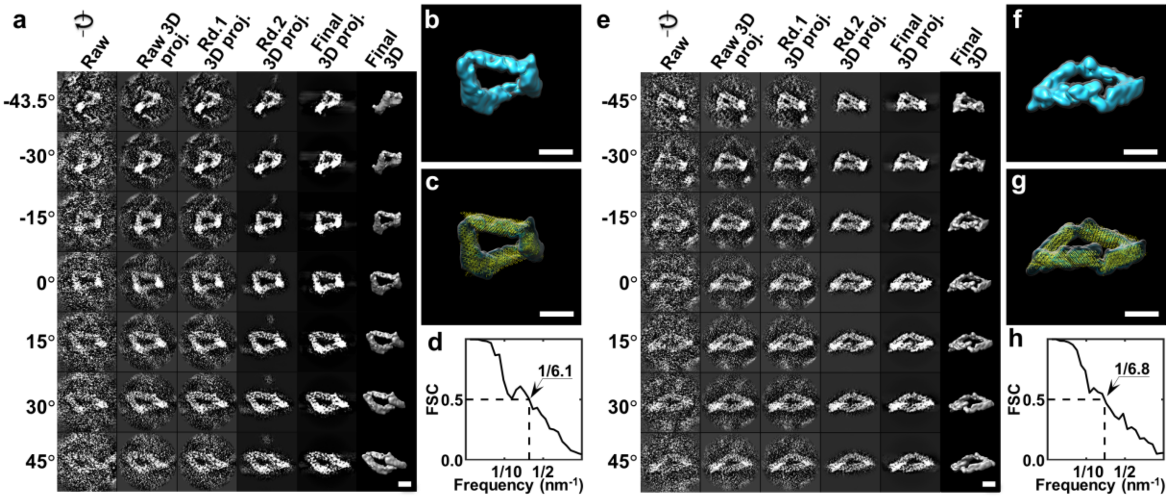

Supplementary Fig. 30 | IPET reconstruction processes on the 57th and 58th DNA origami Bennett linkage (a) Seven representative tilt views (first column), their corresponding projections on the intermediate 3D reconstructions from major iterations (second to fifth columns), and the 3D density map (sixth column) of the 57th particle of the DNA origami Bennett linkage are shown. **(b)** The final 3D density map. **(c)** The density map displays the overall conformation of the DNA origami Bennett linkage. A new conformation of DNA origami Bennett linkage was obtained (yellow ribbon) by flexible docking the Bennett linkage model into the density. **(d)** The FSC curve shows that the resolution of the final 3D reconstruction was ~6.1 nm. **(e–h)** The 3D density map of the 58th individual Bennett linkage was reconstructed from the tilt images using IPET. The FSC analysis showed that the 3D reconstruction resolution was ~6.8 nm. Scale bars are 20 nm.

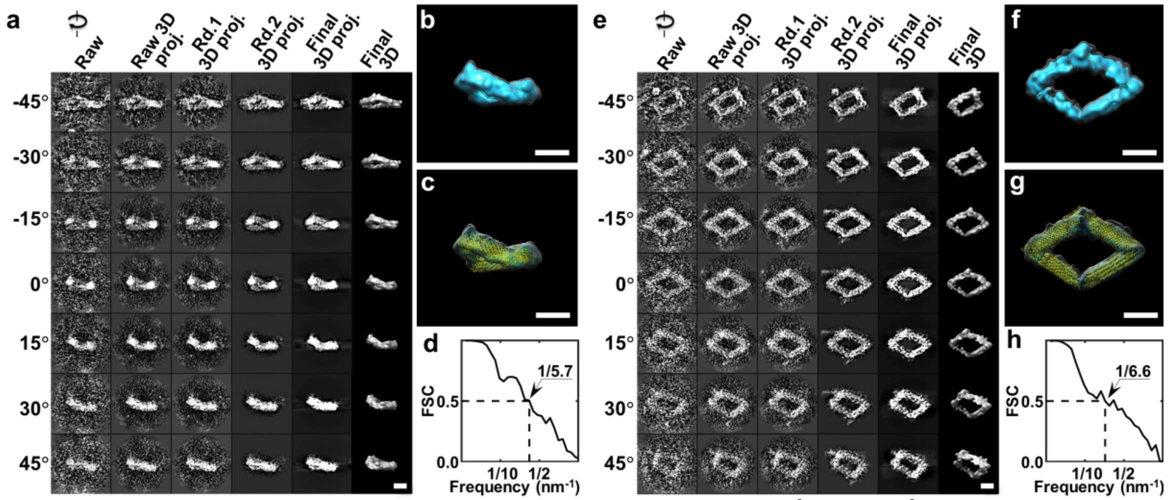

Supplementary Fig. 31 | IPET reconstruction processes on the 59th and 60th DNA origami Bennett linkage (a) Seven representative tilt views (first column), their corresponding projections on the intermediate 3D reconstructions from major iterations (second to fifth columns), and the 3D density map (sixth column) of the 59th particle of the DNA origami Bennett linkage are shown. **(b)** The final 3D density map. **(c)** The density map displays the overall conformation of the DNA origami Bennett linkage. A new conformation of DNA origami Bennett linkage was obtained (yellow ribbon) by flexible docking the Bennett linkage model into the density. **(d)** The FSC curve shows that the resolution of the final 3D reconstruction was ~5.7 nm. **(e–h)** The 3D density map of the 60th individual Bennett linkage was reconstructed from the tilt images using IPET. The FSC analysis showed that the 3D reconstruction resolution was ~6.6 nm. Scale bars are 20 nm.

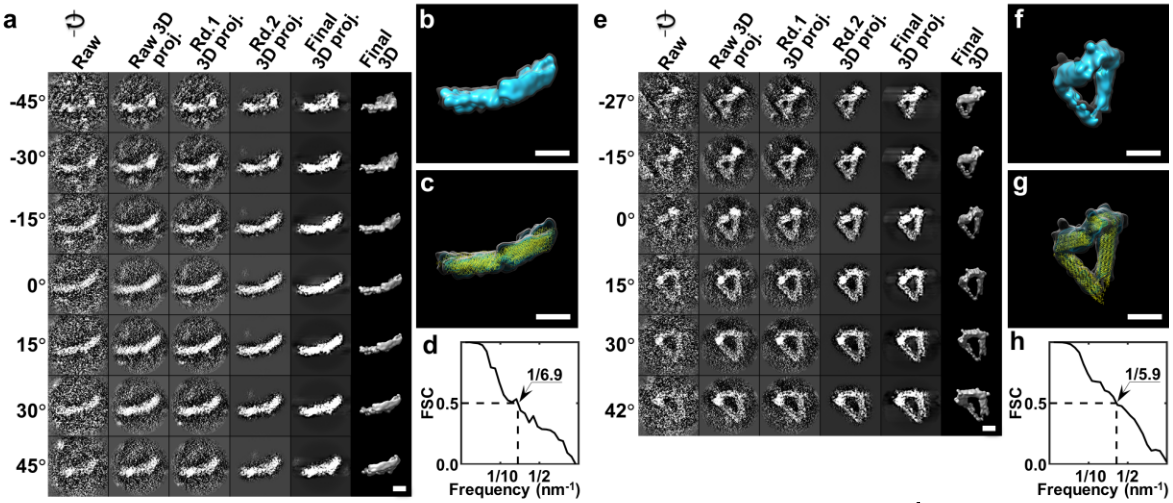

Supplementary Fig. 32 | IPET reconstruction processes on the 61st and 62nd DNA origami Bennett linkage (a) Seven representative tilt views (first column), their corresponding projections on the intermediate 3D reconstructions from major iterations (second to fifth columns), and the 3D density map (sixth column) of the 61st particle of the DNA origami Bennett linkage are shown. **(b)** The final 3D density map. **(c)** The density map displays the overall conformation of the DNA origami Bennett linkage. A new conformation of DNA origami Bennett linkage was obtained (yellow ribbon) by flexible docking the Bennett linkage model into the density. **(d)** The FSC curve shows that the resolution of the final 3D reconstruction was ~6.9 nm. **(e–h)** The 3D density map of the 62nd individual Bennett linkage was reconstructed from the tilt images using IPET. The FSC analysis showed that the 3D reconstruction resolution was ~5.9 nm. Scale bars are 20 nm.

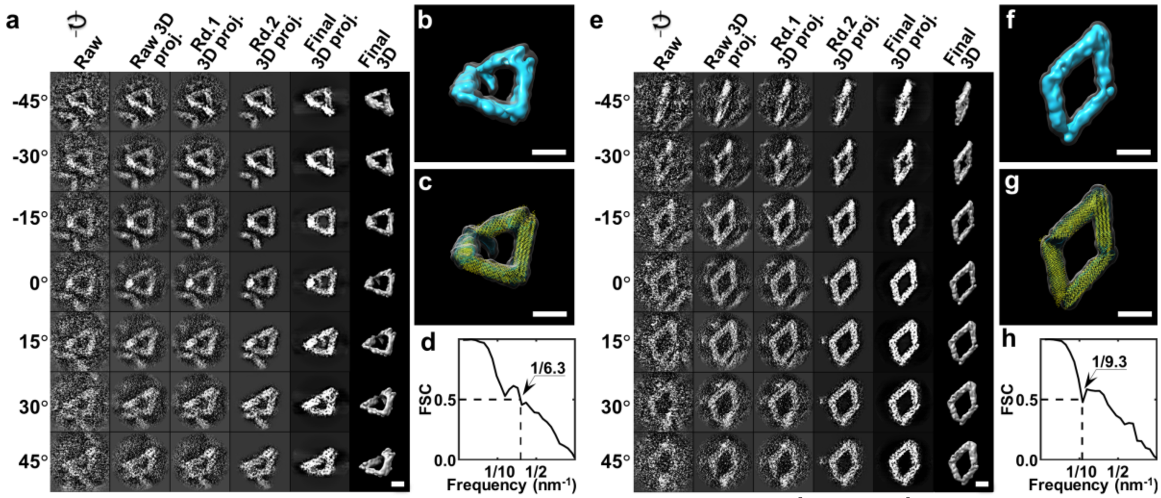

Supplementary Fig. 33 | IPET reconstruction processes on the 63rd and 64th DNA origami Bennett linkage (a) Seven representative tilt views (first column), their corresponding projections on the intermediate 3D reconstructions from major iterations (second to fifth columns), and the 3D density map (sixth column) of the 63rd particle of the DNA origami Bennett linkage are shown. **(b)** The final 3D density map. **(c)** The density map displays the overall conformation of the DNA origami Bennett linkage. A new conformation of DNA origami Bennett linkage was obtained (yellow ribbon) by flexible docking the Bennett linkage model into the density. **(d)** The FSC curve shows that the resolution of the final 3D reconstruction was ~6.3 nm. **(e–h)** The 3D density map of the 64th individual Bennett linkage was reconstructed from the tilt images using IPET. The FSC analysis showed that the 3D reconstruction resolution was ~9.3 nm. Scale bars are 20 nm.

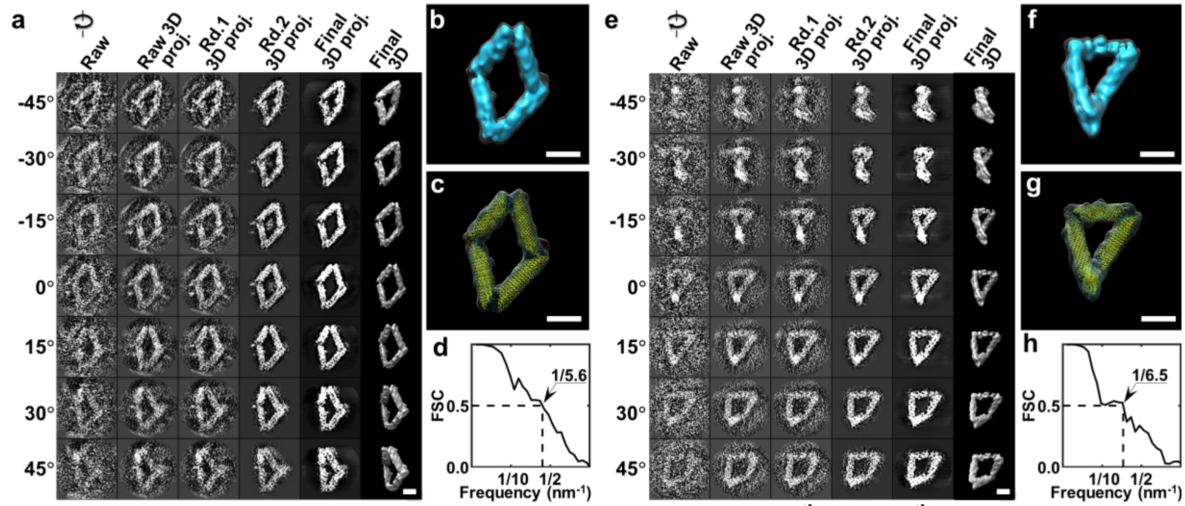

Supplementary Fig. 34 | IPET reconstruction processes on the 65th and 66th DNA origami Bennett linkage (a) Seven representative tilt views (first column), their corresponding projections on the intermediate 3D reconstructions from major iterations (second to fifth columns), and the 3D density map (sixth column) of the 65th particle of the DNA origami Bennett linkage are shown. **(b)** The final 3D density map. **(c)** The density map displays the overall conformation of the DNA origami Bennett linkage. A new conformation of DNA origami Bennett linkage was obtained (yellow ribbon) by flexible docking the Bennett linkage model into the density. **(d)** The FSC curve shows that the resolution of the final 3D reconstruction was ~5.6 nm. **(e–h)** The 3D density map of the 66th individual Bennett linkage was reconstructed from the tilt images using IPET. The FSC analysis showed that the 3D reconstruction resolution was ~6.5 nm. Scale bars are 20 nm.

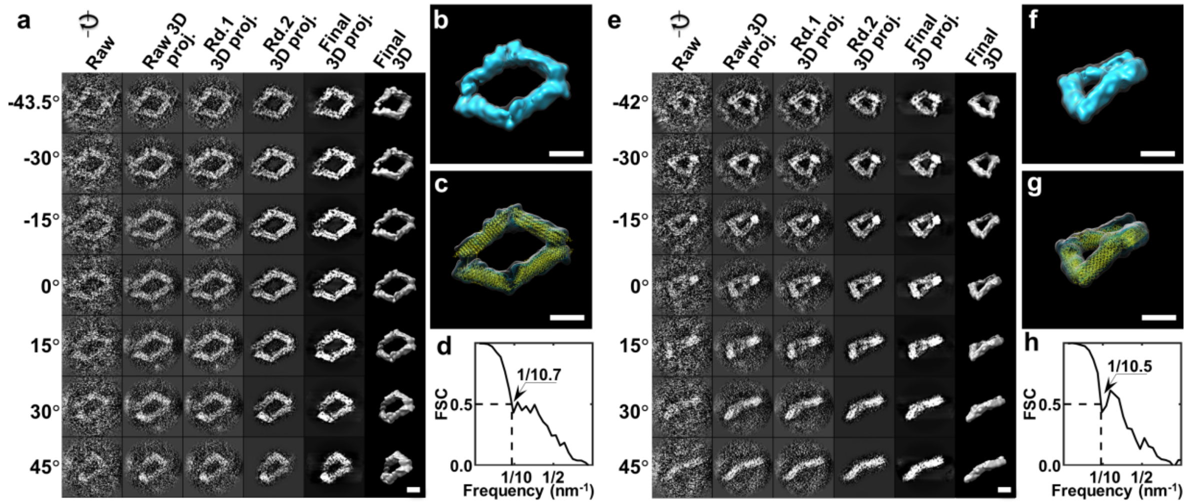

Supplementary Fig. 35 | IPET reconstruction processes on the 67th and 68th DNA origami Bennett linkage (a) Seven representative tilt views (first column), their corresponding projections on the intermediate 3D reconstructions from major iterations (second to fifth columns), and the 3D density map (sixth column) of the 67th particle of the DNA origami Bennett linkage are shown. **(b)** The final 3D density map. **(c)** The density map displays the overall conformation of the DNA origami Bennett linkage. A new conformation of DNA origami Bennett linkage was obtained (yellow ribbon) by flexible docking the Bennett linkage model into the density. **(d)** The FSC curve shows that the resolution of the final 3D reconstruction was ~10.7 nm. **(e–h)** The 3D density map of the 68th individual Bennett linkage was reconstructed from the tilt images using IPET. The FSC analysis showed that the 3D reconstruction resolution was ~10.5 nm. Scale bars are 20 nm.

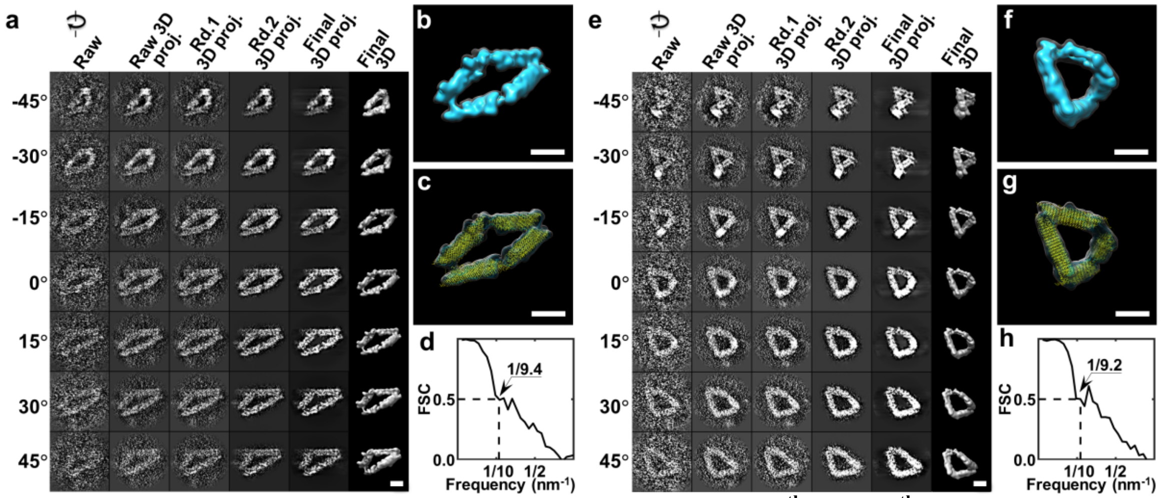

Supplementary Fig. 36 | IPET reconstruction processes on the 69th and 70th DNA origami Bennett linkage (a) Seven representative tilt views (first column), their corresponding projections on the intermediate 3D reconstructions from major iterations (second to fifth columns), and the 3D density map (sixth column) of the 69th particle of the DNA origami Bennett linkage are shown. **(b)** The final 3D density map. **(c)** The density map displays the overall conformation of the DNA origami Bennett linkage. A new conformation of DNA origami Bennett linkage was obtained (yellow ribbon) by flexible docking the Bennett linkage model into the density. **(d)** The FSC curve shows that the resolution of the final 3D reconstruction was ~9.4 nm. **(e–h)** The 3D density map of the 70th individual Bennett linkage was reconstructed from the tilt images using IPET. The FSC analysis showed that the 3D reconstruction resolution was ~9.2 nm. Scale bars are 20 nm.

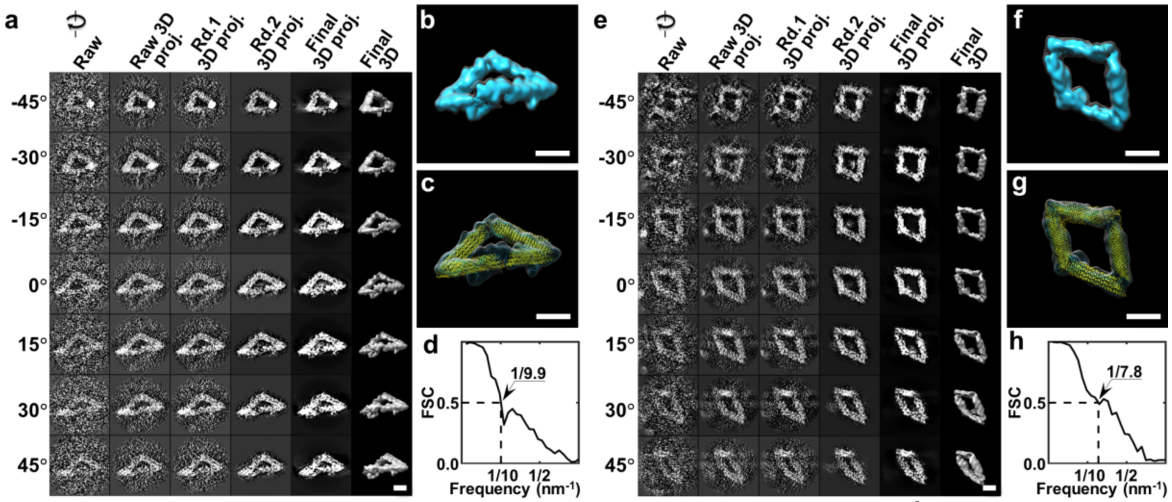

Supplementary Fig. 37 | IPET reconstruction processes on the 71st and 72nd DNA origami Bennett linkage (a) Seven representative tilt views (first column), their corresponding projections on the intermediate 3D reconstructions from major iterations (second to fifth columns), and the 3D density map (sixth column) of the 71st particle of the DNA origami Bennett linkage are shown. **(b)** The final 3D density map. **(c)** The density map displays the overall conformation of the DNA origami Bennett linkage. A new conformation of DNA origami Bennett linkage was obtained (yellow ribbon) by flexible docking the Bennett linkage model into the density. **(d)** The FSC curve shows that the resolution of the final 3D reconstruction was ~9.9 nm. **(e–h)** The 3D density map of the 72nd individual Bennett linkage was reconstructed from the tilt images using IPET. The FSC analysis showed that the 3D reconstruction resolution was ~7.8 nm. Scale bars are 20 nm.

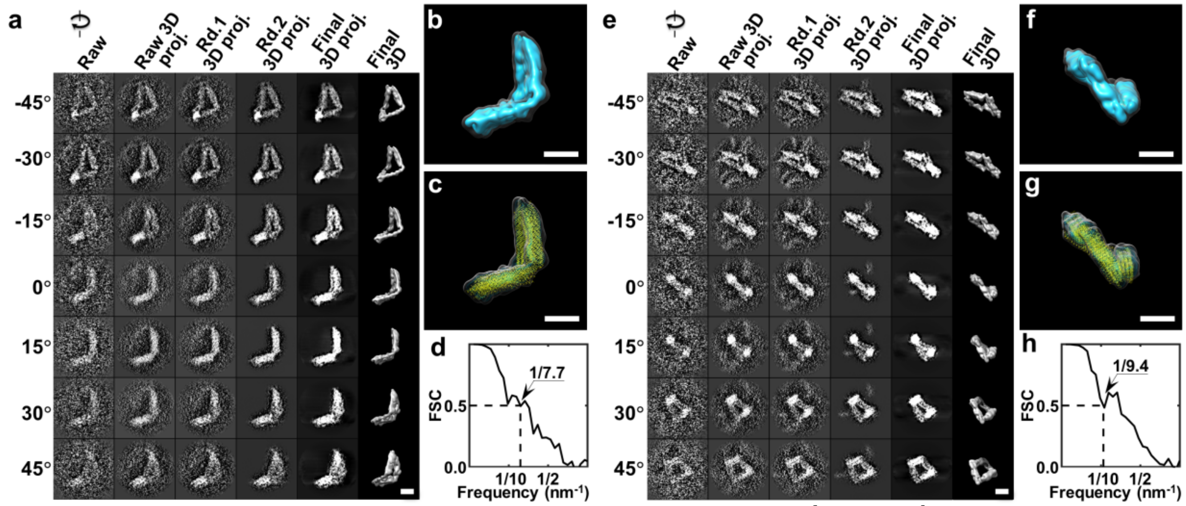

Supplementary Fig. 38 | IPET reconstruction processes on the 73rd and 74th DNA origami Bennett linkage (a) Seven representative tilt views (first column), their corresponding projections on the intermediate 3D reconstructions from major iterations (second to fifth columns), and the 3D density map (sixth column) of the 73rd particle of the DNA origami Bennett linkage are shown. **(b)** The final 3D density map. **(c)** The density map displays the overall conformation of the DNA origami Bennett linkage. A new conformation of DNA origami Bennett linkage was obtained (yellow ribbon) by flexible docking the Bennett linkage model into the density. **(d)** The FSC curve shows that the resolution of the final 3D reconstruction was ~7.7 nm. **(e–h)** The 3D density map of the 74th individual Bennett linkage was reconstructed from the tilt images using IPET. The FSC analysis showed that the 3D reconstruction resolution was ~9.4 nm. Scale bars are 20 nm.

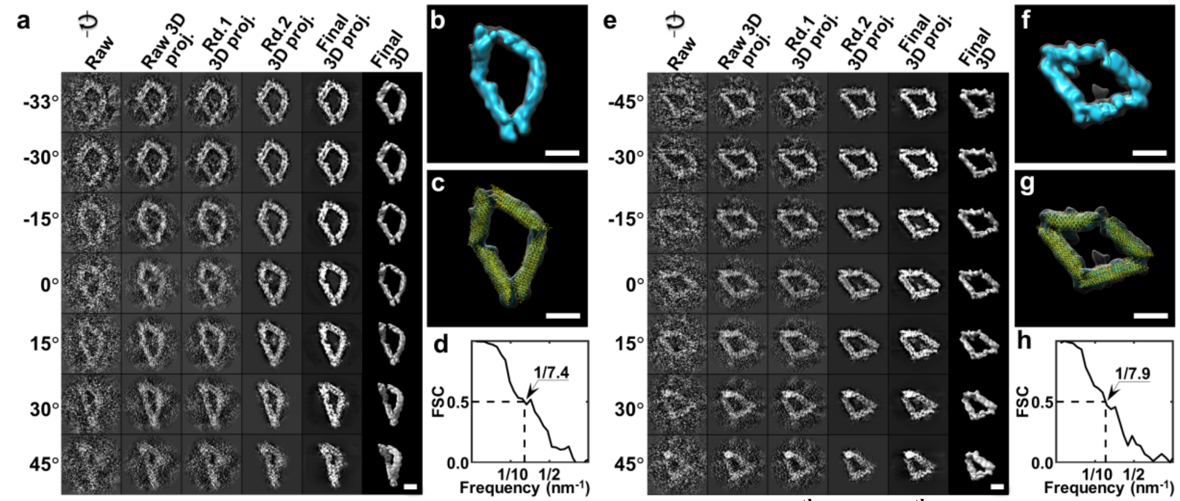

Supplementary Fig. 39 | IPET reconstruction processes on the 75th and 76th DNA origami Bennett linkage (a) Seven representative tilt views (first column), their corresponding projections on the intermediate 3D reconstructions from major iterations (second to fifth columns), and the 3D density map (sixth column) of the 75th particle of the DNA origami Bennett linkage are shown. **(b)** The final 3D density map. **(c)** The density map displays the overall conformation of the DNA origami Bennett linkage. A new conformation of DNA origami Bennett linkage was obtained (yellow ribbon) by flexible docking the Bennett linkage model into the density. **(d)** The FSC curve shows that the resolution of the final 3D reconstruction was ~7.4 nm. **(e–h)** The 3D density map of the 76th individual Bennett linkage was reconstructed from the tilt images using IPET. The FSC analysis showed that the 3D reconstruction resolution was ~7.9 nm. Scale bars are 20 nm.

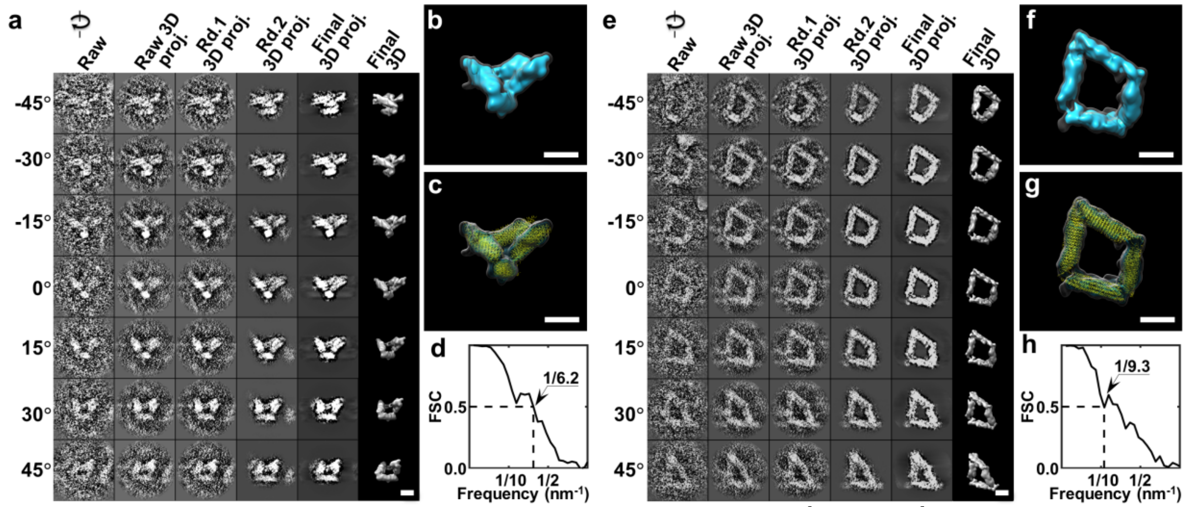

Supplementary Fig. 40 | IPET reconstruction processes on the 77th and 78th DNA origami Bennett linkage (a) Seven representative tilt views (first column), their corresponding projections on the intermediate 3D reconstructions from major iterations (second to fifth columns), and the 3D density map (sixth column) of the 77th particle of the DNA origami Bennett linkage are shown. **(b)** The final 3D density map. **(c)** The density map displays the overall conformation of the DNA origami Bennett linkage. A new conformation of DNA origami Bennett linkage was obtained (yellow ribbon) by flexible docking the Bennett linkage model into the density. **(d)** The FSC curve shows that the resolution of the final 3D reconstruction was ~6.2 nm. **(e–h)** The 3D density map of the 78th individual Bennett linkage was reconstructed from the tilt images using IPET. The FSC analysis showed that the 3D reconstruction resolution was ~9.3 nm. Scale bars are 20 nm.

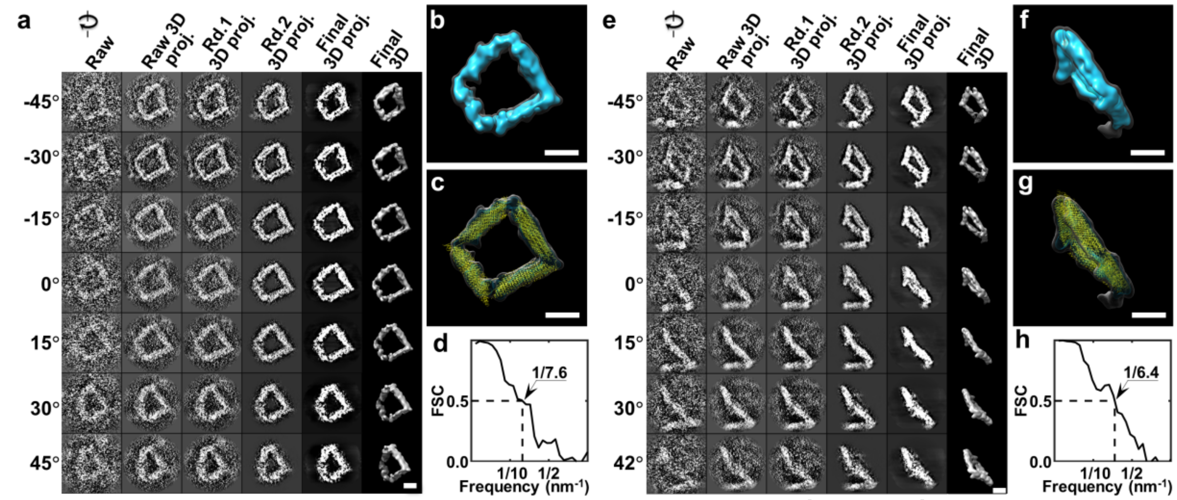

Supplementary Fig. 41 | IPET reconstruction processes on the 79th and 80th DNA origami Bennett linkage (a) Seven representative tilt views (first column), their corresponding projections on the intermediate 3D reconstructions from major iterations (second to fifth columns), and the 3D density map (sixth column) of the 79th particle of the DNA origami Bennett linkage are shown. **(b)** The final 3D density map. **(c)** The density map displays the overall conformation of the DNA origami Bennett linkage. A new conformation of DNA origami Bennett linkage was obtained (yellow ribbon) by flexible docking the Bennett linkage model into the density. **(d)** The FSC curve shows that the resolution of the final 3D reconstruction was ~7.6 nm. **(e–h)** The 3D density map of the 80th individual Bennett linkage was reconstructed from the tilt images using IPET. The FSC analysis showed that the 3D reconstruction resolution was ~6.4 nm. Scale bars are 20 nm.

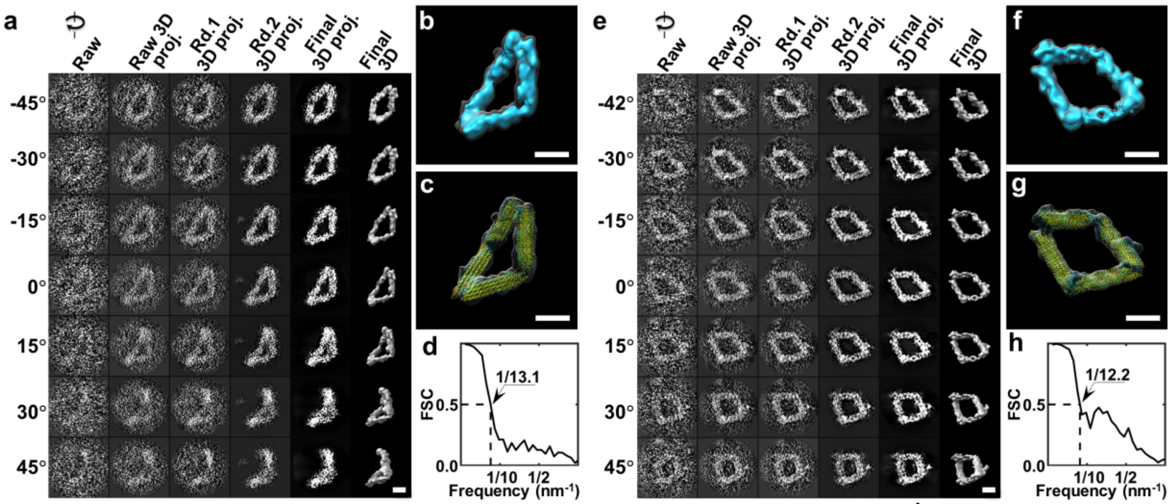

Supplementary Fig. 42 | IPET reconstruction processes on the 81st and 82nd DNA origami Bennett linkage (a) Seven representative tilt views (first column), their corresponding projections on the intermediate 3D reconstructions from major iterations (second to fifth columns), and the 3D density map (sixth column) of the 81st particle of the DNA origami Bennett linkage are shown. **(b)** The final 3D density map. **(c)** The density map displays the overall conformation of the DNA origami Bennett linkage. A new conformation of DNA origami Bennett linkage was obtained (yellow ribbon) by flexible docking the Bennett linkage model into the density. **(d)** The FSC curve shows that the resolution of the final 3D reconstruction was ~13.1 nm. **(e–h)** The 3D density map of the 82nd individual Bennett linkage was reconstructed from the tilt images using IPET. The FSC analysis showed that the 3D reconstruction resolution was ~12.2 nm. Scale bars are 20 nm.

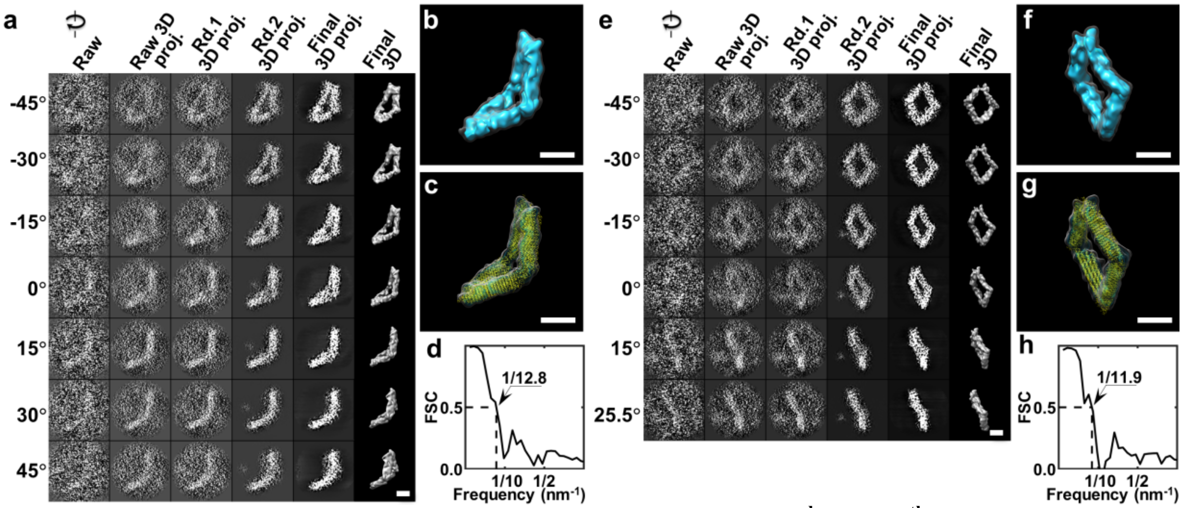

Supplementary Fig. 43 | IPET reconstruction processes on the 83rd and 84th DNA origami Bennett linkage (a) Seven representative tilt views (first column), their corresponding projections on the intermediate 3D reconstructions from major iterations (second to fifth columns), and the 3D density map (sixth column) of the 83rd particle of the DNA origami Bennett linkage are shown. **(b)** The final 3D density map. **(c)** The density map displays the overall conformation of the DNA origami Bennett linkage. A new conformation of DNA origami Bennett linkage was obtained (yellow ribbon) by flexible docking the Bennett linkage model into the density. **(d)** The FSC curve shows that the resolution of the final 3D reconstruction was ~12.8 nm. **(e–h)** The 3D density map of the 84th individual Bennett linkage was reconstructed from the tilt images using IPET. The FSC analysis showed that the 3D reconstruction resolution was ~11.9 nm. Scale bars are 20 nm.

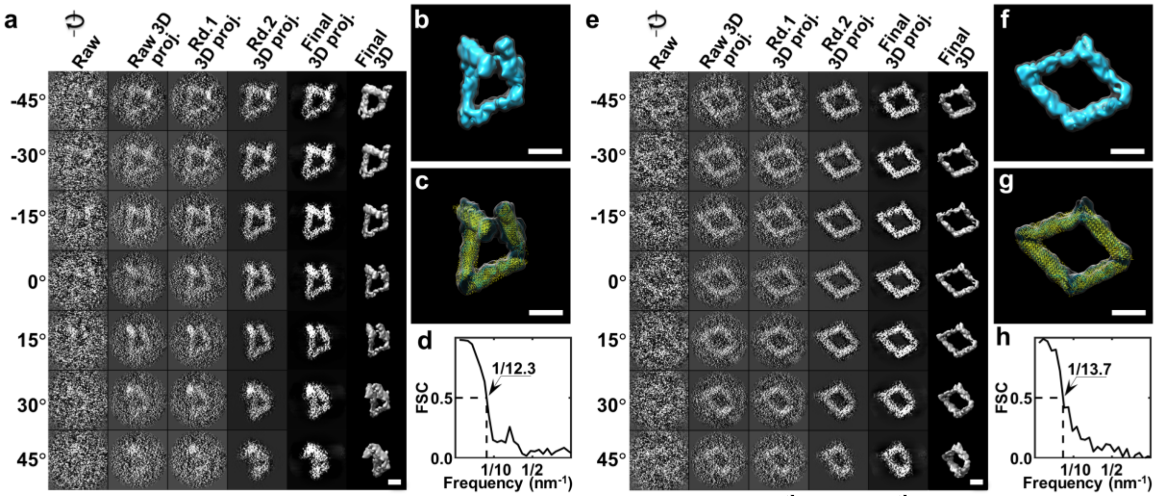

Supplementary Fig. 44 | IPET reconstruction processes on the 85th and 86th DNA origami Bennett linkage (a) Seven representative tilt views (first column), their corresponding projections on the intermediate 3D reconstructions from major iterations (second to fifth columns), and the 3D density map (sixth column) of the 85th particle of the DNA origami Bennett linkage are shown. **(b)** The final 3D density map. **(c)** The density map displays the overall conformation of the DNA origami Bennett linkage. A new conformation of DNA origami Bennett linkage was obtained (yellow ribbon) by flexible docking the Bennett linkage model into the density. **(d)** The FSC curve shows that the resolution of the final 3D reconstruction was ~12.3 nm. **(e–h)** The 3D density map of the 86th individual Bennett linkage was reconstructed from the tilt images using IPET. The FSC analysis showed that the 3D reconstruction resolution was ~13.7 nm. Scale bars are 20 nm.

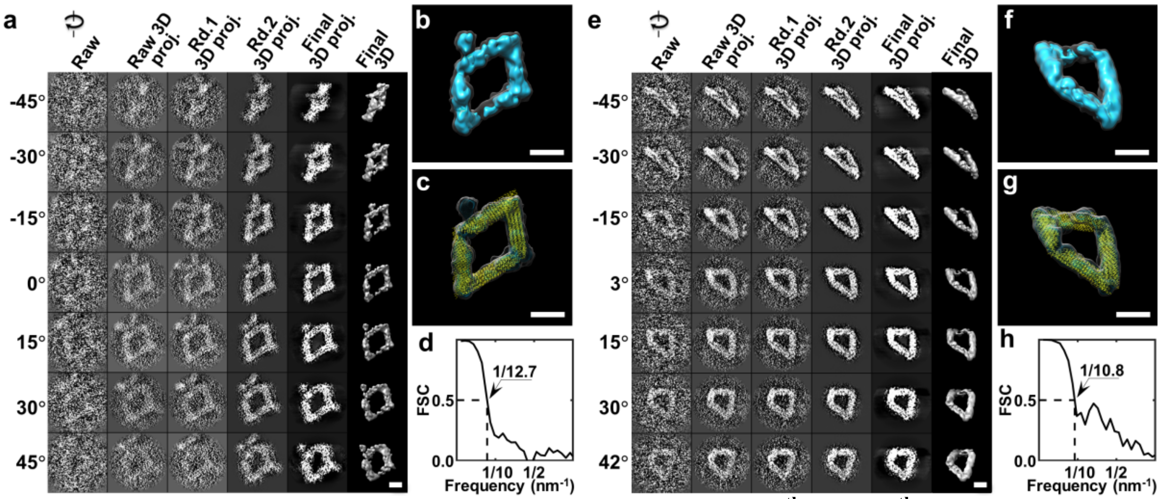

Supplementary Fig. 45 | IPET reconstruction processes on the 87th and 88th DNA origami Bennett linkage (a) Seven representative tilt views (first column), their corresponding projections on the intermediate 3D reconstructions from major iterations (second to fifth columns), and the 3D density map (sixth column) of the 87th particle of the DNA origami Bennett linkage are shown. **(b)** The final 3D density map. **(c)** The density map displays the overall conformation of the DNA origami Bennett linkage. A new conformation of DNA origami Bennett linkage was obtained (yellow ribbon) by flexible docking the Bennett linkage model into the density. **(d)** The FSC curve shows that the resolution of the final 3D reconstruction was ~12.7 nm. (e–h) The 3D density map of the 88th individual Bennett linkage was reconstructed from the tilt images using IPET. The FSC analysis showed that the 3D reconstruction resolution was ~10.8 nm. Scale bars are 20 nm.

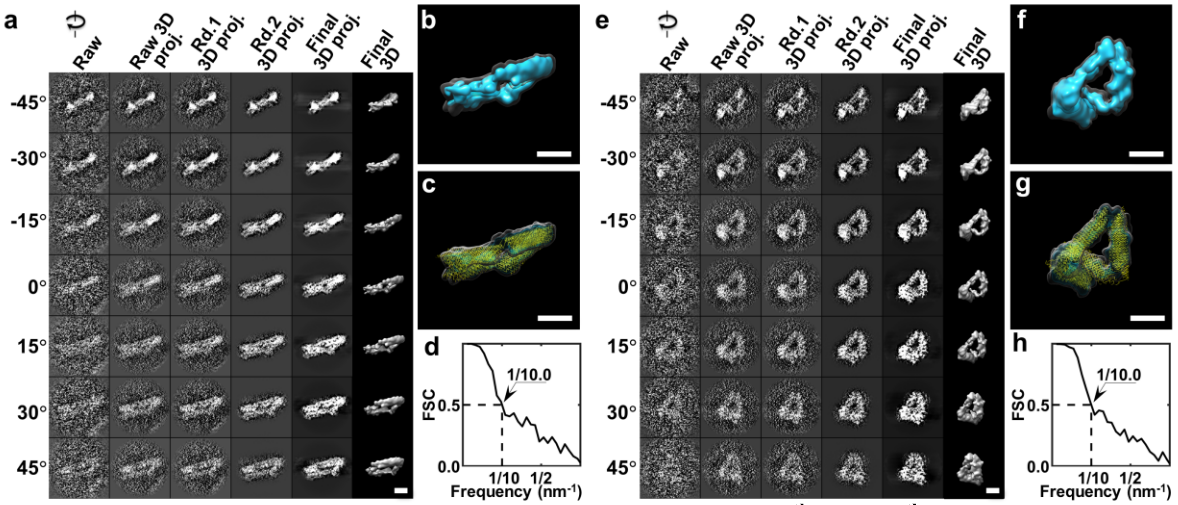

Supplementary Fig. 46 | IPET reconstruction processes on the 89th and 90th DNA origami Bennett linkage (a) Seven representative tilt views (first column), their corresponding projections on the intermediate 3D reconstructions from major iterations (second to fifth columns), and the 3D density map (sixth column) of the 89th particle of the DNA origami Bennett linkage are shown. **(b)** The final 3D density map. **(c)** The density map displays the overall conformation of the DNA origami Bennett linkage. A new conformation of DNA origami Bennett linkage was obtained (yellow ribbon) by flexible docking the Bennett linkage model into the density. **(d)** The FSC curve shows that the resolution of the final 3D reconstruction was ~10.0 nm. **(e–h)** The 3D density map of the 90th individual Bennett linkage was reconstructed from the tilt images using IPET. The FSC analysis showed that the 3D reconstruction resolution was ~10.0 nm. Scale bars are 20 nm.

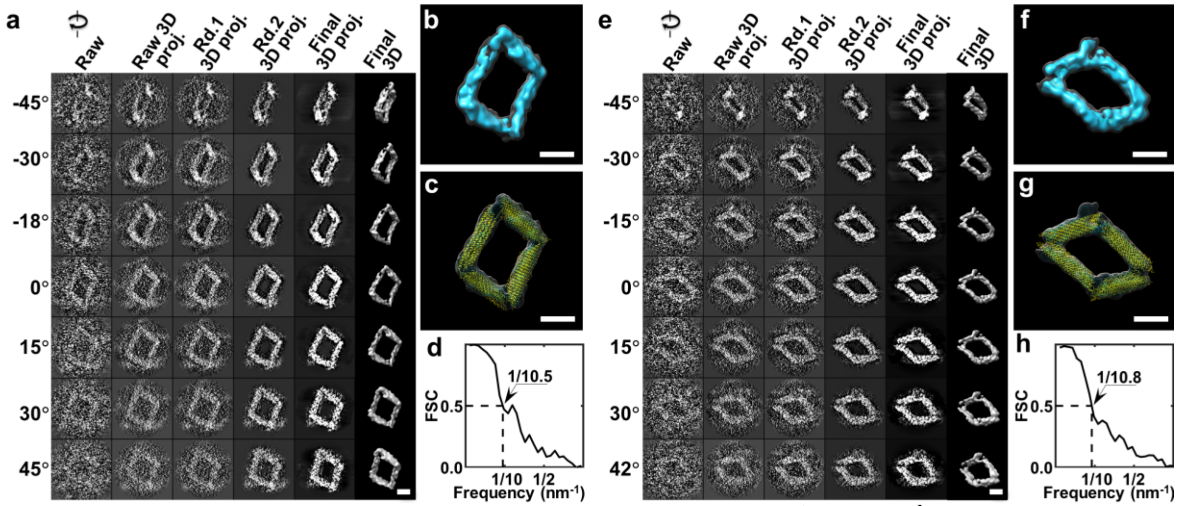

Supplementary Fig. 47 | IPET reconstruction processes on the 91st and 92nd DNA origami Bennett linkage (a) Seven representative tilt views (first column), their corresponding projections on the intermediate 3D reconstructions from major iterations (second to fifth columns), and the 3D density map (sixth column) of the 91st particle of the DNA origami Bennett linkage are shown. **(b)** The final 3D density map. **(c)** The density map displays the overall conformation of the DNA origami Bennett linkage. A new conformation of DNA origami Bennett linkage was obtained (yellow ribbon) by flexible docking the Bennett linkage model into the density. **(d)** The FSC curve shows that the resolution of the final 3D reconstruction was ~10.5 nm. **(e–h)** The 3D density map of the 92nd individual Bennett linkage was reconstructed from the tilt images using IPET. The FSC analysis showed that the 3D reconstruction resolution was ~10.8 nm. Scale bars are 20 nm.

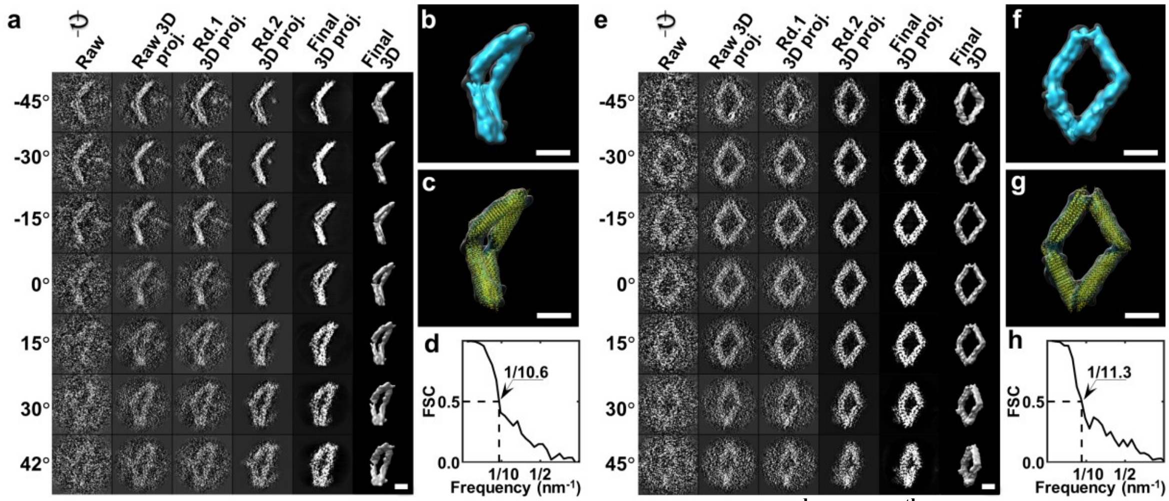

Supplementary Fig. 48 | IPET reconstruction processes on the 93rd and 94th DNA origami Bennett linkage (a) Seven representative tilt views (first column), their corresponding projections on the intermediate 3D reconstructions from major iterations (second to fifth columns), and the 3D density map (sixth column) of the 93rd particle of the DNA origami Bennett linkage are shown. **(b)** The final 3D density map. **(c)** The density map displays the overall conformation of the DNA origami Bennett linkage. A new conformation of DNA origami Bennett linkage was obtained (yellow ribbon) by flexible docking the Bennett linkage model into the density. **(d)** The FSC curve shows that the resolution of the final 3D reconstruction was ~10.6 nm. **(e–h)** The 3D density map of the 94th individual Bennett linkage was reconstructed from the tilt images using IPET. The FSC analysis showed that the 3D reconstruction resolution was ~11.3 nm. Scale bars are 20 nm.

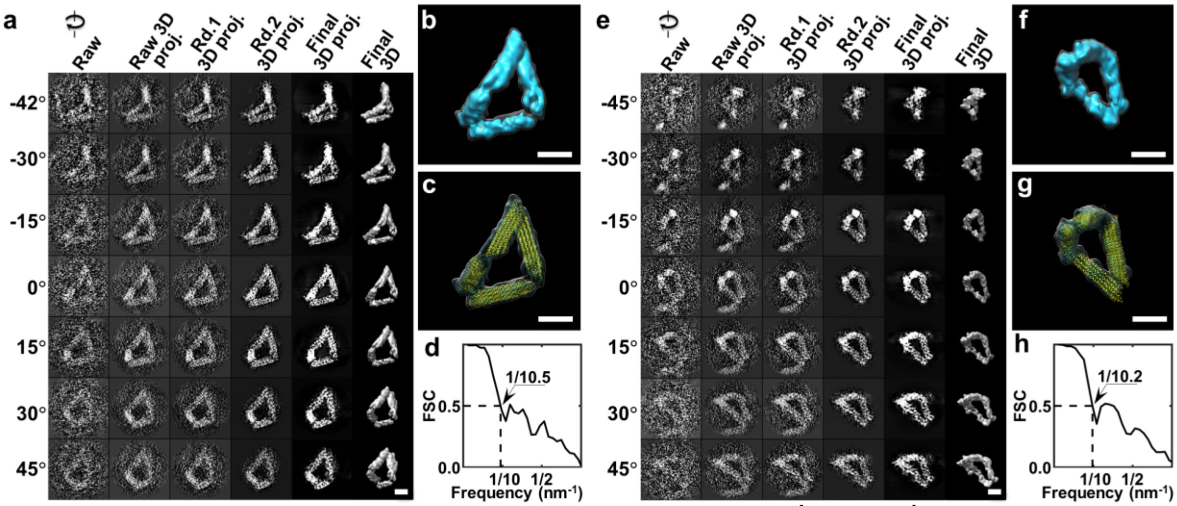

Supplementary Fig. 49 | IPET reconstruction processes on the 95th and 96th DNA origami Bennett linkage (a) Seven representative tilt views (first column), their corresponding projections on the intermediate 3D reconstructions from major iterations (second to fifth columns), and the 3D density map (sixth column) of the 95th particle of the DNA origami Bennett linkage are shown. **(b)** The final 3D density map. **(c)** The density map displays the overall conformation of the DNA origami Bennett linkage. A new conformation of DNA origami Bennett linkage was obtained (yellow ribbon) by flexible docking the Bennett linkage model into the density. **(d)** The FSC curve shows that the resolution of the final 3D reconstruction was ~10.5 nm. **(e–h)** The 3D density map of the 96th individual Bennett linkage was reconstructed from the tilt images using IPET. The FSC analysis showed that the 3D reconstruction resolution was ~10.2 nm. Scale bars are 20 nm.

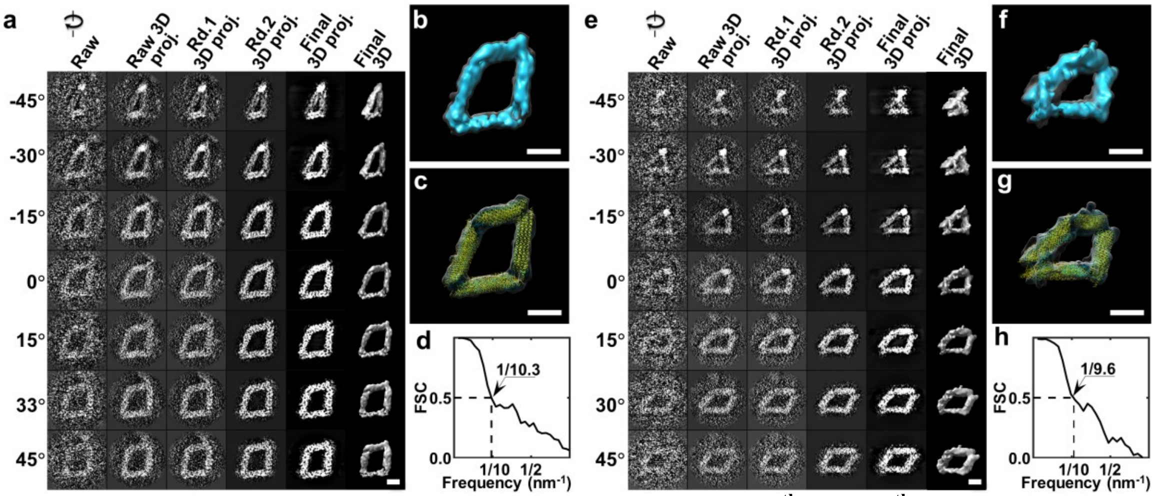

Supplementary Fig. 50 | IPET reconstruction processes on the 97th and 98th DNA origami Bennett linkage (a) Seven representative tilt views (first column), their corresponding projections on the intermediate 3D reconstructions from major iterations (second to fifth columns), and the 3D density map (sixth column) of the 97th particle of the DNA origami Bennett linkage are shown. **(b)** The final 3D density map. **(c)** The density map displays the overall conformation of the DNA origami Bennett linkage. A new conformation of DNA origami Bennett linkage was obtained (yellow ribbon) by flexible docking the Bennett linkage model into the density. **(d)** The FSC curve shows that the resolution of the final 3D reconstruction was ~10.3 nm. **(e–h)** The 3D density map of the 98th individual Bennett linkage was reconstructed from the tilt images using IPET. The FSC analysis showed that the 3D reconstruction resolution was ~9.6 nm. Scale bars are 20 nm.

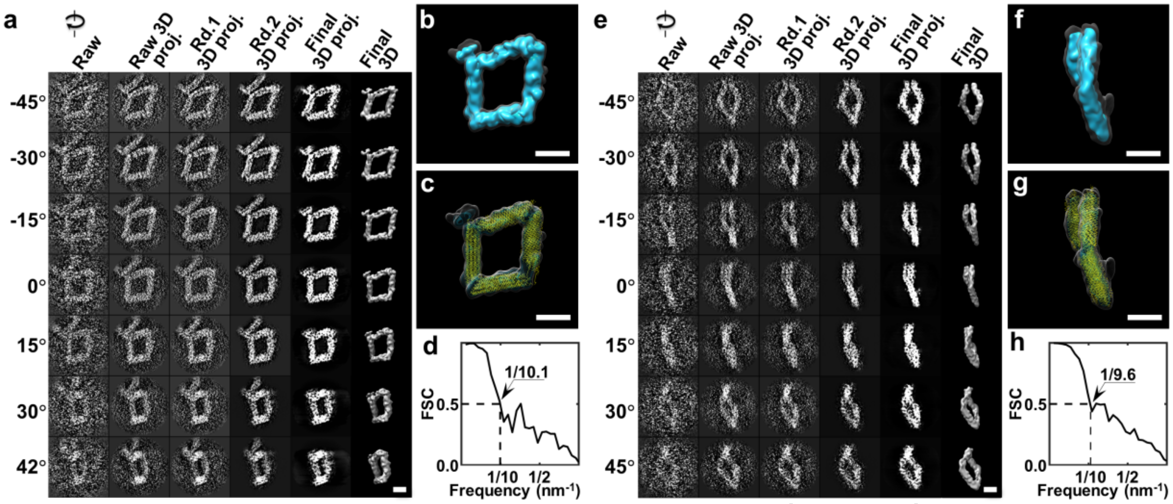

Supplementary Fig. 51 | IPET reconstruction processes on the 99th and 100th DNA origami Bennett linkage (a) Seven representative tilt views (first column), their corresponding projections on the intermediate 3D reconstructions from major iterations (second to fifth columns), and the 3D density map (sixth column) of the 99th particle of the DNA origami Bennett linkage are shown. **(b)** The final 3D density map. **(c)** The density map displays the overall conformation of the DNA origami Bennett linkage. A new conformation of DNA origami Bennett linkage was obtained (yellow ribbon) by flexible docking the Bennett linkage model into the density. **(d)** The FSC curve shows that the resolution of the final 3D reconstruction was ~10.1 nm. **(e–h)** The 3D density map of the 100th individual Bennett linkage was reconstructed from the tilt images using IPET. The FSC analysis showed that the 3D reconstruction resolution was ~9.6 nm. Scale bars are 20 nm.

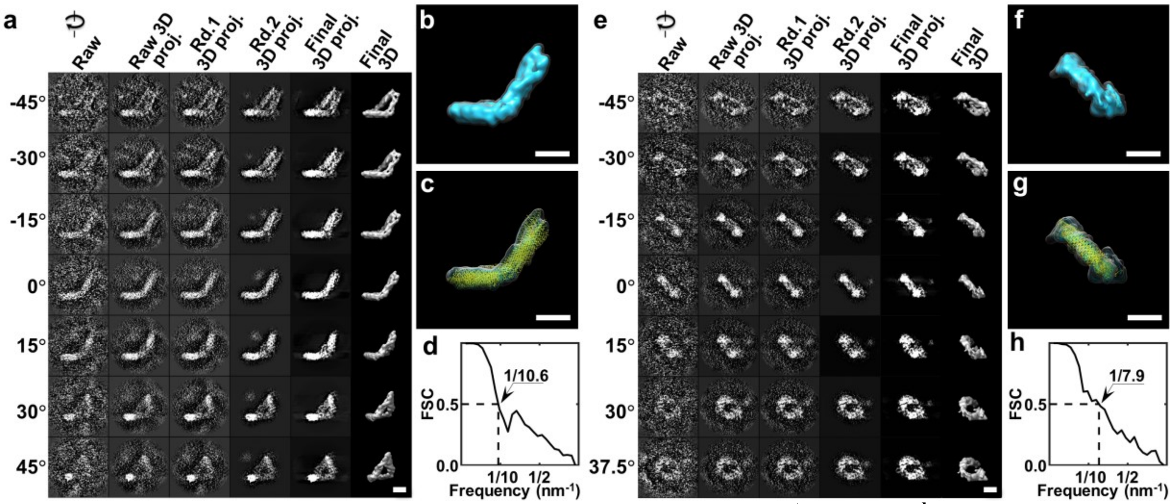

Supplementary Fig. 52 | IPET reconstruction processes on the 101st and 102nd DNA origami Bennett linkage (a) Seven representative tilt views (first column), their corresponding projections on the intermediate 3D reconstructions from major iterations (second to fifth columns), and the 3D density map (sixth column) of the 101st particle of the DNA origami Bennett linkage are shown. **(b)** The final 3D density map. **(c)** The density map displays the overall conformation of the DNA origami Bennett linkage. A new conformation of DNA origami Bennett linkage was obtained (yellow ribbon) by flexible docking the Bennett linkage model into the density. **(d)** The FSC curve shows that the resolution of the final 3D reconstruction was ~10.6 nm. **(e–h)** The 3D density map of the 102nd individual Bennett linkage was reconstructed from the tilt images using IPET. The FSC analysis showed that the 3D reconstruction resolution was ~7.9 nm. Scale bars are 20 nm.

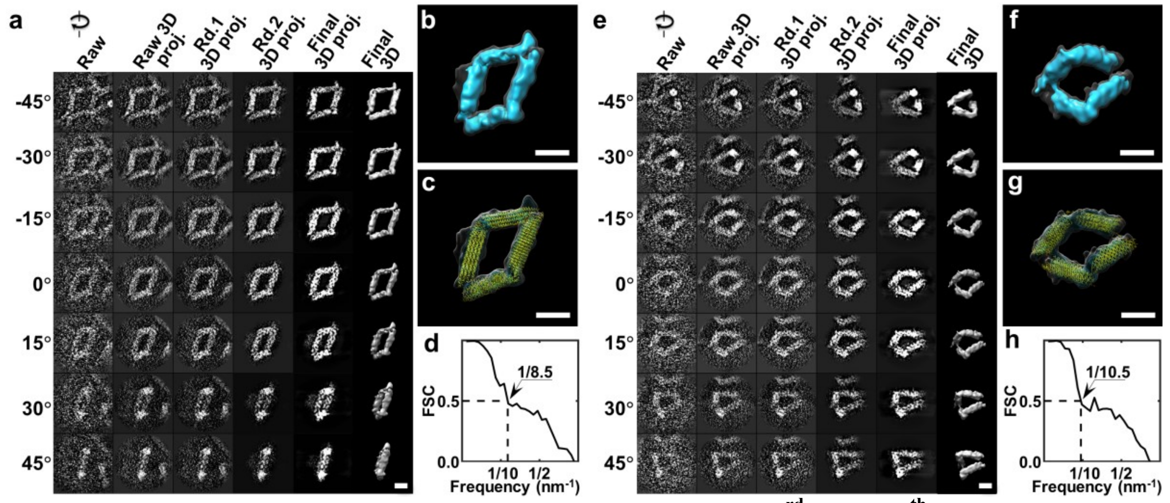

Supplementary Fig. 53 | IPET reconstruction processes on the 103rd and 105th DNA origami Bennett linkage (a) Seven representative tilt views (first column), their corresponding projections on the intermediate 3D reconstructions from major iterations (second to fifth columns), and the 3D density map (sixth column) of the 103rd particle of the DNA origami Bennett linkage are shown. **(b)** The final 3D density map. **(c)** The density map displays the overall conformation of the DNA origami Bennett linkage. A new conformation of DNA origami Bennett linkage was obtained (yellow ribbon) by flexible docking the Bennett linkage model into the density. **(d)** The FSC curve shows that the resolution of the final 3D reconstruction was ~8.5 nm. **(e–h)** The 3D density map of the 104th individual Bennett linkage was reconstructed from the tilt images using IPET. The FSC analysis showed that the 3D reconstruction resolution was ~10.5 nm. Scale bars are 20 nm.

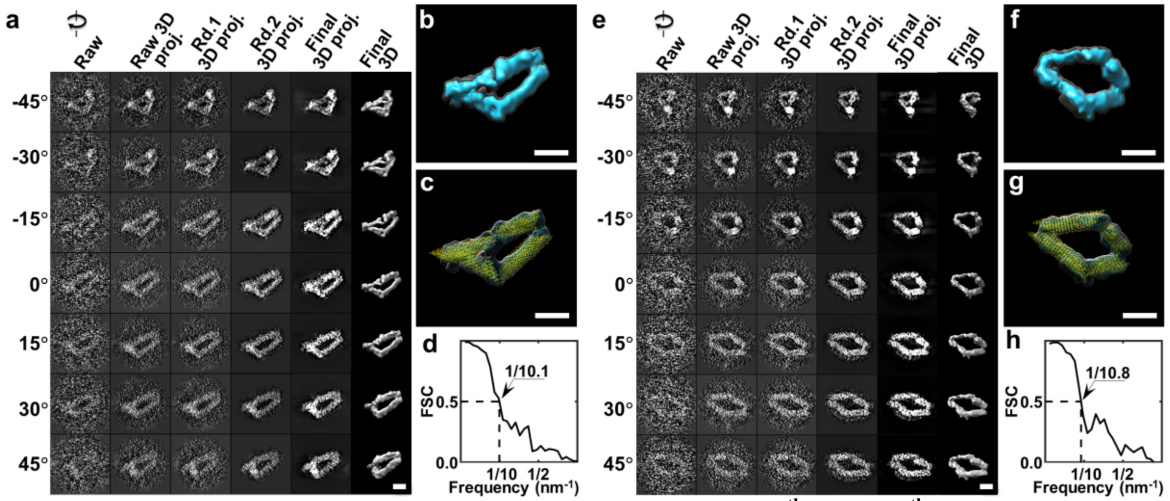

Supplementary Fig. 54 | IPET reconstruction processes on the 105th and 106th DNA origami Bennett linkage (a) Seven representative tilt views (first column), their corresponding projections on the intermediate 3D reconstructions from major iterations (second to fifth columns), and the 3D density map (sixth column) of the 105th particle of the DNA origami Bennett linkage are shown. **(b)** The final 3D density map. **(c)** The density map displays the overall conformation of the DNA origami Bennett linkage. A new conformation of DNA origami Bennett linkage was obtained (yellow ribbon) by flexible docking the Bennett linkage model into the density. **(d)** The FSC curve shows that the resolution of the final 3D reconstruction was ~10.1 nm. **(e–h)** The 3D density map of the 106th individual Bennett linkage was reconstructed from the tilt images using IPET. The FSC analysis showed that the 3D reconstruction resolution was ~10.8 nm. Scale bars are 20 nm.

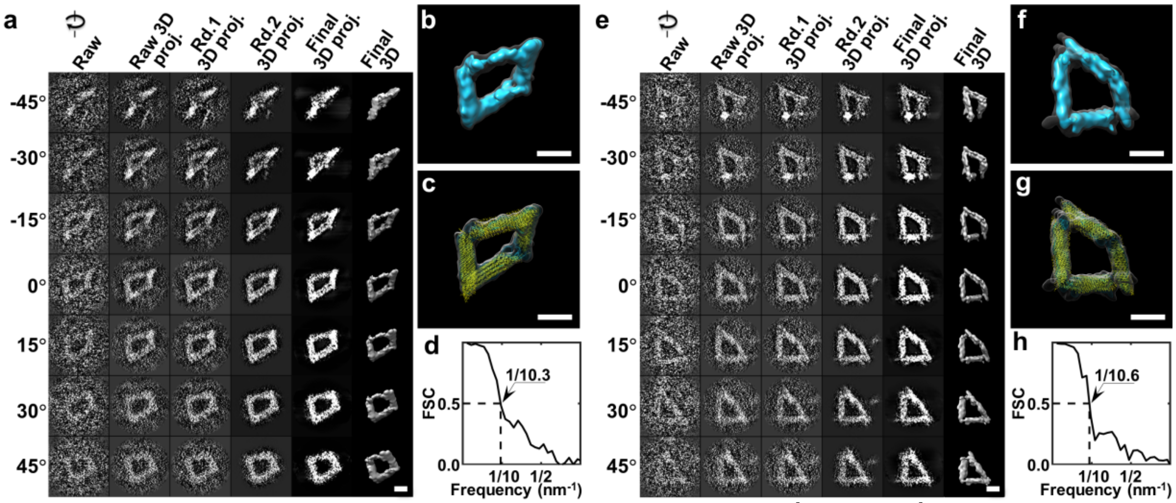

Supplementary Fig. 55 | IPET reconstruction processes on the 107th and 108th DNA origami Bennett linkage (a) Seven representative tilt views (first column), their corresponding projections on the intermediate 3D reconstructions from major iterations (second to fifth columns), and the 3D density map (sixth column) of the 107th particle of the DNA origami Bennett linkage are shown. **(b)** The final 3D density map. **(c)** The density map displays the overall conformation of the DNA origami Bennett linkage. A new conformation of DNA origami Bennett linkage was obtained (yellow ribbon) by flexible docking the Bennett linkage model into the density. **(d)** The FSC curve shows that the resolution of the final 3D reconstruction was ~10.3 nm. **(e–h)** The 3D density map of the 108th individual Bennett linkage was reconstructed from the tilt images using IPET. The FSC analysis showed that the 3D reconstruction resolution was ~10.6 nm. Scale bars are 20 nm.

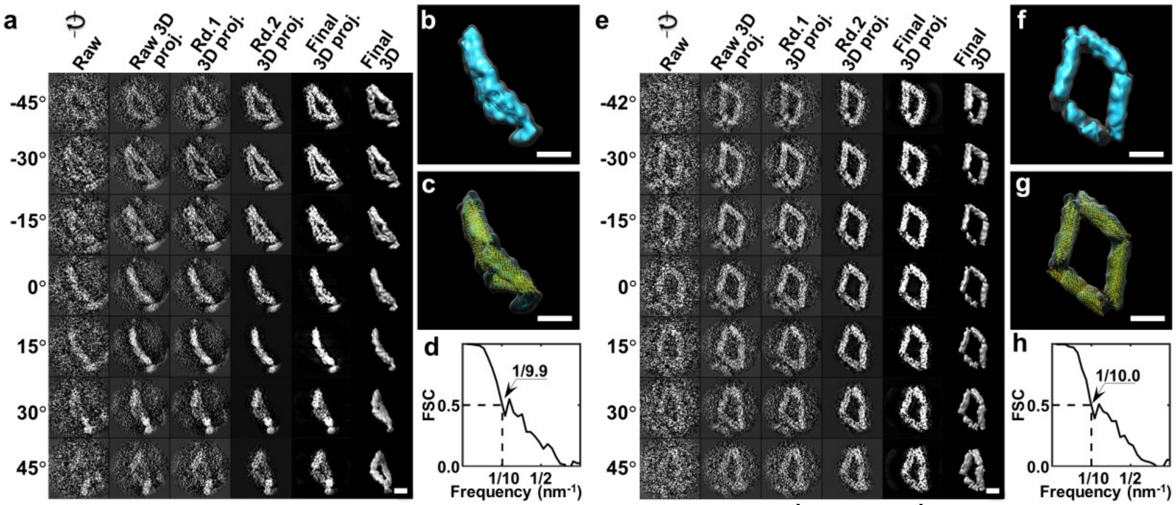

Supplementary Fig. 56 | IPET reconstruction processes on the 109th and 110th DNA origami Bennett linkage (a) Seven representative tilt views (first column), their corresponding projections on the intermediate 3D reconstructions from major iterations (second to fifth columns), and the 3D density map (sixth column) of the 109th particle of the DNA origami Bennett linkage are shown. **(b)** The final 3D density map. **(c)** The density map displays the overall conformation of the DNA origami Bennett linkage. A new conformation of DNA origami Bennett linkage was obtained (yellow ribbon) by flexible docking the Bennett linkage model into the density. **(d)** The FSC curve shows that the resolution of the final 3D reconstruction was ~9.9 nm. **(e–h)** The 3D density map of the 110th individual Bennett linkage was reconstructed from the tilt images using IPET. The FSC analysis showed that the 3D reconstruction resolution was ~10.0 nm. Scale bars are 20 nm.

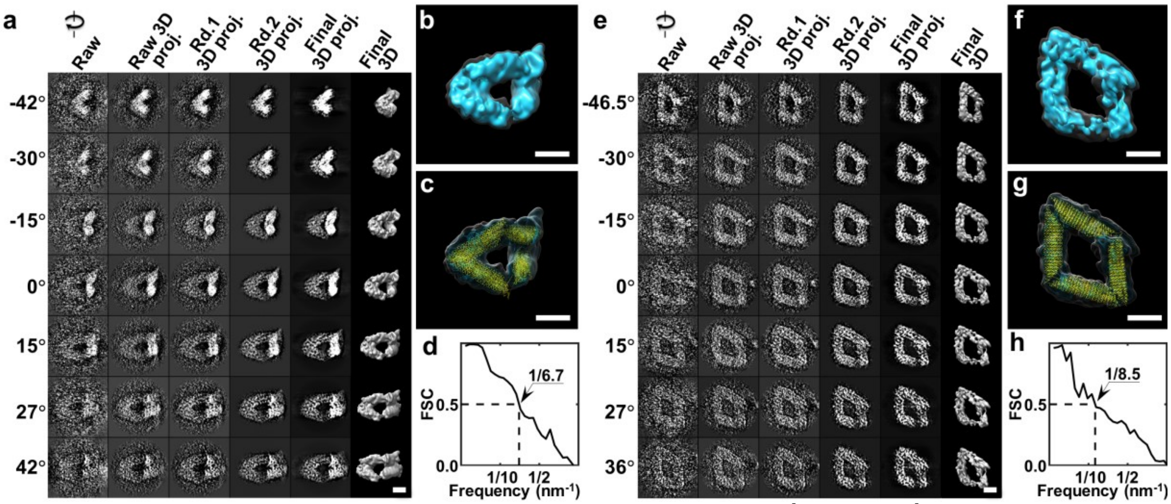

Supplementary Fig. 57 | IPET reconstruction processes on the 111th and 112th DNA origami Bennett linkage (a) Seven representative tilt views (first column), their corresponding projections on the intermediate 3D reconstructions from major iterations (second to fifth columns), and the 3D density map (sixth column) of the 111th particle of the DNA origami Bennett linkage are shown. **(b)** The final 3D density map. **(c)** The density map displays the overall conformation of the DNA origami Bennett linkage. A new conformation of DNA origami Bennett linkage was obtained (yellow ribbon) by flexible docking the Bennett linkage model into the density. **(d)** The FSC curve shows that the resolution of the final 3D reconstruction was ~6.7 nm. **(e–h)** The 3D density map of the 112th individual Bennett linkage was reconstructed from the tilt images using IPET. The FSC analysis showed that the 3D reconstruction resolution was ~8.5 nm. Scale bars are 20 nm.

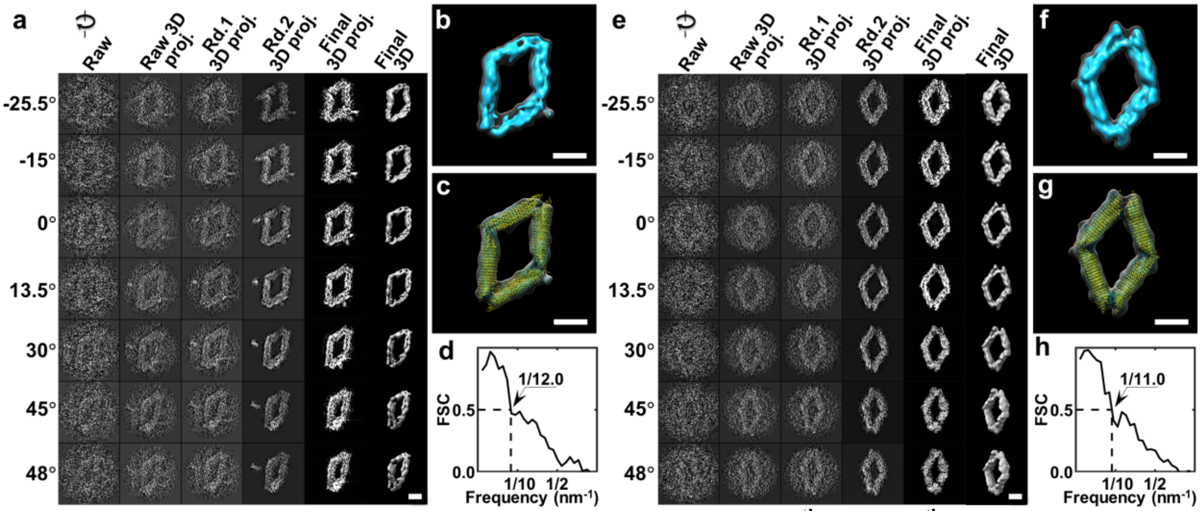

Supplementary Fig. 58 | IPET reconstruction processes on the 113th and 114th DNA origami Bennett linkage (a) Seven representative tilt views (first column), their corresponding projections on the intermediate 3D reconstructions from major iterations (second to fifth columns), and the 3D density map (sixth column) of the 113th particle of the DNA origami Bennett linkage are shown. **(b)** The final 3D density map. **(c)** The density map displays the overall conformation of the DNA origami Bennett linkage. A new conformation of DNA origami Bennett linkage was obtained (yellow ribbon) by flexible docking the Bennett linkage model into the density. **(d)** The FSC curve shows that the resolution of the final 3D reconstruction was ~12.0 nm. **(e–h)** The 3D density map of the 114th individual Bennett linkage was reconstructed from the tilt images using IPET. The FSC analysis showed that the 3D reconstruction resolution was ~11.0 nm. Scale bars are 20 nm.

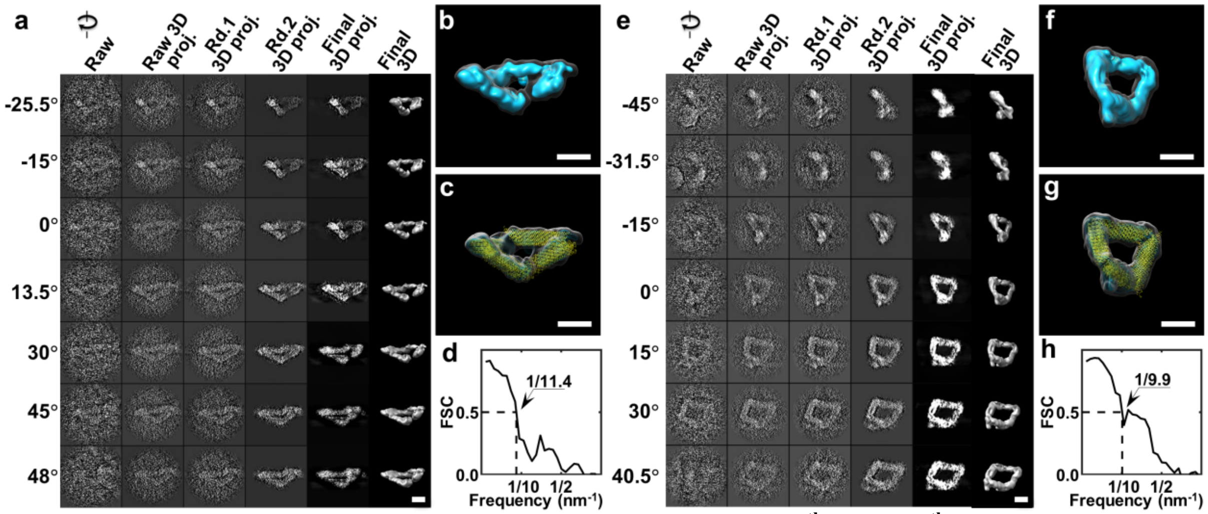

Supplementary Fig. 59 | IPET reconstruction processes on the 115th and 116th DNA origami Bennett linkage (a) Seven representative tilt views (first column), their corresponding projections on the intermediate 3D reconstructions from major iterations (second to fifth columns), and the 3D density map (sixth column) of the 115th particle of the DNA origami Bennett linkage are shown. **(b)** The final 3D density map. **(c)** The density map displays the overall conformation of the DNA origami Bennett linkage. A new conformation of DNA origami Bennett linkage was obtained (yellow ribbon) by flexible docking the Bennett linkage model into the density. **(d)** The FSC curve shows that the resolution of the final 3D reconstruction was ~11.4 nm. **(e–h)** The 3D density map of the 116th individual Bennett linkage was reconstructed from the tilt images using IPET. The FSC analysis showed that the 3D reconstruction resolution was ~9.9 nm. Scale bars are 20 nm.

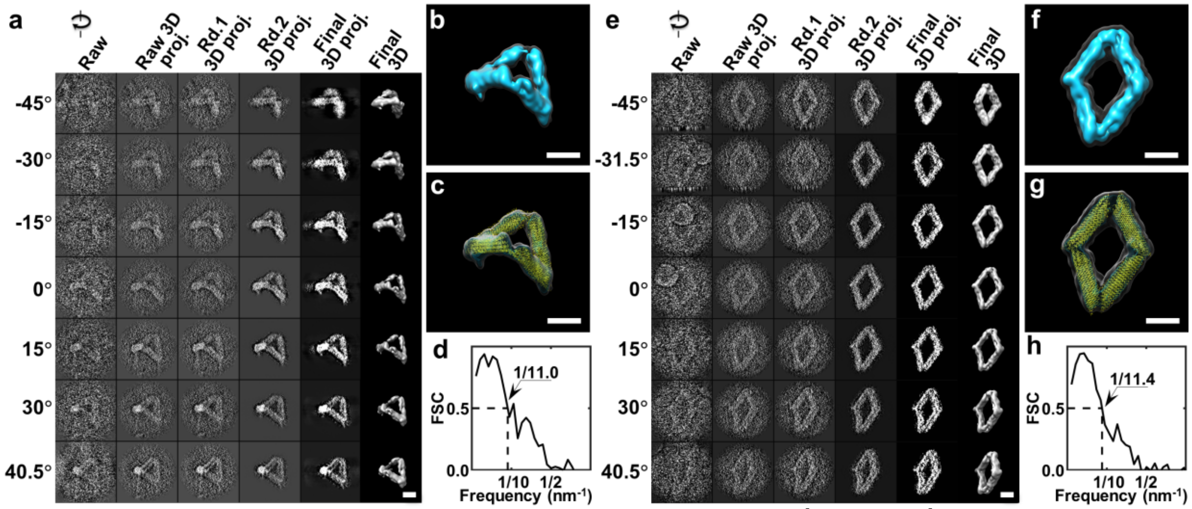

Supplementary Fig. 60 | IPET reconstruction processes on the 117th and 118th DNA origami Bennett linkage (a) Seven representative tilt views (first column), their corresponding projections on the intermediate 3D reconstructions from major iterations (second to fifth columns), and the 3D density map (sixth column) of the 117th particle of the DNA origami Bennett linkage are shown. **(b)** The final 3D density map. **(c)** The density map displays the overall conformation of the DNA origami Bennett linkage. A new conformation of DNA origami Bennett linkage was obtained (yellow ribbon) by flexible docking the Bennett linkage model into the density. **(d)** The FSC curve shows that the resolution of the final 3D reconstruction was ~11.0 nm. **(e–h)** The 3D density map of the 118th individual Bennett linkage was reconstructed from the tilt images using IPET. The FSC analysis showed that the 3D reconstruction resolution was ~11.4 nm. Scale bars are 20 nm.

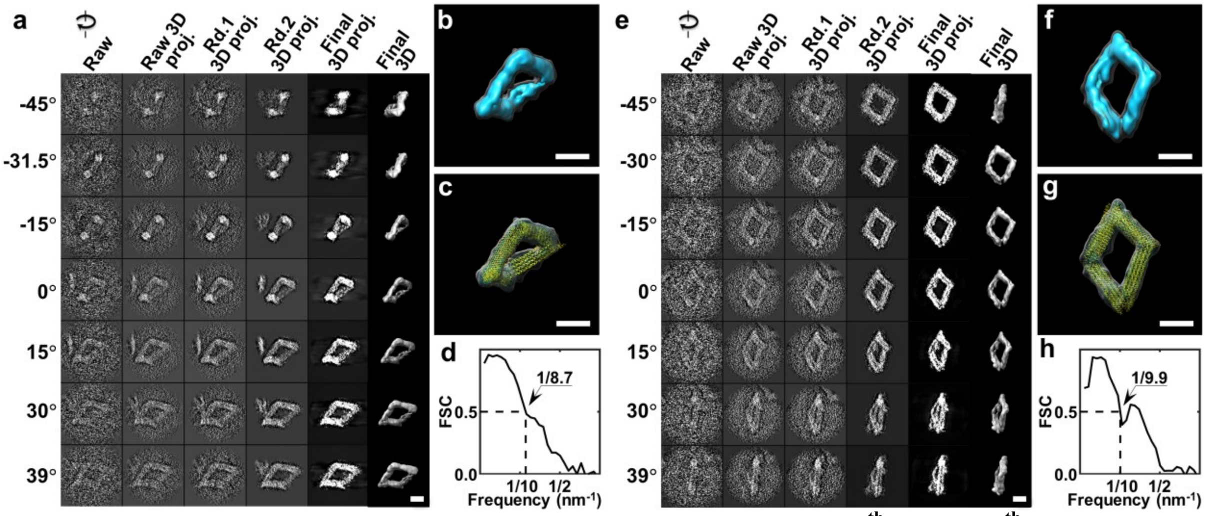

Supplementary Fig. 61 | IPET reconstruction processes on the 119th and 120th DNA origami Bennett linkage (a) Seven representative tilt views (first column), their corresponding projections on the intermediate 3D reconstructions from major iterations (second to fifth columns), and the 3D density map (sixth column) of the 119th particle of the DNA origami Bennett linkage are shown. **(b)** The final 3D density map. **(c)** The density map displays the overall conformation of the DNA origami Bennett linkage. A new conformation of DNA origami Bennett linkage was obtained (yellow ribbon) by flexible docking the Bennett linkage model into the density. **(d)** The FSC curve shows that the resolution of the final 3D reconstruction was ~8.7 nm. **(e–h)** The 3D density map of the 120th individual Bennett linkage was reconstructed from the tilt images using IPET. The FSC analysis showed that the 3D reconstruction resolution was ~9.9 nm. Scale bars are 20 nm.

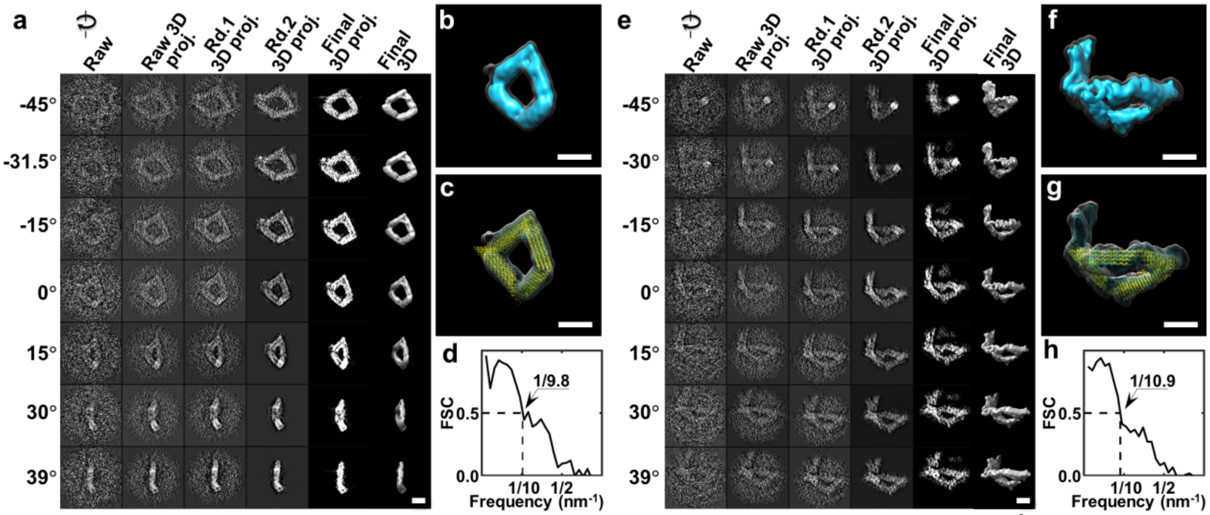

Supplementary Fig. 62 | IPET reconstruction processes on the 121st and 122nd DNA origami Bennett linkage (a) Seven representative tilt views (first column), their corresponding projections on the intermediate 3D reconstructions from major iterations (second to fifth columns), and the 3D density map (sixth column) of the 121st particle of the DNA origami Bennett linkage are shown. **(b)** The final 3D density map. **(c)** The density map displays the overall conformation of the DNA origami Bennett linkage. A new conformation of DNA origami Bennett linkage was obtained (yellow ribbon) by flexible docking the Bennett linkage model into the density. **(d)** The FSC curve shows that the resolution of the final 3D reconstruction was ~9.8 nm. **(e–h)** The 3D density map of the 122nd individual Bennett linkage was reconstructed from the tilt images using IPET. The FSC analysis showed that the 3D reconstruction resolution was ~10.9 nm. Scale bars are 20 nm.

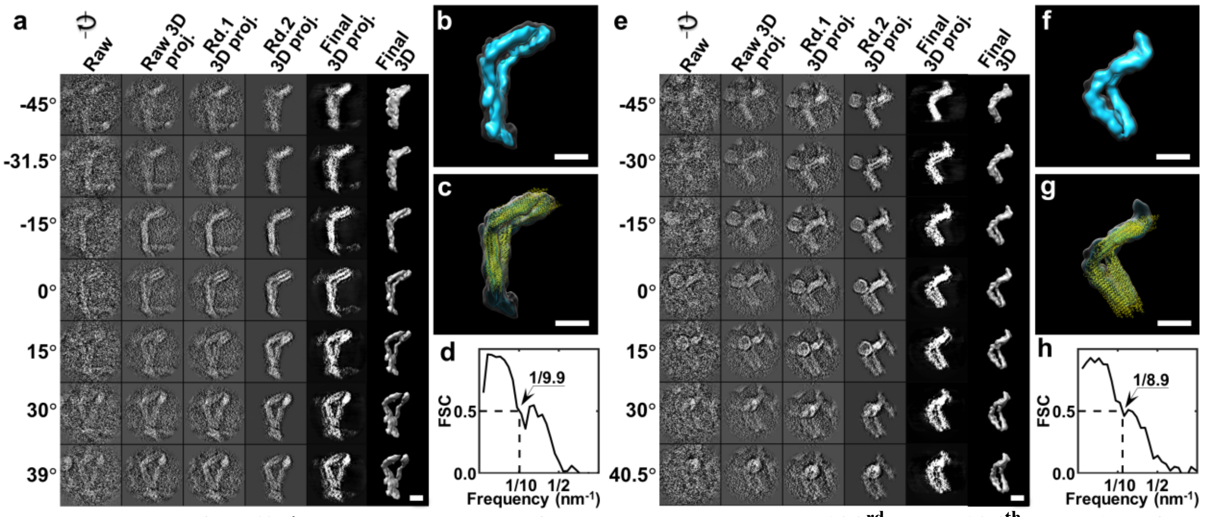

Supplementary Fig. 63 | IPET reconstruction processes on the 123rd and 124th DNA origami Bennett linkage (a) Seven representative tilt views (first column), their corresponding projections on the intermediate 3D reconstructions from major iterations (second to fifth columns), and the 3D density map (sixth column) of the 123rd particle of the DNA origami Bennett linkage are shown. **(b)** The final 3D density map. **(c)** The density map displays the overall conformation of the DNA origami Bennett linkage. A new conformation of DNA origami Bennett linkage was obtained (yellow ribbon) by flexible docking the Bennett linkage model into the density. **(d)** The FSC curve shows that the resolution of the final 3D reconstruction was ~9.9 nm. **(e–h)** The 3D density map of the 124th individual Bennett linkage was reconstructed from the tilt images using IPET. The FSC analysis showed that the 3D reconstruction resolution was ~8.9 nm. Scale bars are 20 nm.

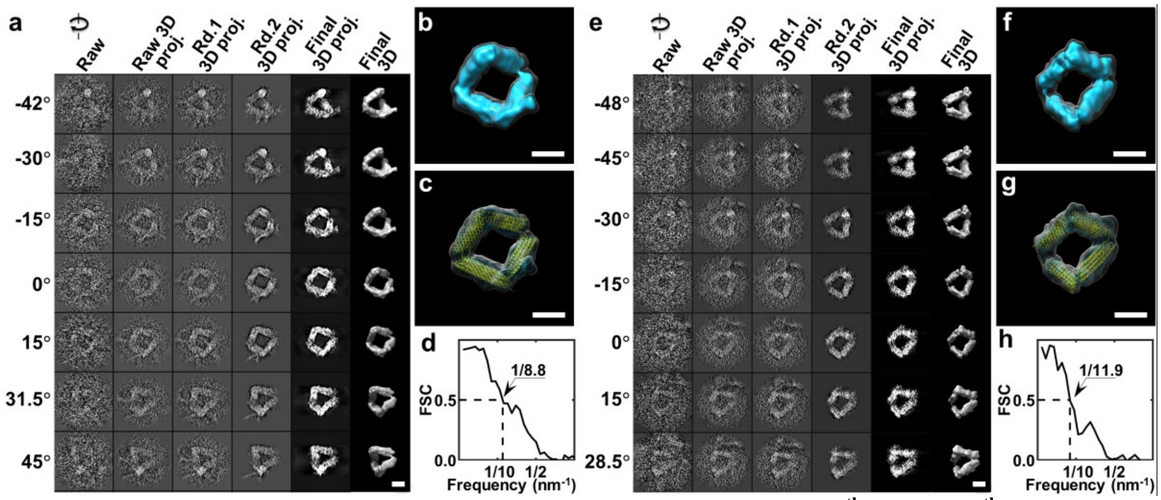

Supplementary Fig. 64 | IPET reconstruction processes on the 125th and 126th DNA origami Bennett linkage (a) Seven representative tilt views (first column), their corresponding projections on the intermediate 3D reconstructions from major iterations (second to fifth columns), and the 3D density map (sixth column) of the 125th particle of the DNA origami Bennett linkage are shown. **(b)** The final 3D density map. **(c)** The density map displays the overall conformation of the DNA origami Bennett linkage. A new conformation of DNA origami Bennett linkage was obtained (yellow ribbon) by flexible docking the Bennett linkage model into the density. **(d)** The FSC curve shows that the resolution of the final 3D reconstruction was ~8.8 nm. **(e–h)** The 3D density map of the 126th individual Bennett linkage was reconstructed from the tilt images using IPET. The FSC analysis showed that the 3D reconstruction resolution was ~11.9 nm. Scale bars are 20 nm.

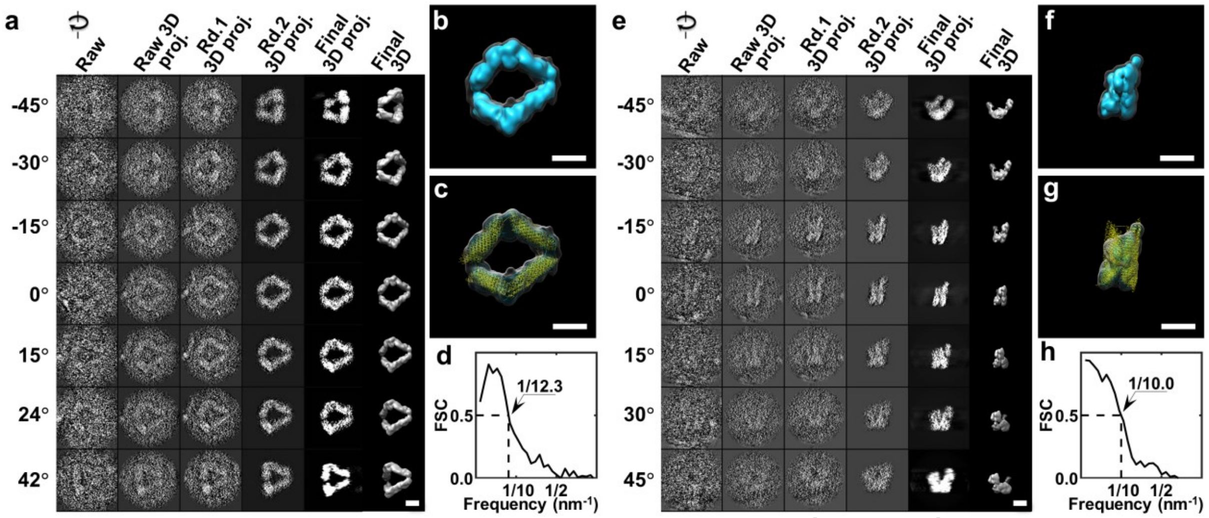

Supplementary Fig. 65 | IPET reconstruction processes on the 127th and 128th DNA origami Bennett linkage (a) Seven representative tilt views (first column), their corresponding projections on the intermediate 3D reconstructions from major iterations (second to fifth columns), and the 3D density map (sixth column) of the 127th particle of the DNA origami Bennett linkage are shown. **(b)** The final 3D density map. **(c)** The density map displays the overall conformation of the DNA origami Bennett linkage. A new conformation of DNA origami Bennett linkage was obtained (yellow ribbon) by flexible docking the Bennett linkage model into the density. **(d)** The FSC curve shows that the resolution of the final 3D reconstruction was ~12.3 nm. **(e–h)** The 3D density map of the 128th individual Bennett linkage was reconstructed from the tilt images using IPET. The FSC analysis showed that the 3D reconstruction resolution was ~10.0 nm. Scale bars are 20 nm.

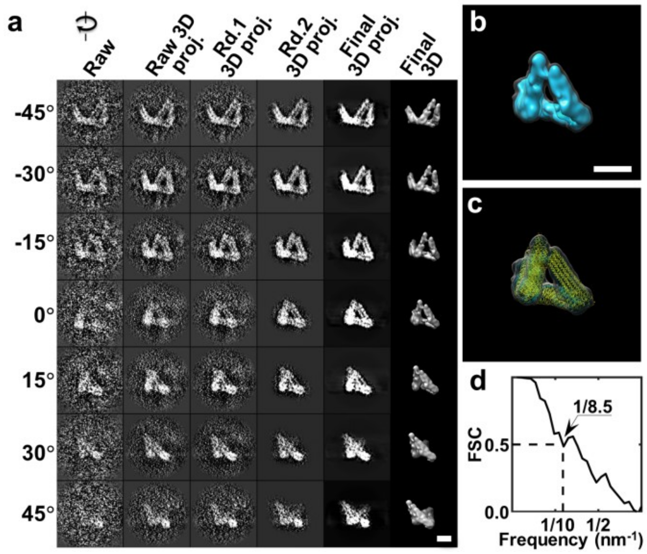

Supplementary Fig. 66 | IPET reconstruction processes on the 129th DNA origami Bennett linkage (a) Seven representative tilt views (first column), their corresponding projections on the intermediate 3D reconstructions from major iterations (second to fifth columns), and the 3D density map (sixth column) of the 129th particle of the DNA origami Bennett linkage are shown. **(b)** The final 3D density map. **(c)** The density map displays the overall conformation of the DNA origami Bennett linkage. A new conformation of DNA origami Bennett linkage was obtained (yellow ribbon) by flexible docking the Bennett linkage model into the density. **(d)** The FSC curve shows that the resolution of the final 3D reconstruction was ~8.5 Å. Scale bars are 20 nm.

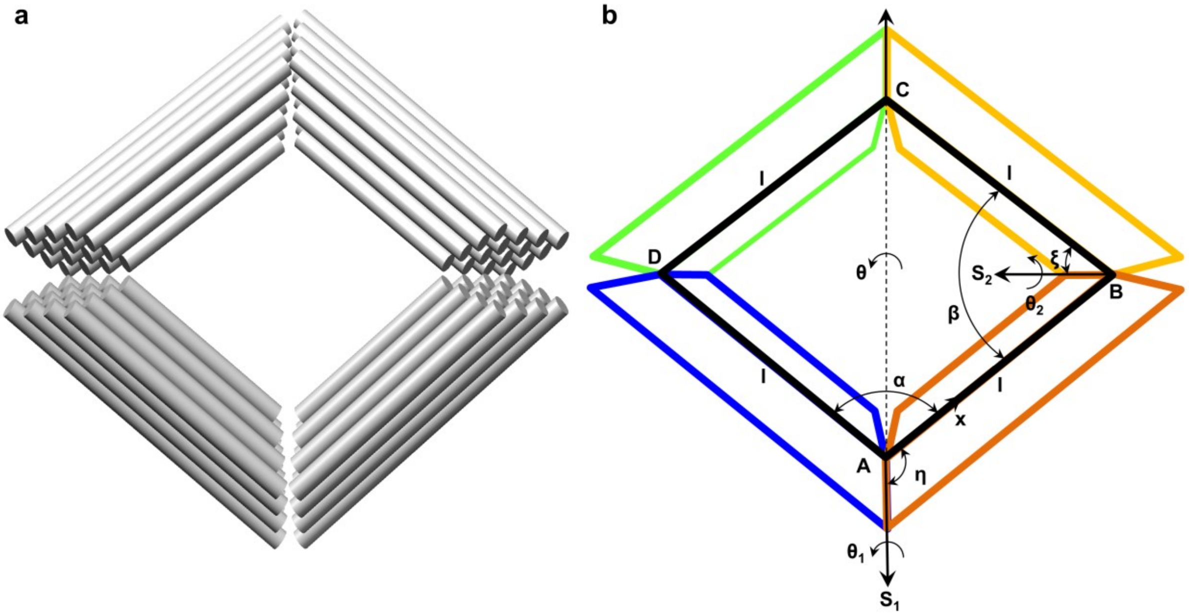

Supplementary Fig. 67 | Schematics of DNA origami Bennett linkage (a) Model of DNA origami Bennett linkage. **(b)** Kinematics model of DNA origami Bennett linkage

Supplementary Table 1. The parameters used for IPET 3D reconstructions

#	EMDB#[1]	TEM[2]	CCD[3]	Mag.[3]	Apix[4] (Å)	Dose/img.[5] ($e^-/Å^2$)	Dose/set[6] ($e^-/Å^2$)	Acq. angle range[7]	Total img.[8]	Reconst. angle range[9]	Cont.[10]	Resol.[11] (nm)	*α* (°)	*β* (°)	*ϑ* (°)	*φ* (°)	RSS *ang.*[12] (°)	Supp. Fig. [13]
1	EMD-7155	Zeiss 120	UltraScan	50 kX	4.8	2.01	122.56	-45° to +45°	61	-45° to 45°	0.299	6.41	101.8	66.4	136.2	119.7	181.3	S. Fig. 2
2	EMD-7156	Zeiss 120	UltraScan	50 kX	4.8	1.69	103.14	-45° to +45°	61	-45° to 45°	0.311	6.29	118.2	59.8	162.2	151.8	222.1	S. Fig. 2
3	EMD-7157	Zeiss 120	UltraScan	50 kX	4.8	1.73	105.58	-45° to +45°	61	-45° to 45°	0.335	10.23	108.2	70.9	162.0	161.1	228.5	S. Fig. 3
4	EMD-7165	Zeiss 120	UltraScan	50 kX	4.8	1.91	116.34	-45° to +45°	61	-45° to 45°	0.334	9.88	104.9	75.5	162.1	157.6	226.1	S. Fig. 3
5	EMD-7170	Zeiss 120	UltraScan	50 kX	4.8	1.65	100.85	-45° to +45°	61	-45° to 45°	0.322	10.94	113.8	62.0	154.2	141.9	209.5	S. Fig. 4
6	EMD-7175	Zeiss 120	UltraScan	50 kX	4.8	1.74	106.18	-45° to +45°	61	-45° to 45°	0.308	6.33	108.3	47.3	122.3	85.4	149.2	S. Fig. 4
7	EMD-7181	Zeiss 120	UltraScan	50 kX	4.8	2.85	173.72	-45° to +45°	61	-45° to 45°	0.261	6.15	127.7	35.4	137.4	85.6	161.9	S. Fig. 5
8	EMD-7184	Zeiss 120	UltraScan	50 kX	4.8	2.79	170.32	-45° to +45°	61	-45° to 45°	0.334	6.83	118.0	61.5	170.4	161.2	234.6	S. Fig. 5
9	EMD-7189	Zeiss 120	UltraScan	50 kX	4.8	2.75	167.67	-45° to +45°	61	-45° to 45°	0.402	6.05	102.3	41.4	107.9	68.2	127.7	S. Fig. 6
10	EMD-7193	Zeiss 120	UltraScan	50 kX	4.8	2.31	140.96	-45° to +45°	61	-45° to 45°	0.275	6.06	101.9	63.3	131.2	112.8	173.0	S. Fig. 6
11	EMD-7196	Zeiss 120	UltraScan	50 kX	4.8	2.83	172.61	-45° to +45°	61	-45° to 45°	0.342	5.94	102.6	46.9	115.7	75.8	138.3	S. Fig. 7
12	EMD-7200	Zeiss 120	UltraScan	50 kX	4.8	2.02	123.34	-45° to +45°	61	-45° to 45°	0.299	6.47	115.8	54.7	146.2	114.7	185.9	S. Fig. 7
13	EMD-7201	Zeiss 120	UltraScan	50 kX	4.8	0.97	58.92	-45° to +45°	61	-42° to 42°	0.310	9.25	105.0	57.9	133.7	104.7	169.8	S. Fig. 8
14	EMD-7203	Zeiss 120	UltraScan	50 kX	4.8	3.00	183.00	-45° to +45°	61	-45° to 45°	0.306	6.65	107.6	68.3	152.6	142.4	208.8	S. Fig. 8
15	EMD-7205	Zeiss 120	UltraScan	50 kX	4.8	2.86	174.22	-45° to +45°	61	-45° to 45°	0.280	5.72	108.6	69.9	163.9	156.3	226.5	S. Fig. 9
16	EMD-7207	Zeiss 120	UltraScan	50 kX	4.8	1.64	100.27	-45° to +45°	61	-45° to 45°	0.321	6.47	126.3	50.6	159.9	140.8	213.0	S. Fig. 9
17	EMD-7210	Zeiss 120	UltraScan	50 kX	4.8	1.68	102.41	-45° to +45°	61	-45° to 45°	0.292	6.36	116.8	61.2	161.8	149.6	220.4	S. Fig. 10
18	EMD-7214	Zeiss 120	UltraScan	50 kX	4.8	1.55	94.45	-45° to +45°	61	-45° to 45°	0.304	8.92	137.3	34.6	148.9	100.0	179.4	S. Fig. 10
19	EMD-7217	Zeiss 120	UltraScan	50 kX	4.8	1.53	93.43	-45° to +45°	61	-45° to 45°	0.271	6.78	124.4	48.5	152.4	122.3	195.4	S. Fig. 11
20	EMD-7219	Zeiss 120	UltraScan	50 kX	4.8	1.40	85.46	-45° to +45°	61	-45° to 45°	0.326	6.44	83.9	60.4	100.1	79.1	127.6	S. Fig. 11
21	EMD-7224	Zeiss 120	UltraScan	50 kX	4.8	1.43	87.02	-45° to +45°	61	-45° to 45°	0.390	9.46	152.9	24.2	162.4	126.9	206.1	S. Fig. 12
22	EMD-7228	Zeiss 120	UltraScan	50 kX	4.8	1.15	69.95	-45° to +45°	61	-45° to 45°	0.348	7.22	122.0	40.5	136.6	89.7	163.5	S. Fig. 12
23	EMD-7231	Zeiss 120	UltraScan	50 kX	4.8	1.17	71.61	-45° to +45°	61	-43.5° to 45°	0.326	6.26	119.8	58.4	162.7	152.7	223.1	S. Fig. 13
24	EMD-7235	Zeiss 120	UltraScan	50 kX	4.8	0.63	38.69	-45° to +45°	61	-45° to 45°	0.376	13.89	115.7	63.8	170.8	167.6	239.3	S. Fig. 13
25	EMD-7240	Zeiss 120	UltraScan	50 kX	4.8	0.67	40.87	-45° to +45°	61	-45° to 45°	0.312	13.00	102.4	55.0	123.8	94.8	155.9	S. Fig. 14
26	EMD-7245	Zeiss 120	UltraScan	50 kX	4.8	0.68	41.35	-45° to +45°	61	-45° to 45°	0.320	11.72	110.2	67.1	160.1	148.1	218.1	S. Fig. 14
27	EMD-7248	Zeiss 120	UltraScan	50 kX	4.8	1.72	105.09	-45° to +45°	61	-45° to 39°	0.315	9.87	102.8	63.2	131.2	112.2	172.7	S. Fig. 15
28	EMD-7251	Zeiss 120	UltraScan	50 kX	4.8	1.02	62.48	-45° to +45°	61	-43.5° to 45°	0.208	7.32	109.9	69.1	163.2	156.4	226.0	S. Fig. 15
29	EMD-7253	Zeiss 120	UltraScan	50 kX	4.8	1.69	103.02	-45° to +45°	61	-45° to 45°	0.290	10.05	123.5	55.8	169.9	162.8	235.3	S. Fig. 16
30	EMD-7255	Zeiss 120	UltraScan	50 kX	4.8	2.73	166.68	-45° to +45°	61	-45° to 45°	0.331	9.24	128.9	51.1	178.8	177.1	251.7	S. Fig. 16

31	EMD-7259	Zeiss 120	UltraScan	50 kX	4.8	1.74	106.19	-45° to +45°	61	-45° to 45°	0.270	9.48	124.8	50.7	151.2	131.4	200.3	S. Fig. 17
32	EMD-7264	Zeiss 120	UltraScan	50 kX	4.8	1.74	106.38	-45° to +45°	61	-43.5° to 45°	0.285	10.32	119.8	60.3	174.3	171.3	244.4	S. Fig. 17
33	EMD-7267	Zeiss 120	UltraScan	50 kX	4.8	1.04	63.15	-45° to +45°	61	-45° to 45°	0.328	10.71	108.3	61.7	140.8	120.8	185.6	S. Fig. 18
34	EMD-7270	Zeiss 120	UltraScan	50 kX	4.8	0.95	57.84	-45° to +45°	61	-45° to 45°	0.321	9.78	108.5	70.4	162.6	156.7	225.8	S. Fig. 18
35	EMD-7273	Zeiss 120	UltraScan	50 kX	4.8	0.87	52.86	-45° to +45°	61	-45° to 45°	0.295	10.45	110.1	54.6	134.0	109.2	172.8	S. Fig. 19
36	EMD-7276	Zeiss 120	UltraScan	50 kX	4.8	1.01	61.72	-45° to +45°	61	-45° to 45°	0.265	10.45	133.3	46.8	177.1	171.2	246.4	S. Fig. 19
37	EMD-7283	Zeiss 120	UltraScan	50 kX	4.8	1.01	61.36	-45° to +45°	61	-45° to 45°	0.296	10.86	131.3	47.8	168.2	157.8	230.7	S. Fig. 20
38	EMD-7285	Zeiss 120	UltraScan	50 kX	4.8	1.01	61.37	-45° to +45°	61	-45° to 45°	0.292	10.33	120.9	59.0	175.3	174.2	247.1	S. Fig. 20
39	EMD-7284	Zeiss 120	UltraScan	50 kX	4.8	1.75	106.64	-45° to +45°	61	-45° to 45°	0.323	10.67	91.8	65.7	117.1	99.7	153.8	S. Fig. 21
40	EMD-7282	Zeiss 120	UltraScan	50 kX	4.8	1.00	60.73	-45° to +45°	61	-43.5° to 42°	0.265	7.54	124.0	53.6	156.8	148.6	216.0	S. Fig. 21
41	EMD-7168	Zeiss 120	UltraScan	50 kX	4.8	1.91	116.46	-45° to +45°	61	-45° to 45°	0.323	8.00	131.1	46.3	166.4	143.4	219.7	S. Fig. 22
42	EMD-7173	Zeiss 120	UltraScan	50 kX	4.8	1.92	117.36	-45° to +45°	61	-45° to 45°	0.326	7.02	95.6	71.4	130.7	119.9	177.4	S. Fig. 22
43	EMD-7177	Zeiss 120	UltraScan	50 kX	4.8	1.84	112.41	-45° to +45°	61	-24° to 45°	0.306	9.89	112.4	64.2	157.5	145.0	214.1	S. Fig. 23
44	EMD-7180	Zeiss 120	UltraScan	50 kX	4.8	1.73	105.27	-45° to +45°	61	-45° to 45°	0.329	10.38	101.1	78.6	170.0	169.2	239.9	S. Fig. 23
45	EMD-7182	Zeiss 120	UltraScan	50 kX	4.8	2.30	140.36	-45° to +45°	61	-42° to 45°	0.381	10.41	120.9	58.5	164.8	164.4	232.8	S. Fig. 24
46	EMD-7185	Zeiss 120	UltraScan	50 kX	4.8	2.30	140.34	-45° to +45°	61	-45° to 45°	0.268	9.77	105.3	67.3	146.7	131.5	197.0	S. Fig. 24
47	EMD-7187	Zeiss 120	UltraScan	50 kX	4.8	2.28	139.05	-45° to +45°	61	-45° to 45°	0.346	11.80	102.6	74.2	156.1	149.5	216.2	S. Fig. 25
48	EMD-7190	Zeiss 120	UltraScan	50 kX	4.8	1.27	77.74	-45° to +45°	61	-45° to 45°	0.369	10.32	120.9	58.1	166.9	158.5	230.2	S. Fig. 25
49	EMD-7216	Zeiss 120	UltraScan	50 kX	4.8	1.30	79.04	-45° to +45°	61	-45° to 45°	0.381	9.22	137.0	41.8	166.1	148.5	222.8	S. Fig. 26
50	EMD-7198	Zeiss 120	UltraScan	50 kX	4.8	1.05	64.21	-45° to +45°	61	-36° to 42°	0.151	7.00	97.5	74.5	140.9	135.0	195.1	S. Fig. 26
51	EMD-7213	Zeiss 120	UltraScan	50 kX	4.8	1.08	65.74	-45° to +45°	61	-36° to 28.5°	0.243	8.03	113.6	65.3	166.0	161.4	231.5	S. Fig. 27
52	EMD-7220	Zeiss 120	UltraScan	50 kX	4.8	0.96	58.32	-45° to +45°	61	-45° to 42°	0.252	9.32	114.3	60.3	144.1	133.4	196.4	S. Fig. 27
53	EMD-7223	Zeiss 120	UltraScan	50 kX	4.8	1.66	101.02	-45° to +45°	61	-33° to 42°	0.240	6.26	127.9	34.8	121.6	87.4	149.8	S. Fig. 28
54	EMD-7226	Zeiss 120	UltraScan	50 kX	4.8	1.68	102.72	-45° to +45°	61	-34.5° to 30°	0.278	7.84	103.5	67.1	142.0	121.1	186.6	S. Fig. 28
55	EMD-7229	Zeiss 120	UltraScan	50 kX	4.8	1.69	102.91	-45° to +45°	61	-33° to 45°	0.201	10.87	91.1	88.2	168.0	167.2	237.0	S. Fig. 29
56	EMD-7232	Zeiss 120	UltraScan	50 kX	4.8	2.99	182.09	-45° to +45°	61	-45° to 45°	0.313	6.25	129.3	50.6	173.5	168.5	241.9	S. Fig. 29
57	EMD-7234	Zeiss 120	UltraScan	50 kX	4.8	2.56	156.27	-45° to +45°	61	-43.5° to 45°	0.255	6.10	111.7	60.8	141.1	125.6	188.9	S. Fig. 30
58	EMD-7237	Zeiss 120	UltraScan	50 kX	4.8	2.84	173.49	-45° to +45°	61	-45° to 45°	0.270	6.78	112.9	59.5	147.5	128.9	195.9	S. Fig. 30
59	EMD-7239	Zeiss 120	UltraScan	50 kX	4.8	2.59	157.73	-45° to +45°	61	-45° to 45°	0.255	5.74	106.1	70.3	147.0	142.7	204.9	S. Fig. 31
60	EMD-7242	Zeiss 120	UltraScan	50 kX	4.8	2.51	153.35	-45° to +45°	61	-45° to 45°	0.349	6.59	105.9	74.2	172.1	171.3	242.8	S. Fig. 31
61	EMD-7244	Zeiss 120	UltraScan	50 kX	4.8	2.37	144.64	-45° to +45°	61	-45° to 45°	0.278	6.90	105.9	72.0	165.0	153.0	225.0	S. Fig. 32
62	EMD-7247	Zeiss 120	UltraScan	50 kX	4.8	2.70	164.79	-45° to +45°	61	-27° to 45°	0.219	5.84	95.4	47.3	112.3	73.0	133.9	S. Fig. 32

63	EMD-7250	Zeiss 120	UltraScan	50 kX	4.8	2.53	154.49	-45° to +45°	61	-45° to 45°	0.301	6.28	78.9	68.2	99.4	91.2	134.9	S. Fig. 33
64	EMD-7252	Zeiss 120	UltraScan	50 kX	4.8	2.69	164.36	-45° to +45°	61	-45° to 45°	0.368	9.33	123.0	57.0	174.5	173.5	246.1	S. Fig. 33
65	EMD-7254	Zeiss 120	UltraScan	50 kX	4.8	2.66	162.04	-45° to +45°	61	-45° to 45°	0.286	5.55	118.0	51.8	146.2	114.6	185.8	S. Fig. 34
66	EMD-7256	Zeiss 120	UltraScan	50 kX	4.8	2.00	122.03	-45° to +45°	61	-45° to 45°	0.270	6.51	100.2	59.8	126.0	100.3	161.0	S. Fig. 34
67	EMD-7257	Zeiss 120	UltraScan	50 kX	4.8	1.49	90.67	-45° to +45°	61	-43.5° to 45°	0.236	10.67	106.5	68.2	151.4	138.8	205.4	S. Fig. 35
68	EMD-7260	Zeiss 120	UltraScan	50 kX	4.8	1.56	94.96	-45° to +45°	61	-43.5° to 45°	0.236	10.48	124.8	48.7	152.4	115.9	191.5	S. Fig. 35
69	EMD-7262	Zeiss 120	UltraScan	50 kX	4.8	1.93	117.65	-45° to +45°	61	-45° to 45°	0.291	9.36	118.3	27.9	124.7	56.2	136.8	S. Fig. 36
70	EMD-7265	Zeiss 120	UltraScan	50 kX	4.8	2.01	122.87	-45° to +45°	61	-45° to 45°	0.260	9.20	88.3	54.5	102.0	80.4	129.9	S. Fig. 36
71	EMD-7266	Zeiss 120	UltraScan	50 kX	4.8	2.08	126.71	-45° to +45°	61	-45° to 45°	0.226	9.92	107.5	55.9	129.1	104.6	166.1	S. Fig. 37
72	EMD-7269	Zeiss 120	UltraScan	50 kX	4.8	1.95	119.19	-45° to +45°	61	-45° to 45°	0.383	7.83	111.5	68.2	171.2	169.0	240.6	S. Fig. 37
73	EMD-7271	Zeiss 120	UltraScan	50 kX	4.8	1.66	100.99	-45° to +45°	61	-45° to 45°	0.341	7.72	91.0	50.8	104.0	74.9	128.2	S. Fig. 38
74	EMD-7272	Zeiss 120	UltraScan	50 kX	4.8	1.72	104.72	-45° to +45°	61	-45° to 45°	0.284	9.42	114.8	62.7	150.6	144.0	208.4	S. Fig. 38
75	EMD-7275	Zeiss 120	UltraScan	50 kX	4.8	1.61	98.47	-45° to +45°	61	-33° to 45°	0.358	7.36	114.2	57.9	144.8	125.1	191.3	S. Fig. 39
76	EMD-7277	Zeiss 120	UltraScan	50 kX	4.8	1.57	95.97	-45° to +45°	61	-45° to 45°	0.307	7.86	107.0	60.0	137.9	113.9	178.9	S. Fig. 39
77	EMD-7279	Zeiss 120	UltraScan	50 kX	4.8	1.60	97.89	-45° to +45°	61	-45° to 45°	0.242	6.15	52.0	35.9	64.1	44.7	78.1	S. Fig. 40
78	EMD-7281	Zeiss 120	UltraScan	50 kX	4.8	1.57	95.78	-45° to +45°	61	-45° to 45°	0.301	9.27	100.8	68.6	135.9	124.3	184.2	S. Fig. 40
79	EMD-7280	Zeiss 120	UltraScan	50 kX	4.8	1.46	88.95	-45° to +45°	61	-45° to 45°	0.353	7.58	88.5	88.1	154.0	149.0	214.3	S. Fig. 41
80	EMD-7278	Zeiss 120	UltraScan	50 kX	4.8	1.53	93.15	-45° to +45°	61	-45° to 42°	0.281	6.44	117.1	60.3	161.0	147.7	218.5	S. Fig. 41
81	EMD-7188	Zeiss 120	UltraScan	50 kX	4.8	0.68	41.64	-45° to +45°	61	-45° to 45°	0.378	13.11	105.7	41.5	115.4	69.8	134.9	S. Fig. 42
82	EMD-7191	Zeiss 120	UltraScan	50 kX	4.8	1.30	79.03	-45° to +45°	61	-45° to 45°	0.303	12.21	105.6	66.2	142.6	124.2	189.2	S. Fig. 42
83	EMD-7192	Zeiss 120	UltraScan	50 kX	4.8	0.70	42.46	-45° to +45°	61	-45° to 45°	0.322	12.75	113.8	35.1	122.8	65.8	139.3	S. Fig. 43
84	EMD-7195	Zeiss 120	UltraScan	50 kX	4.8	0.67	40.67	-45° to +45°	61	-45° to 25.5°	0.320	11.88	113.7	65.5	167.0	162.1	232.7	S. Fig. 43
85	EMD-7197	Zeiss 120	UltraScan	50 kX	4.8	0.58	35.51	-45° to +45°	61	-45° to 45°	0.282	12.31	93.7	64.7	109.7	89.5	141.5	S. Fig. 44
86	EMD-7199	Zeiss 120	UltraScan	50 kX	4.8	0.67	40.86	-45° to +45°	61	-45° to 45°	0.347	13.69	108.3	70.9	166.4	163.2	233.1	S. Fig. 44
87	EMD-7202	Zeiss 120	UltraScan	50 kX	4.8	0.71	43.42	-45° to +45°	61	-45° to 45°	0.314	12.71	113.1	66.8	173.7	172.8	245.0	S. Fig. 45
88	EMD-7204	Zeiss 120	UltraScan	50 kX	4.8	1.76	107.50	-45° to +45°	61	-45° to 43.5°	0.298	10.83	117.6	54.8	147.8	125.3	193.8	S. Fig. 45
89	EMD-7206	Zeiss 120	UltraScan	50 kX	4.8	1.71	104.09	-45° to +45°	61	-45° to 45°	0.306	9.97	113.8	66.5	163.4	155.9	225.9	S. Fig. 46
90	EMD-7208	Zeiss 120	UltraScan	50 kX	4.8	1.77	107.86	-45° to +45°	61	-45° to 45°	0.239	10.00	75.5	69.7	103.3	87.1	135.1	S. Fig. 46
91	EMD-7209	Zeiss 120	UltraScan	50 kX	4.8	1.00	60.87	-45° to +45°	61	-45° to 45°	0.386	10.52	104.7	75.5	173.5	172.2	244.5	S. Fig. 47
92	EMD-7211	Zeiss 120	UltraScan	50 kX	4.8	1.03	62.59	-45° to +45°	61	-45° to 45°	0.364	10.82	110.7	69.2	170.0	168.7	239.5	S. Fig. 47
93	EMD-7212	Zeiss 120	UltraScan	50 kX	4.8	1.03	62.75	-45° to +45°	61	-45° to 45°	0.330	10.64	116.3	25.6	120.9	48.8	130.4	S. Fig. 48
94	EMD-7215	Zeiss 120	UltraScan	50 kX	4.8	1.05	64.31	-45° to +45°	61	-45° to 45°	0.357	11.31	116.6	62.9	167.8	161.9	233.2	S. Fig. 48

95	EMD-7218	Zeiss 120	UltraScan	50 kX	4.8	1.76	107.26	-45˚ to +45˚	61	-45˚ to 45˚	0.285	10.51	89.6	51.1	99.7	69.5	121.6	S. Fig. 49
96	EMD-7221	Zeiss 120	UltraScan	50 kX	4.8	1.76	107.18	-45˚ to +45˚	61	-45˚ to 45˚	0.325	10.23	99.9	37.5	110.4	56.8	124.2	S. Fig. 49
97	EMD-7222	Zeiss 120	UltraScan	50 kX	4.8	1.75	106.85	-45˚ to +45˚	61	-45˚ to 45˚	0.359	10.30	109.4	66.6	154.9	142.8	210.7	S. Fig. 50
98	EMD-7225	Zeiss 120	UltraScan	50 kX	4.8	2.32	141.74	-45˚ to +45˚	61	-45˚ to 45˚	0.166	9.58	101.8	51.2	117.7	83.6	144.4	S. Fig. 50
99	EMD-7227	Zeiss 120	UltraScan	50 kX	4.8	1.79	109.08	-45˚ to +45˚	61	-45˚ to 45˚	0.378	10.09	115.7	64.3	167.2	162.1	232.9	S. Fig. 51
100	EMD-7230	Zeiss 120	UltraScan	50 kX	4.8	1.87	113.92	-45˚ to +45˚	61	-45˚ to 45˚	0.371	9.60	112.4	66.5	151.5	150.3	213.5	S. Fig. 51
101	EMD-7233	Zeiss 120	UltraScan	50 kX	4.8	1.88	114.92	-45˚ to +45˚	61	-45˚ to 45˚	0.320	10.56	104.0	58.3	126.8	102.4	163.0	S. Fig. 52
102	EMD-7236	Zeiss 120	UltraScan	50 kX	4.8	1.82	111.11	-45˚ to +45˚	61	-45˚ to 37.5˚	0.303	7.89	120.4	59.7	179.1	178.7	253.0	S. Fig. 52
103	EMD-7238	Zeiss 120	UltraScan	50 kX	4.8	1.93	117.67	-45˚ to +45˚	61	-45˚ to 45˚	0.360	8.48	113.2	67.1	174.5	171.7	244.8	S. Fig. 53
104	EMD-7241	Zeiss 120	UltraScan	50 kX	4.8	1.95	118.90	-45˚ to +45˚	61	-45˚ to 45˚	0.270	10.49	74.7	37.5	77.5	45.7	90.0	S. Fig. 53
105	EMD-7243	Zeiss 120	UltraScan	50 kX	4.8	2.28	139.27	-45˚ to +45˚	61	-45˚ to 45˚	0.311	10.07	104.3	34.9	100.3	38.2	107.4	S. Fig. 54
106	EMD-7246	Zeiss 120	UltraScan	50 kX	4.8	2.27	138.60	-45˚ to +45˚	61	-45˚ to 45˚	0.309	10.84	112.6	38.6	123.2	71.4	142.4	S. Fig. 54
107	EMD-7249	Zeiss 120	UltraScan	50 kX	4.8	2.27	138.36	-45˚ to +45˚	61	-45˚ to 45˚	0.302	10.34	102.3	72.2	145.5	132.7	196.9	S. Fig. 55
108	EMD-7258	Zeiss 120	UltraScan	50 kX	4.8	2.28	138.79	-45˚ to +45˚	61	-45˚ to 45˚	0.314	10.61	92.2	54.7	104.6	77.4	130.2	S. Fig. 55
109	EMD-7261	Zeiss 120	UltraScan	50 kX	4.8	1.23	75.07	-45˚ to +45˚	61	-45˚ to 45˚	0.324	9.85	111.8	59.8	144.7	125.0	191.2	S. Fig. 56
110	EMD-7263	Zeiss 120	UltraScan	50 kX	4.8	1.17	71.66	-45˚ to +45˚	61	-45˚ to 45˚	0.344	10.00	113.7	58.3	147.2	123.0	191.8	S. Fig. 56
111	EMD-7268	Zeiss 120	UltraScan	50 kX	4.8	1.40	89.39	-46.5˚ to +48˚	64	-46.5˚ to 39˚	0.138	6.74	95.9	51.3	104.2	66.6	123.7	S. Fig. 57
112	EMD-7274	Zeiss 120	UltraScan	50 kX	4.8	1.39	89.11	-46.5˚ to +48˚	64	-45˚ to 45˚	0.243	8.55	115.2	64.7	174.8	172.3	245.4	S. Fig. 57
113	EMD-7159	FEI TF20	K2 Summit	19 kX	3.7	0.69	44.65	-48˚ to +48˚	65	-25.5˚ to 48˚	0.017	12.01	123.5	54.9	166.0	153.3	226.0	S. Fig. 58
114	EMD-7160	FEI TF20	K2 Summit	19 kX	3.7	0.70	45.67	-48˚ to +48˚	65	-25.5˚ to 48˚	0.010	10.99	121.2	58.8	177.1	175.6	249.4	S. Fig. 58
115	EMD-7161	FEI TF20	K2 Summit	19 kX	3.7	0.67	43.33	-48˚ to +48˚	65	-25.5˚ to 48˚	0.017	11.43	92.5	32.1	92.2	45.9	103.0	S. Fig. 59
116	EMD-7162	FEI TF20	K2 Summit	19 kX	3.7	0.69	44.84	-48˚ to +48˚	65	-48˚ to 40.5˚	0.009	9.90	101.1	67.5	136.2	121.0	182.2	S. Fig. 59
117	EMD-7163	FEI TF20	K2 Summit	19 kX	3.7	0.67	43.64	-48˚ to +48˚	65	-48˚ to 40.5˚	0.015	11.02	73.6	54.4	84.5	69.2	109.2	S. Fig. 60
118	EMD-7164	FEI TF20	K2 Summit	19 kX	3.7	0.68	44.19	-48˚ to +48˚	65	-48˚ to 40.5˚	0.010	11.37	119.9	60.7	178.3	174.7	249.6	S. Fig. 60
119	EMD-7166	FEI TF20	K2 Summit	19 kX	3.7	0.71	46.04	-48˚ to +48˚	65	-48˚ to 40.5˚	0.013	8.75	102.4	74.0	153.2	145.3	211.1	S. Fig. 61
120	EMD-7167	FEI TF20	K2 Summit	19 kX	3.7	0.69	44.73	-48˚ to +48˚	65	-48˚ to 40.5˚	0.015	9.91	107.7	72.2	167.5	161.0	232.3	S. Fig. 61
121	EMD-7169	FEI TF20	K2 Summit	19 kX	3.7	0.69	44.64	-48˚ to +48˚	65	-48˚ to 40.5˚	0.016	9.75	100.2	77.9	158.8	155.9	222.6	S. Fig. 62
122	EMD-7171	FEI TF20	K2 Summit	19 kX	3.7	0.67	43.61	-48˚ to +48˚	65	-48˚ to 40.5˚	0.009	10.90	108.9	64.1	141.7	130.4	192.6	S. Fig. 62
123	EMD-7172	FEI TF20	K2 Summit	19 kX	3.7	0.67	43.24	-48˚ to +48˚	65	-48˚ to 40.5˚	0.015	9.88	102.2	51.1	118.9	83.7	145.5	S. Fig. 63
124	EMD-7174	FEI TF20	K2 Summit	19 kX	3.7	0.66	43.13	-48˚ to +48˚	65	-48˚ to 40.5˚	0.016	8.91	87.2	11.4	87.1	10.6	87.7	S. Fig. 63
125	EMD-7176	FEI TF20	K2 Summit	19 kX	3.7	0.69	44.93	-48˚ to +48˚	65	-48˚ to 48˚	0.019	8.80	69.4	52.8	79.0	64.1	101.7	S. Fig. 64
126	EMD-7178	FEI TF20	K2 Summit	19 kX	3.7	0.65	42.12	-48˚ to +48˚	65	-48˚ to 30˚	0.008	11.89	57.2	46.1	62.1	52.4	81.3	S. Fig. 64

#	EMDB#[1]	TEM[2]	CCD[3]	Mag.[3]	Apix[4] (Å)	Dose/img.[5] (e-/Å2)	Dose/set[6] (e-/Å2)	Acq. angle range[7]	Total img.[8]	Reconst. angle range[9]	Cont.[10]	Resol.[11] (nm)	α (°)	β (°)	ϑ (°)	φ (°)	RSS of ang.[12] (°)	Supp. Fig. [13]
127	EMD-7179	FEI TF20	K2 Summit	19 kX	3.7	0.65	42.13	-48° to +48°	65	-48° to 48°	0.009	12.32	75.7	56.6	90.7	71.6	115.5	S. Fig. 65
128	EMD-7183	FEI TF20	K2 Summit	19 kX	3.7	0.64	41.31	-48° to +48°	65	-48° to 48°	0.012	10.01	57.4	18.6	69.4	6.0	69.6	S. Fig. 65
129	EMD-7186	Zeiss 120	UltraScan	50 kX	4.8	1.89	115.44	-45° to +45°	61	-45° to 45°	0.339	8.48	84.8	51.6	124.5	71.0	143.3	S. Fig. 66

[1] EMDB Index: https://www.ebi.ac.uk/pdbe/emdb/

[2] TEM model: FEI TF20 stands for FEI TF200 TEM; Zeiss 120 stands for Zeiss Libra 120 Plus TEM

[3] CCD: K_2 Summit stands for Gantan K_2 Summit Direct Detector; UltraScan for Gatan UltraScan 4000 4Kx4K CCD

[4] Angstrom per pixel

[5] Dose used for each CCD frame

[6] Dose used for whole tilt series

[7] Data acquisition angle range

[8] Total images in the tilt series

[9] Reconstruction angle range

[10] Contour used for display

[11] IPET 3D reconstruction resolution

[12] RSS: Root sum squared angles of ϑ and φ, *i.e.* $(\vartheta^2 + \varphi^2)^{1/2}$

[13] Supplementary Figure showed the detailed process of IPET 3D reconstruction
